# Solar Flow Synthesis of Polymer Nanoparticles: Scaling Local Experiments to Global Potential

**DOI:** 10.1002/anie.202524746

**Published:** 2026-02-03

**Authors:** Jochen A. Kammerer, Joshua O. Holloway, Theresa Stephan, Hartmut Gliemann, Florian Feist, Fred Pashley‐Johnson, Laura Delafresnaye, Christopher Barner‐Kowollik

**Affiliations:** ^1^ Soft Matter Materials Laboratory School of Chemistry and Physics Queensland University of Technology (QUT) Brisbane Queensland Australia; ^2^ Centre For Materials Science Queensland University of Technology (QUT) Brisbane Queensland Australia; ^3^ Institute of Functional Interfaces (IFG) Karlsruhe Institute of Technology (KIT) Eggenstein‐Leopoldshafen Germany; ^4^ Polymer Chemistry Research Group Centre of Macromolecular Chemistry (CMaC) Department of Organic and Macromolecular Chemistry Faculty of Sciences Ghent University Ghent Belgium

**Keywords:** flow chemistry, photochemistry, polymer nanoparticles, solar photochemistry

## Abstract

We present the scalable, additive‐free synthesis of polymer nanoparticles in continuous flow, using solely solar radiation. Using a custom‐made flow reactor, the UV radiation from the sun induces a Diels–Alder step‐growth polymerization between a bismaleimide and a difunctional *o*‐methylbenzaldehyde. The resulting photopolymer subsequently precipitates as nanoparticles without the need for any additional additives, stimuli or processing steps. The solar flow reactor was designed by first carefully assessing the underpinning photochemistry of the photo‐induced Diels–Alder reaction using photochemical action plots and then performing a kinetic investigation of the particle formation under solar irradiation. The determined kinetics allow us to extrapolate our experimental results to a worldwide particle yield by using global UV index data, validated by two highly different geographical locations, Australia and Germany. Our results clearly demonstrate the applicability of our system for the scalable, sustainable, solar‐powered production of polymeric nanoparticles in regions of high levels of solar radiation. Furthermore, our calculations function as a blueprint for how local experimental data can be extrapolated to assess the global solar photochemical potential of photochemical systems, thus making their performance comparable.

## Graphical abstract

The large‐scale sunlight‐induced photochemical synthesis of polymer nanoparticles based on the Diels–Alder step‐growth polymerization of a bismaleimide and a difunctional *o*‐methylbenzaldehyde in a self‐built solar flow reactor is introduced. Furthermore, a framework is established to translate sunlight‐driven experimental results globally—validated in both the southern and northern hemisphere—making sun‐induced syntheses and solar reactor designs more comparable.

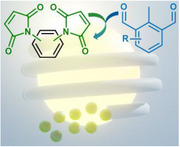

## 1 Introduction

In 1912, Ciamician made his now famous speech, that “on the arid lands there will spring up industrial colonies without smoke and without smokestacks; forests of glass tubes will extend over the plains and glass buildings will rise everywhere; inside of these will take place the photochemical processes that hitherto have been the guarded secret of the plants, but that will have been mastered by human industry which will know how to make them bear even more abundant fruit than nature, for nature is not in a hurry and mankind is. And if in a distant future the supply of coal becomes completely exhausted, civilisation will not be checked by that, for life and civilisation will continue as long as the sun shines!” [1]. Surprisingly—despite his undoubtably valid assertion—humankind has unfortunately made only limited progress in exploiting solar radiation for the production of chemicals and materials, instead continuing with the fossil fuel industry that Ciamician predicted would one day become exhausted [2]. While solar radiation is used globally, to varying degrees, in electricity generation and water heating, there is—to the best of our knowledge—not a single commercially viable chemical production process in place that relies on sunlight.

Over the last decade, photochemistry has begun to undergo a precision transformation, underpinned by the paradigm that the absorption spectra of chromophores can bepoor predictors of photochemical reactivity at specific wavelengths, as shown in many examples [3, 4]. This understanding of the relationship between chromophores and the optimum wavelength for photochemical reactivity has been enabled by the development of so‐called modern‐day photochemical action plots. These action plots analyse the wavelength‐resolved reactivity of a chromophore, wavelength by wavelength, depositing a defined and identical number of photons from a tunable laser system at each examined wavelength [3, 4, 5, 6, 7]. The resulting observations have revealed that significant and sometimes maximum photochemical reactivity can exist well beyond the absorption maximum and into the red‐shifted region [8]. These findings suggest that photochemistry in previously non‐explored wavelength regimes—including those provided by solar radiation—is possible. In other words, even regions of low absorptivity can lead to high reactivities, enabling potentially high penetration depth and maximum yields. While these advantages, enabled by the information contained in photochemical action plots, are important considerations when conducting photochemistry in bulk systems, they become even more powerful in flow chemical setups [9]. Photo‐flow chemistry is ideally suited not only for homogenous solutions [10, 11, 12, 13, 14], but more critically for conducting photochemistry in systems that evolve intense scattering profiles with increasing reaction progress, such as self‐assembled polymer structures and nano‐ and microparticle formation, as it circumvents some of the problems usually associated with light scattering and penetration depth when irradiating heterogeneous systems [9, 15, 16].

**FIGURE 1:**
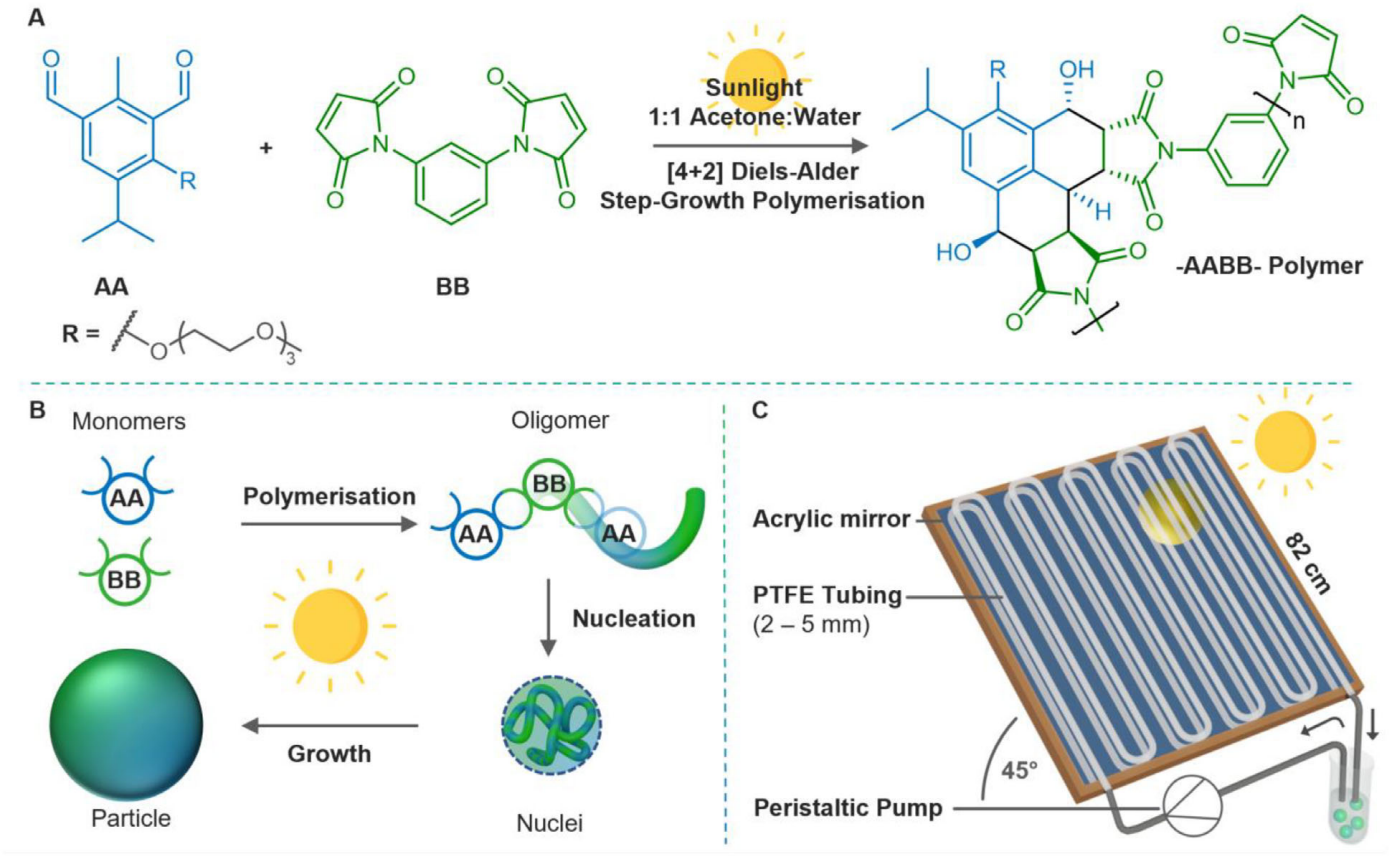
Solar flow synthesis of polymeric nanoparticles via [4+2] Diels–Alder step‐growth photopolymerisation. (A) The photoactive AA monomer reacts with the BB monomer through a [4+2] Diels–Alder reaction after UV‐promoted photoisomerisation to the photoenol upon solar irradiation. (B) Particle formation via precipitation polymerisation: As the step‐growth polymerisation of the AA and BB monomers proceeds, the growing oligomers reach their critical molecular weight for solubility, leading to the nucleation of particles. These nuclei grow to stable nanoparticles by capturing additional oligomers that reach their solubility threshold. (C) Design of the solar flow reactor for sunlight‐induced nanoparticle synthesis: The monomer solution is circulated within PTFE tubing. The reaction solution is exposed to the sun in the white parts of the tubing in the diagram and shielded from radiation in the black parts and the reservoir. Circulating the solution allows us to choose the residence time independent of the flow speed and the small reservoir in the circuit enables the extraction of samples during the experiment. A mirror behind the tubing increases the radiation intensity. The entire setup is angled toward the sun at 45°.

The synthesis of polymeric nano‐ and micro‐particles is of key industrial importance, given their use in a wide range of applications, including point‐of‐care diagnostic tests (such as COVID‐19 [17] or early detection of myocardial infarction) [18], coatings or drug delivery systems, and high surface area materials for flow separation systems such as size‐exclusion chromatography (SEC) [9, 19, 20, 21, 22, 23, 24]. The production of polymeric particles has, in the past, been largely accomplished using thermal fabrication methods in dispersed media [25, 26, 27], most notably the Stöver process [28] and related precipitation polymerisation methodologies from a θ‐solvent solution [29, 30, 31, 32, 33, 34, 35]. Alternatively, the production of polymer particles via light‐induced chemistries is more challenging given the evolving disperse nature of the reaction medium [36]. However, it holds critical advantages, such as ambient temperature synthesis and superior spatiotemporal control [9, 22, 36, 37, 38, 39, 40, 41]. Our team has introduced methodologies that enable the synthesis of polymer particles from polymer strands that are decorated with photoreactive units—such as tetrazoles [42], *o*‐methyl benzaldehydes [43, 44], and triazolinediones [45]. Interestingly, the resulting polymeric microparticles retain photoreactive functionalities on their surface, which can be later employed for further functionalisation [22, 43, 44]. More recently, we have introduced a simplified approach to prepare polymeric nano‐ and microparticles via a photoinduced step‐growth polymerisation, using a difunctional *o*‐methylbenzaldehyde (AA, Figure 1A) and a bismaleimide species (BB, Figure 1A), which requires no additives or surfactants and has been shown to operate in both batch and photo‐flow reactors [9, 22, 46]. This simple system thus lends itself extremely well to be translated into a solar‐driven flow process. Additionally, the particles produced through the photo‐induced step‐growth polymerisation are of AA‐ and BB‐type monomers and can be readily surface‐modified to alter the dispersion properties or to introduce functionality [9, 22, 46]. Several studies have explored the use of cost‐effective “solar flow” for organic photochemistry with various reactor designs [2, 47, 48, 49, 50]. However, the different levels of solar irradiance based on latitude, season, and weather influences during the experiments make the obtained results generally incomparable.

In the current contribution, we fuse the findings of precision photochemistry and light‐driven polymeric nano‐ and microparticle formation with solar‐powered flow chemistry to establish the production of polymeric nanoparticles in a sustainable, scalable fashion. Figure 1A,B depicts the chemical reaction system and particle formation for which we designed the solar flow reactor in Figure 1C. We used this solar flow reactor in Brisbane, Australia and in Karlsruhe, Germany (both independently constructed at each location but with identical design specifications; for details on the construction of the reactors refer to the Sections S1 and S2, respectively) to investigate the influence of the tubing diameter and the flow speed on the total particle yield as well as the particle yield over time. Furthermore, we monitor the particle growth as a function of sun exposure time and UV light intensity. These UV light intensity dependent kinetics allow us to translate our local experimental nanoparticle yield to the yield of a hypothetical chemical plant that uses our reactor design anywhere in the world. Despite the simple reactor design, our data clearly demonstrates the viability of our chemical system for the sustainable, large‐scale manufacturing of polymeric nanoparticles using sunlight. We furthermore hope to encourage other researchers to explore the power of the sun for other photochemical reactions and propose a framework to make solar photochemical investigations globally comparable.

## 2 Results and Discussion

### 2.1 Design of the Solar Flow Reactor

**FIGURE 2:**
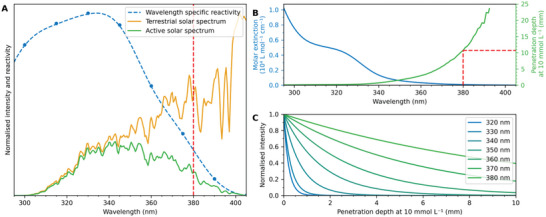
Design considerations for the solar flow reactor. (A) The wavelength‐specific reactivity (blue) of the monofunctional model system [51] of the Diels–Alder photocycloaddition determines—together with the terrestrial solar spectrum [52] (orange)—the active solar spectrum (green) for the nanoparticle synthesis. The active solar spectrum shows that only photons with a wavelength below 380 nm significantly contribute to the photocycloaddition (red‐dashed line). For the calculation of the active solar spectrum, the measured wavelength‐specific reactivity (blue dots) was spline interpolated (blue‐dashed line) and multiplied with the solar spectrum. (B) From the molar extinction coefficient of the reaction solution (blue), the penetration depths of the relevant photons (green) can be estimated to about 10 mm at a concentration of 10 mmol L^−1^ (red‐dashed lines) using Beer–Lambert's law. (C) Photons of shorter wavelengths have a lower penetration depth into the solution, as calculated from the molar extinction in B using Beer–Lambert's law and a concentration of 10 mmol L^−1^.

The design of the solar flow reactor (Figures 1C and S1) is dictated by the photochemistry and physical properties of the reaction solution in combination with the terrestrial solar spectrum (Figure 2A). To evaluate the photochemistry, we used the wavelength‐specific reactivity (inphotochemical action plot) of the Diels–Alder photocycloaddition of a monofunctional maleimide and a monofunctional *o*‐methylbenzaldehyde as a model system [51]. The product of the terrestrial solar spectrum [52] and the wavelength‐specific reactivity results in the active solar spectrum and shows that only photons with a wavelength of 320–380 nm significantly contribute to the photocycloaddition (Figure 2A). Below 320 nm, the number of photons limits the photocycloaddition despite the high wavelength‐specific reactivity, whereas above 380 nm, the low wavelength‐specific reactivity is limiting. To utilise as many photons of the relevant wavelengths as possible for the reaction, we selected polytetrafluoroethylene (PTFE) tubing with minimal extinction at the relevant wavelengths (refer to the extinction spectrum in Figure S1D) and positioned a mirror ca. 2 cm behind the tubing to increase the radiation intensity.

For the present system, a monomer concentration of 10 mmol L^−1^ in a 1:1 mixture of water and acetone is ideal for nanoparticle formation [9]. At this concentration, 380 nm photons penetrate about 10 mm into the reaction solution (refer to Figure 2B), rendering 10 mm as a maximum useful inner diameter for the tubing, past which the reaction solution is not fully irradiated anymore. Maximising the tubing diameter was one of our initial design considerations, as the throughput scales quadratically with the inner tubing radius (at constant flow speed).

We designed the solar flow reactor to circulate the reaction mixture in a closed loop system rather than using a fixed tube length, thus ensuring sufficient sun exposure to complete the reaction at any flow speed. Furthermore, we included a small reservoir into the circuit, which we used to extract samples from during the experiments. In addition, due to the variability that is inevitable when relying on solar irradiation in comparison to other typical reaction parameters such as pressure or temperature, the recirculation means that reaction duration can be extended indefinitely. In an industrialised version of the reactor presented here, we propose that real‐time monitoring of the actual solar irradiation would enable calculation of the optimum reaction time for a given size of particle. For the flow conditions presented in our current study, residence times and pressure‐drops associated with the reactor are provided in Section S12.

We note that the wavelength‐resolved activity we used for the design of our solar flow reactor was determined for a model system in a different solvent (*d*_3_‐acetonitrile), which can shift the wavelength specific reactivity [4, 53]. However, our following investigation of the influence of tubing diameter and flow speed shows that these parameters have—in conjunction with the wavelength‐specific penetration depths—a significant influence on the particle formation via the nucleation and growth of the nanoparticles, which could not be predicted from the wavelength‐specific reactivity alone.

### 2.2 Flow Speed and Tubing Diameter Affect the Particle Yield but Not the Particle Quality

To investigate the influence of the flow speed (the velocity of the reaction solution in the tubing) on the particle yield and quality, we varied the flow speed between almost 0 and 30 m min^−1^ in tubing with an inner diameter of 2, 3, and 5 mm. It should be noted that all data presented hereafter is strictly under laminar flow conditions, with Reynold's numbers of less than 2300 in all cases (Section S11).

Initially, the particles produced were analyzed by FT‐IR spectroscopy to confirm the proposed polymerisation mechanism shown in Figure 1 (Figures S107 and S108). The disappearance of the sharp C–H stretching band at 3103 cm^−1^ for the BB monomer and formation of a broad ─OH signal at 3465 cm^−1^ is consistent with the proposed mechanism. In addition, inspection of the 1600–1800 cm^−1^ region shows that the aldehyde C═O signal is completely consumed in the reaction, while the C═O of the maleimide is retained. Further analysis of the formation of the particles by ^1^H NMR spectroscopy under LED irradiation further confirmed these findings (refer to Section S10). These observations are in line with our previously determined formation mechanism for the particles [22].

**FIGURE 3:**
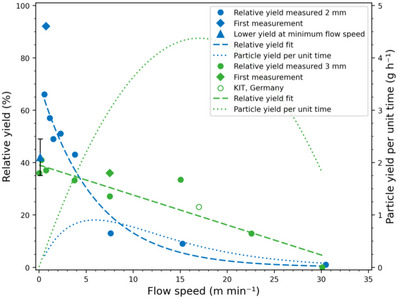
Yield as a function of flow speed for 2 and 3 mm tubing. A linear and exponential fit reflect the dependency of the yield on the flow rate for 2 and 3 mm, respectively (dashed lines). Diamond data points correspond to the first measurement with new tubing. For 2 mm, the yield decreases again when the flow speed approaches zero (triangular datapoint). The experiment was repeated four times and excluded from the fit. The error bar reflects the corresponding standard deviation. The particle yield per time (dotted lines) is calculated from the fits using Equation S5. The hollow data point was obtained with an identical reactor set‐up with 3 mm tubing in the northern hemisphere (Karlsruhe, Germany).

For both 2 and 3 mm tubing, we observed a decreasing particle yield with increasing flow speed (Figure 3). For the 2 mm tubing, this trend is broken when the flow speed converges to zero (Figure 3, blue triangular datapoint), where a slight decrease in yield is observed compared to flow speeds ≥1 m min^−1^. This is consistent with previous observations that some turbulent flow is necessary for efficient particle formation [9].

When we increased the tubing diameter to 5 mm, we only observed significant particle formation in the first run with virgin PTFE tubing and obtained a relative yield of 13% at a flow speed of 7.5 m min^−1^, where relative yield refers to the ratio of isolated particle mass with respect to the used monomer mass. In the second run at a lower flow speed of 3.8 m min^−1^, where a yield of 30%–40% could be obtained at 2 and 3 mm, we observed a relative particle yield of only 4%, opposing the otherwise observed trend of increasing particle yield with decreasing speed. A subsequent run at a higher flow rate of 15.1 m min^−1^ yielded a clear solution without any particle formation. Instead, polymer deposited as a thin, homogeneous layer onto the walls of the tubing. In fact, we also observed a significantly higher yield in the first run at 2 and 3 mm (Figure 3, diamond data points) as well as a build‐up of polymer deposit on the walls of the tubing over time, especially at higher flow rates. From these observations, we postulate the following mechanism of yield loss for our system: Particle formation occurs by nucleation and growth due to the decreasing solubility of the forming oligomers with increasing molecular weight (Figure 1B). In the initially clear solution, longer and longer oligomers form. Once they reach a critical molecular weight and concentration, nuclei form in the reaction solution, which subsequently grow into polymeric nanoparticles via the attachment of further oligomers, reaching their solubility limit upon chain extension [9].

During our initial design considerations, we inferred that photons of the relevant wavelengths penetrate up to 10 mm into the reaction solution (Figure 2B). Thus, particles should—in principle—form at a tubing diameter of 5 mm. However, the penetration depth of the photons drastically decreases with decreasing wavelength (Figure 2C). A diameter of 10 mm, which corresponds to the penetration depth of the active photons with 380 nm wavelength, was not tested due to the results at smaller diameters.

Thus, a large part of the photoreaction is confined to the vicinity of the PTFE tubing walls. The confinement of the reaction close to the tubing surface leads—in the case of larger diameter tubing—to a higher oligomer concentration and molecular weight close to the tubing surface and a lower oligomer concentration and molecular weight in the volume of the solution. Therefore, the critical concentration and molecular weight for nuclei formation is less likely to be reached in the solution volume. Instead, the oligomers that reach their solubility limit are preferably deposited onto the walls of the tubing, further depleting the reaction solution from oligomers.

At smaller diameters, the above‐described filter effect is less pronounced, as higher oligomer concentrations and molecular weights are reached in the volume of the solution, since photons of shorter wavelength penetrate most of the volume. This inner filter effect is also accelerated at higher flow speeds, where the deposition of the oligomers onto the tubing is preferred over the deposition onto the particles.

In general, reactor fouling is detrimental for industrial applications. However, we still achieved yields of 66% with the 2 mm tubing and the oligomer deposit can be easily removed mechanically, for example, by flushing cotton balls through the reactor. Furthermore, we achieved a yield of 92% in the first run with the 2 mm tubing. As much as the nucleation step is necessary for the particle formation, the polymer must also nucleate on the tubing walls before deposition becomes possible. Since this nucleation barrier needs to be overcome in the first run with new tubing, less oligomer is deposited onto the walls of the tubing and the relative particle yield is significantly higher. Thus, reactor fouling can be reduced—and particle yield drastically increased—with optimal surface engineering of the tubing.

Interestingly, we achieved the high relative yields of 66% and 92% despite the exposure of the reaction solution to air via the reservoir sample extraction (refer to Figure 1C). This is remarkable as oxygen quenches the reactive triplet state of the system [51].

**FIGURE 4:**
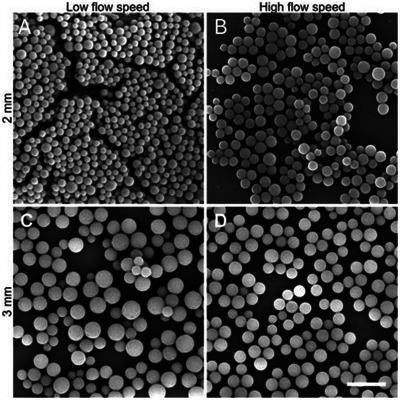
SEM images demonstrating that the particle roundness is not affected by the flow speed. (A) Flow speed: 0.57 m min^−1^ at 2 mm. (B) Flow speed: 15.1 m min^−1^ at 2 mm. (C) Flow speed: 0.26 m min^−1^ at 3 mm. (D) Flow speed: 15.1 m min^−1^ at 3 mm. Scale bar of 1 µm applies to all images. Note that the size difference between the samples is a result of the varying sun intensities during the experiments leading to different reaction speeds at a similar exposure time. The samples have similar particle sizes at completion of the reaction (refer to the kinetic investigation via dynamic light scattering (DLS) within our study). Refer to Section S5.3 for micrographs of all samples.

Despite the severe impact of tubing diameter and flow speed onto the particle yield, we did not observe a significant impact on the particle roundness (refer to exemplary scanning electron microscopy (SEM) images in Figure 4). The consistent roundness of particles is a result of the straight tubing of the solar flow reactor. Previously, we obtained a lower roundness for higher flow speeds with our laboratory flow reactor using an artificial light source [9]. Curled tubing leads to centripetal forces, which increase with increasing flow speed, and lead to concentration gradients and particle deformation and fusing [54, 55]. The straight tubing of our solar flow reactor removes this effect and allows us to produce high quality nanoparticles, as shown in Figure 4.

Even though the flow speed does not affect the roundness of the particles, it has a slight influence on the dispersity of the particles. From the SEM images in Figure 4, a larger diameter variance can be observed for the lower flow speeds. With increasing flow speed, the dispersity decreases from 0.046 to 0.014 for 2 mm and from 0.034 to 0.008 for 3 mm (refer to Section S5.3). Higher flow speeds lead to a better mixing of the reaction solution, which reduces the concentration and molecular weight gradients from the inequal solution irradiation, leading to more homogeneous particle growth. Despite the change of dispersity, a maximum dispersity of 0.046 can be still considered small.

To illustrate the general dependency of the relative particle yield and flow rate, we fitted an exponential and a linear function to the 2 and 3 mm yields, respectively (Figure 3). These simple functions do not have a physical meaning but describe the relationship between yield and flow speed well. From these fits, we calculated the yield per unit time (Figure 3, dashed lines) from Equations S3 and S4.

We calculate the yield per unit time from the fit rather than the individual measuring point to reduce the noise amplification associated with differentiation. Our calculation shows that we would be able to produce up to 1.1 g h^−1^ with 2 mm tubing at a flow speed of 6 m min^−1^ and 4.4 g h^−1^ with 3 mm tubing at a flow speed of 17 m min^−1^. This substantial difference is a result of the quadratic dependency of the volume throughput per time (flow rate) on the tubing radius (see Equation S4). The peak yield flow rates are 30.1 mL min^−1^ for 2 mm and 120.4 mL min^−1^ for 3 mm. Hence, a conversion of our solar flow reactor from a circulating design into a fixed length tubing reactor, could lead to a production of 18 times more particles compared to our previous laboratory setup, where we were able to produce 0.24 g h^−1^ of polymeric nanoparticles at a flow rate of 0.8 mL min^−1^ with an inner tubing diameter of 1.3 mm (4.4 g h^−1^/0.24 g h^−1^ = 18.33) [9]. However, for a thorough assessment of the production capacity of our system and how our experiments under the winter and spring sun in Brisbane, Australia translate to other regions worldwide, we investigated the particle growth kinetics during the photopolymerization in our solar flow reactor.

### 2.3 The Two‐Phase Growth Kinetic Determines the Optimal Exposure Time for the Particle Synthesis

**FIGURE 5:**
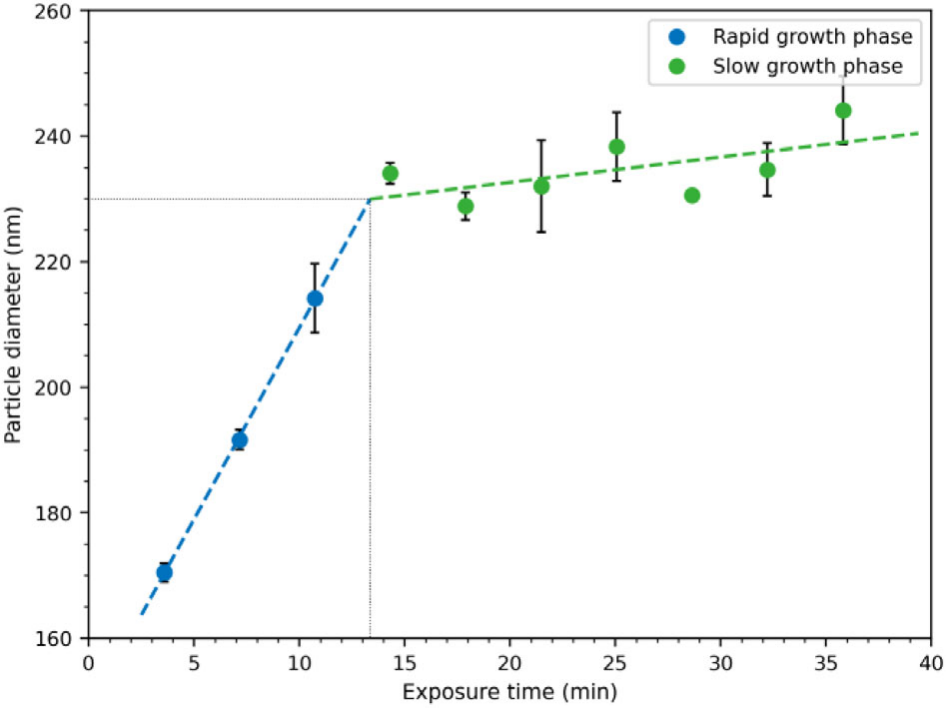
The nanoparticles grow in a rapid (blue) and a slow (green) phase. The measured particle diameter corresponds to the *z*‐average determined by dynamic light scattering (DLS), with the standard deviation from three measurements as the error. The transition from the rapid to the slow phase is determined by the intersection of the linear fits of each phase (dashed lines), allowing the determination of the particle size and exposure time at completion (dotted black lines). Recorded with 2 mm tubing at a flow speed of 15.07 m min^−1^ (experiment 83l, refer to the Section S5 for all experiments and DLS plots).

For the kinetic investigation of the particle growth, we took small samples from the circulating reaction solution to determine the particle diameter by dynamic light scattering (DLS) at given time points. An exemplary growth curve is depicted in Figure 5. Using DLS, only particle sizes >150 nm could be detected reliably, due to the low signal‐to‐noise ratio for smaller particles, which was usually reached after 4–7 min exposure time. From 150 nm on, the particles grow in two distinct phases. A first phase of rapid growth and a second phase of slower growth. The cause of this two‐phase growth requires thorough assessment. A simple concentration effect, for example, the consumption of all monomers slowing the polymerisation reaction is unlikely, as this would lead to a more gradual transition between the rapid and slow phase. The observed transition on the other hand is very rapid. Furthermore, the SEC traces of the filtered reaction solution after the experiment show a large monomer content (Figure S57). These SEC chromatograms further demonstrate that oligomers with more than five incorporated repetition units (>0.7 kDa) begin to precipitate at close to 3 kDa as the absolute solubility limit. Interestingly, these SEC chromatograms also show the absence of oligomer peaks with an even number of incorporated monomer units (AABB, AABBAABB, and AABBAABBAABB), possibly a result of different end group reactivity, or an odd‐even‐effect for the solubility of the oligomers [56].

However, the aforementioned complex interplay of nucleation and growth, tubing diameter, light penetration depths, and flow speed make the root‐cause of the two‐phase particle growth difficult to determine. Especially, the non‐linear kinetics of step‐growth polymerisations and altered light scattering due to the changing particle size make the various experimental influences difficult to untangle. In the present study, we focused on the large‐scale synthetic aspect and the introduction of the global solar photochemical potential. Therefore, the two‐phase characteristic of the particle growth provides two important aspects. First, as the polymerisation continues in the second growth phase, the remaining reaction solution must stay photoreactive, suggesting that the reaction solution could, in theory, be recycled after isolating the solid nanoparticles from it. Second, the transition between the two phases provides a well‐defined completion criteria for the photochemical reaction. Due to the sluggish growth in the second phase, the reaction should not be continued once the rapid growth phase is completed to maximise efficiency. We determined this point of completion as the crossover of two linear fits applied to each of the two phases. The average size at completion from DLS is slightly smaller for the 2 mm tubing with 206 nm ± 13 nm, compared to 225 nm ± 16 nm with 3 mm tubing (standard deviation as error). Based on the determined sizes at completion of the individual flow speeds (Sections S5.1 and S5.2), we cannot identify any influence of the flow speed on the particle size.

Compared to SEM, which visualises the dried particles, DLS gives larger values for size as a measure of the swollen hydrodynamic diameter (compare Sections S5.2 and S5.3). In addition, the DLS data represent the *z*‐average size, while the SEM data represent the number average size. However, more importantly than determining the exact particle size at reaction completion, DLS analysis allowed us to simultaneously determine the exposure time at completion. The exposure times to completion are similar for both tubing diameters with 14.3 min at 2 mm and 15.0 min at 3 mm.

The experiments presented thusfar were conducted in the southern hemisphere in Brisbane, Australia during winter and spring (from June to October—the typically sunnier, drier part of the year in this sub‐tropical climate). To make the exposure time to completion comparable between the different weather conditions and times of the year during the experiments, we normalised it by multiplying it with the average UV index during the initial rapid growth rate, affording a normalized exposure time to completion of 43 min ± 19 min at 2 mm and 78 min ± 27 min at 3 mm, with the standard deviation as error.

We obtained the local UV index for Brisbane as minute wise measurements from the Australian Radiation Protection and Nuclear Safety Agency (ARPANSA) [57]. The UV index is a linear measure for the intensity of the UV radiation weighted by its biological activity, either given in W m^−2^∙40 or dimensionless [58]. Its weighting does not exactly correspond to the wavelength‐resolved reactivity of our system, but since the photocycloaddition is governed by UV light below 380 nm, we deem the UV index—a sufficiently accurate measure of the relevant radiation intensity. Furthermore, after the normalisation, the exposure time to completion shows the expected trend of a longer necessary irradiation time for the 3 mm tubing compared to the 2 mm tubing. This expected relation is not visible in the non‐normalised data as we conducted most of the 2 mm experiments in winter, when the UV intensity is generally lower.

### 2.4 Geographical Validation

Before we progress to predictive modelling of particles on a global scale, it was critical to assess the performance of our reactor system at a different geographical location. We selected Karlsruhe, Germany, as a northern hemisphere antipodean location to Brisbane, going from a subtropical to a temperate climate zone. We constructed an entirely identical reactor set‐up and conducted the sun flow experiments in early October (refer to the Sections S1 and S2 for the details of the German‐based sun flow reactor and the associated data of the sun flow experiments conducted in Germany). The construction of the reactor used locally sourced parts and the construction proceeded fully independently of the Brisbane installation, merely following its design. The independence of the installation was intentional and evidences that the reactor yields data that is fully independent of location and operator. Pleasingly, our validation study yielded highly comparable results to those obtained in Brisbane, Australia, in terms of particle yield, size, and dispersity for the assessed flow speed of 17 m min^−1^ (refer to Figures 3 and S20).

Subsequently, using the UV index as a measure for the UV intensity, we were able to hypothetically translate our local experimental results to any region in the world.

### 2.5 Nanoparticle Synthesis for the Real World

**FIGURE 6:**
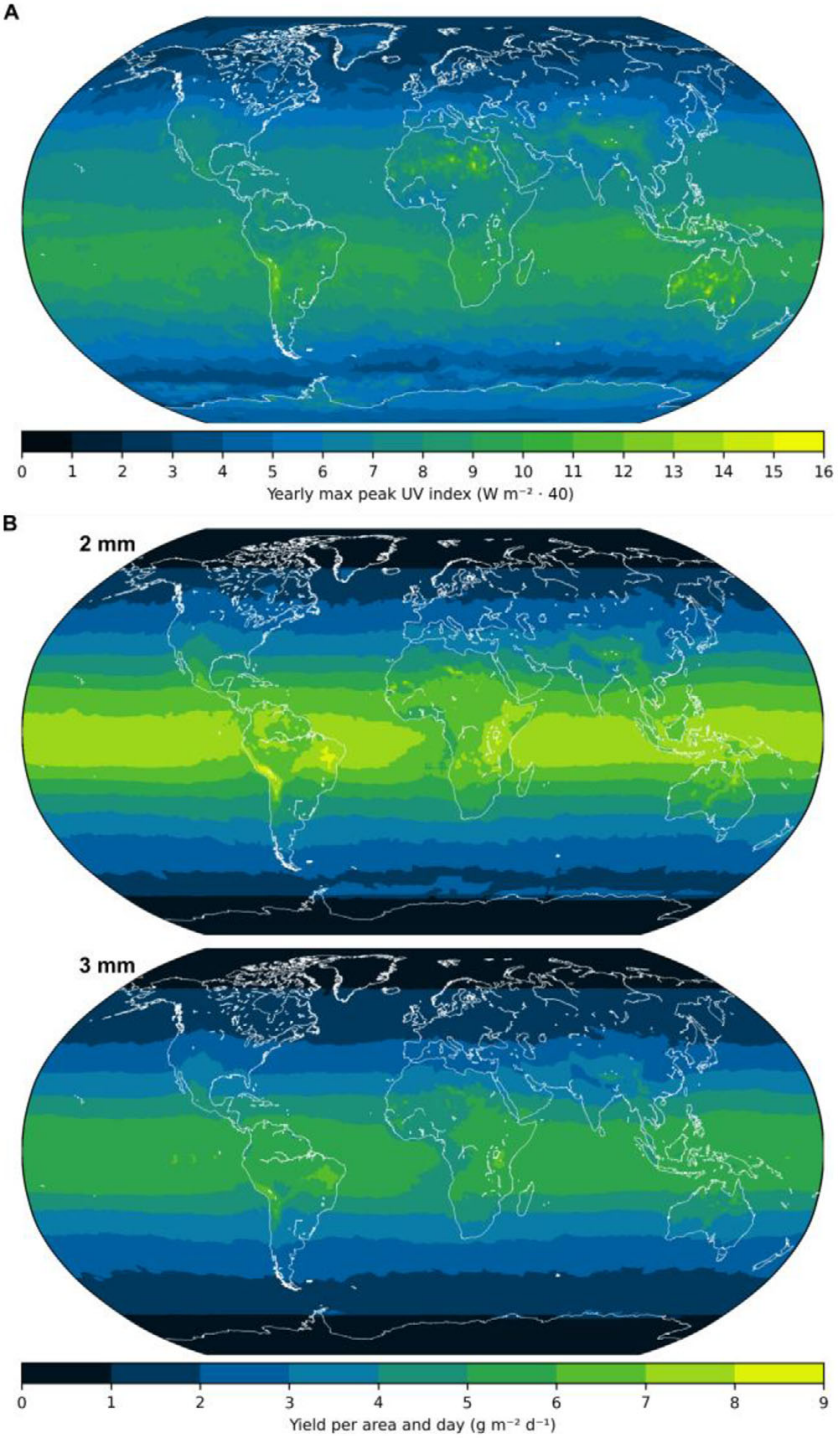
Extrapolation of our experimental data to global solar potential expressed by the yield per area and day. (A) Yearly maximum of the peak UV index to illustrate the all‐sky UV irradiance in different regions of the world [59]. (B) Global particle yields per day and square meter of a hypothetical chemical plant, that uses our chemical system and reactor design with 2 or 3 mm tubing.

Using the UV index for normalisation of the kinetic data allows us to translate our experimental results to the particle yield of a hypothetical chemical plant that uses our solar reactor design anywhere in the world. Therefore, we used the all‐sky UV index data of 2023 of the National Aeronautics and Space Administration's (NASA) *The Power Project* [59], which accounts for the local average cloud cover (refer to Figure 6A and Section S8.2). The relevant figure when translating our reactor design into a hypothetic solar photochemical plant is the yield per day and area

(1) $$Y=\frac{\dot{y}\cdot t_{\mathrm{reac}}}{A}=\frac{\pi }{1.1\cdot 2.5}\cdot \frac{y\cdot r^{2}\cdot c\cdot t_{\mathrm{reac}}\left(t_{0}\right)\cdot \mathrm{UVI}\left(t_{0}\right)}{T\cdot w}$$

where $\dot{y}$ is the yield per unit time, *t*_reac_ the runtime of the reaction per day, *A* is the area required for the plant, *y* is the relative yield, *r* is the inner tubing radius, *c* is the concentration of the reaction solution, UVI(*t*_0_) is the UV index at reaction start, *t*_0_ is the start time of the reaction, *T* is the UV index normalised time to completion, and *w* is the lateral space required by the tubing, for which we used a center‐to‐center distance of the adjacent tubes of 1 cm. A more detailed derivation of Equation 1 is provided in Section S7. The most important assumptions are summarised below.

From a construction point of view, the flow reactors of the solar chemical plant must be built with a fixed tubing length. This length is determined by the flow speed of the reaction solution and the time to completion, so that the reaction is completed when the solution has passed through the tubing (refer Equation S8). Thereby, the time to completion is a function of the UV index (refer to Equation S9). To achieve the highest possible yield, the function in Equation 1 must be optimised for the ideal UV index at reaction start UVI(*t*_0_). The total reaction time *t*_reac_(*t*_0_) is thereby linked to the UV index, being the time until the UV index decreases below UVI(*t*_0_) again that is until the tubing length does not suffice anymore to complete the reaction at the selected flow speed. Additionally, we introduced a buffer of an extra 10% tubing length, which leads to the factor of 1.1 in Equation 1. The additional factor of 2.5 accounts for the total space requirement of the solar plant, for example, for supporting structures and to avoid shadowing effects. The value of 2.5 is based on the typical space requirement for solar power plants, where an area of two to three times the area of the solar modules is needed [60].

As the daylight hours and peak UV index are seasonal, we performed the optimisation of Equation 1 with respect to UVI(*t*_0_) based on monthly UV index and daylight hour data (Sections S8.1 and S8.2). Before discussing the results of this optimisation, we note that the yield per day and area from Equation 1 does not depend on the flow speed anymore, since with increasing flow speed, the tubing length must increase, and thus the required area of the chemical plant. This contrasts with what we observed for our individual solar flow reactor, which achieves the highest particle yields per time at relatively high flow speeds (compare Figure 3, dotted line). This is due to the fact that for an individual single laboratory scale setup the area requirement is generally lower and less of a concern compared to a large‐scale chemical plant; that is, for an individual solar flow reactor, the highest particle yield per unit time is achieved for higher flow speeds, where the high volume throughput outweighs the lower relative yield. The large scale solar chemical plant on the other hand, should be operated at the flow speed where the relative yield is maximal. Thus, we used for the calculation of the yield per area and day of a solar plant with Equation 1 the maximal experimentally obtained yields of 66% for 2 mm and 41% for 3 mm.

On a global scale, the highest potential particle yields per unit time and area can be achieved in the Andes, Brazil, and Uganda with up to 8.7 g m^−2^ d^−1^ for 2 mm diameter tubing and 6.7 g m^−2^ d^−1^ for 3 mm diameter tubing (refer to the yield maps in Figure 6B). Despite the quadratic dependency of the yield per area and day on the tubing radius (refer to Equation 1), the yield with the smaller 2 mm tubing is higher, due to the larger relative yield, and the shorter time to completion.

With a yield of 8.7 g d^−1^ m^−2^ and 2 mm tubing, a hypothetical solar plant the size of a standard soccer field of 105 m by 68 m could produce a total of about 62 kg of nanoparticles per day and 23 t per year. In most other equatorial regions (Northern Australia, Mexico, and some North African regions, for example), about 50 kg d^−1^ (18 t y^−1^) could be produced. Even in less sunny regions such as Northern Europe, a nanoparticle production of 7 kg d^−1^ (3 t y^−1^) is possible, even though a peak UV index of only 3 W m^−2^∙40 (yearly maximum) is reached (see Figure 6A). It is noted that the herein described particle yields are the yearly average and in summer much higher yields can be obtained (refer to monthly resolved data in Section S8.4).

To place the calculated yields into perspective, we note that our calculation is rather conservative by including a safety margin of 10% into the calculation. Additionally, our reactor is of the simplest design, built with readily available materials. A more sophisticated design [2], for example, with variable flow speed to utilise the varying UV index and reaction speed during the day, and optimised reactor materials would greatly enhance the yield. Especially improved surface engineering of the reactor tubing to prevent polymer deposition could improve the reported particle yields by 40% (based on 92% yield for the first run with new 2 mm tubing and maximum 66% for subsequent runs). Thus, we see immense potential to produce polymeric nanoparticles with our system on a large scale by directly harvesting the synthetic power of the sun.

To place the potential particle yields further into perspective, for example, the 50 kg d^−1^ produced by a plant the size of a soccer field in Northern Australia, we point out that a soccer field is comparably small for a solar farm and the particle production can be linearly upscaled by increasing the plant area. Furthermore, the product is polymeric nanoparticles, which are otherwise produced by complex processes and in small quantities [24, 25, 26, 28, 29, 30, 31, 32, 33, 34, 35]. In contrast, the particle precipitate in our system from a homogeneous medium and require no additives. At the same time, they are of high uniformity and stability (refer to Section S13), insoluble and their size can be adjusted by the choice of solvent system [9].

## 3 Conclusion

We introduce the synthesis of polymeric nanoparticles by precipitation polymerisation via the Diels–Alder photocycloaddition of a bismaleimide and a difunctional *o*‐methylbenzaldehyde in a custom‐built solar flow reactor. We show that the particles are of high quality (low dispersity and high roundness) and reveal the interplay between the tubing diameter and flow speed of the reaction solution. The underlying mechanism is based on the penetration depth of the solar radiation into the reaction solution, resulting in concentration and molecular weight gradients of the generated oligomers, which leads to wider tubing and higher flow rates to more oligomer deposition onto the walls of the tubing, affecting the nucleation and growth of the particles.

Furthermore, we investigated the particle growth kinetics as we designed our reactor to enable the extraction of samples during the solar synthesis. The particles grow in a two‐phase process with an initial rapid, and a subsequent slower phase. We defined the transition between the first phase and second phase as the completion of reaction, as the reaction precedes inefficiently afterwards. Taking the UV index as a measure for UV radiance—which governs the photopolymerisation—we can extrapolate from our locally determined time to completion of the reaction to a potential particle yield anywhere in the world.

As noted above, we conducted sun‐flow experiments not only in Australia, but also in Germany, demonstrating that these results are congruent. Thereby, our calculations show that our system is suitable for the year‐round large‐scale solar flow synthesis of polymeric nanoparticles as we calculate that a soccer field‐sized chemical plant could easily produce several tons a year at various places in the world. Our results suggest that the yield could still be dramatically enhanced by optimising the reactor design and eliminating yield losses with suitable surface engineering of the tubing.

We hope that our results encourage other researchers in the field of photochemistry to explore the sun as a most sustainable driver of photochemical reactions. In fact, there are various promising synthetic results and reactor designs for solar photochemistry reported [47, 48, 49, 50]. However, despite the large volume of work to build upon, there is still a long way to go to realise and broadly implement solar photochemical syntheses on a large scale. The relevant literature is dominated by locally recorded yields, obtained under not entirely controlable conditions [61]. Herein, we attempt to make solar photochemistry and our reactor design internationally comparable by calculating the global solar photochemical potential expressed as the yield per area and day. Our simple approach was inspired by the Global Solar Atlas [62] and developed for a UV light‐driven synthesis. To generalise this approach for other non‐UV light‐driven systems, it needs to be adapted as follows: (i) The active wavelength window that drives the reaction must be determined, most accurately by action plots, which provide the wavelength‐resolved reactivity (refer to Figure 2A). (ii) The irradiance of the active wavelengths needs to be recorded during the local experiment. (iii) The data can subsequently be used to calculate the global solar photochemical potential by modifying Equation 1 for the specific photochemical system. Thereby, it is especially important to use the all‐sky irradiance of the active wavelengths, which takes cloud cover into account, as longer wavelengths are stronger scattered by clouds than UV light [63].

We submit that making the solar performance of photochemical systems globally comparable will further propel and emphasise the key potential of sun‐induced chemistry, bringing us one step closer to Ciamician's vision of a more sustainable future.

## Conflicts of Interest

The authors declare no conflicts of interest.

## Supporting information




**Supporting Information file 1**: The authors have cited additional references within the Supporting Information [2–4, 10]. The Supporting Information contains the experimental details and additional data supporting the study as well as the detailed derivation of Equation 1. Furthermore, the script to calculate the yield per time and area is provided as the jupyter notebook *SI_global_solar_photochemical_potential.ipynb* with the Anaconda environment *SI_worldmaps.txt* to execute it.

**Supporting Information file 2**: anie71361‐sup‐0002‐Data.zip.

## Data Availability

The data that support the findings of this study are available in the Supporting Information of this article.
