# Supplementary material for: Solar Flow Synthesis of Polymer Nanoparticles: Scaling Local Experiments to Global Potential
Source: Angew Chem Int Ed Engl. 2026 Feb 3;65(11):e24746. doi: 10.1002/anie.202524746 (PMC12970522; doi:10.1002/anie.202524746)
Supplement: Supplementary file 1 — Supporting Information file 1: The authors have cited additional references within the Supporting Information [2–4, 10]. The Supporting Information contains the experimental details and additional data supporting the study as well as the detailed derivation of Equation 1. Furthermore, the script to calculate the yield per time and area is provided as the jupyter notebook SI_global_solar_photochemical_potential.ipynb with the Anaconda environment SI_worldmaps.txt to execute it. [file ANIE-65-e24746-s002.pdf]

Solar Flow Synthesis of Polymer Nanoparticles: Scaling Local Experiments to Global Potential

Jochen A. Kammerer,[a,b]+ Joshua O. Holloway,[a,b]+ Theresa Stephan,[d]+ Hartmut Gliemann,[d] Florian Feist,[d] Fred Pashley-Johnson,*[a,b,c]# Laura Delafresnaye*[a,b] and Christopher Barner-Kowollik*[a,b,d]

[a] Dr Jochen A. Kammerer, Dr Joshua O. Holloway, Fred Pashley-Johnson, Dr Laura Delafresnaye and Prof. Christopher Barner-Kowollik
School of Chemistry and Physics
Queensland University of Technology (QUT)
2 George Street, Brisbane, 4000 QLD, Australia
E-mail: christopher.barnerkowollik@qut.edu.au; laura.delafresnaye@qut.edu.au; fred.pashley-johnson@dpag.ox.ac.uk

[b] Dr Jochen A. Kammerer, Dr Joshua O. Holloway, Fred Pashley-Johnson, Dr Laura Delafresnaye and Prof. Christopher Barner-Kowollik
Centre for Materials Science
Queensland University of Technology (QUT)
2 George Street, Brisbane, 4000 QLD, Australia
E-mail: christopher.barnerkowollik@qut.edu.au

[c] Fred Pashley-Johnson
Polymer Chemistry Research Group, Centre of Macromolecular Chemistry (CMaC), Department of Organic and Macromolecular Chemistry, Faculty of Sciences, Ghent University
Krijgslaan 281-S4bis, Ghent, 9000, Belgium

[d] Prof. Christopher Barner-Kowollik, Theresa Stephan, Dr. Hartmut Gliemann, Dr. Florian Feist
Institute of Functional Interfaces (IFG), Karlsruhe Institute of Technology (KIT), Hermann-von-Helmholtz-Platz 1, 76344 Eggenstein-Leopoldshafen, Germany
E-mail: christopher.barner-kowollik@kit.edu

+ Authors contributed equally.

# current address: Department of Physiology, Anatomy and Genetics, University of Oxford, OX1 3QU Oxford, U.K.

Table of Contents

1. Materials 3
2. Methods 5
3. Monomer Synthesis 7
3.1. Synthesis of 4-hydroxy-5-isopropyl-2-methylisophthalaldehyde 7
3.2. Synthesis of AA Monomer - 5-isopropyl-4-(2-(2-(2-methoxyethoxy)ethoxy) ethoxy)-2-methylisophthalaldehyde 7
4. Particle synthesis using the solar flow reactor and related sample extraction 7
4.1. Particle synthesis using the solar flow reactor 7
4.2. DLS sample extraction and preparation 8
4.3. Particle isolation and work up 8
4.4. SEC sample preparation 8
5. Experimental results 8
5.1. Experiment overview 8
5.2. Determination of time to completion by DLS 10
5.2.1. 2 mm diameter tubing 10
5.2.2. 3 mm diameter tubing 16

5.3. Size and dispersity analysis by SEM 19
5.3.1. Segmented SEM images: 2 mm diameter tubing 22
5.3.2. Segmented SEM images: 3 mm diameter tubing 30
5.3.3. Histograms: 2 mm diameter tubing 39
5.3.4. Histograms: 3 mm diameter tubing 44
5.4. SEC analysis 48
6. Relative yield and yield per time calculation 49
7. Derivation of the formula for yield per area and day of a solar chemical plant 49
8. Additional calculations and global data plots 51
8.1. Monthly average daylight hours per day 51
8.2. Monthly average peak UVI-index 58
8.3. Optimal UV index at reaction start 64
8.4. Monthly resolved yield per area and day 64
8.4.1. 2 mm diameter tubing 64
8.4.2. 3 mm diameter tubing 71
9. Fourier-Transform Infrared Spectroscopy of Nanoparticles 77
10. NMR and DLS from irradiation with a 365 nm LED 78
11. Reynolds Number 81
12. Residence time distribution determined using UV/Vis spectroscopy 81
13. Particle stability 84
14. Reactor fouling 85
15. References 86

1. Materials

All chemicals were reagent grade and used as received, unless stated otherwise: 2-isopropyl-5-methylphenol (≥ 98.5 %, Sigma-Aldrich), hexamethylenetetramine (≥ 99.5 %, Sigma-Aldrich), sulfuric acid (96%, Thermo-Fischer), hydrochloric acid (32%, Thermo-Fisher) 2-[2-(2-methoxyethoxy)ethoxy]ethyl bromide (97%, Combi-blocks), acetic acid (glacial) (Thermo-Fisher), oxalyl chloride (≥99%, Sigma-Aldrich) potassium carbonate (99.9 %, Merck), triethylamine (Sigma-Aldrich, ≥99.5%), N,N ′-(1,3-Phenylene)dimaleimide (97%, Sigma-Aldrich), 4-Amino-2,6-dichlorophenol (98%, Combi-blocks), maleic anhydride (99%, Sigma-Aldrich), N,N'-dimethylformamide, anhydrous (DMF, 99.8% Sigma-Aldrich) ethyl acetate, anhydrous (Sigma-Aldrich, 99.8%) N,N'-dicylohexylcarbodiimide (DCC, Sigma-Aldrich), magnesium sulphate anhydrous (Merck), acetonitrile (ACN, HPLC-grade, Thermo-Fisher), dimethyl sulfoxide (DMSO, analytical reagent, Thermo-Fisher), acetone (analytical reagent, Thermo-Fisher) methanol (MeOH, analytical reagent, Ajax Finechem), tetrahydrofuran (THF, 99.8% analytical reagent, Fisher), cyclohexane (CH, analytical reagent, Ajax Finechem), ethyl acetate (EA, analytical reagent, Thermo-Fisher), dichloromethane (DCM, analytical reagent, Thermo-Fisher), n-pentane (analytical reagent, Thermo-Fisher) acetonitrile-d_3 (ACN-d_3, 99.8 %D, Cambridge Isotope Laboratories), chloroform-d ($CDCl_3$, 99.8 %D, Cambridge Isotope Laboratories), dimethylsulfoxide-d_6 (DMSO-d_6, 99.9 %D, Cambridge Isotope Laboratories), tetrahydrofuran-d_8 (THF-d_8, 99.5 %D, Cambridge Isotope Laboratories).

Sun Flow Reactor in Brisbane (Australia)

The construction materials for the solar flow reactor were purchased from the Australian hardware store *Bunnings Warehouse*, including the 300 mm by 300 mm acrylic mirrors from the brand *Stylit*.

The solar reactor was constructed with a wooden structure (Figure S1A). The front face of the reactor was covered with the acrylic mirrors and oriented towards the azimuth of the sun in zenith with an inclination of 45° (Figure S1B). The tubing was attached vertically on the ca. 0.8 m high front face, spanning the face six times for the 2 mm diameter tubing, four times for the 3 mm diameter tubing, and twice for the 5 mm diameter tubing. The tubing was laid approximately 2 cm in front of the mirrors (Figure S1C).

To choose the best possible tubing for the reactor, several specimens were acquired and tested for their transmittance in the relevant wavelength regime of 310 nm to 380 nm (Figure S1D), since it was known that the transmittance of PTFE can vary depending on manufacturer due to the different additives used during production. The PTFE tubing was purchased online from Ebay and had a wall thickness of 1 mm. Of these samples, PTFE Sample 2 was least absorbing and selected for the construction of the reactor (see Figure S1). Other tubing was kindly provided by *Vapourtec* (samples PTFE Vaportec PTFE and PFA Vapourtec), and was analysed, along with PVC tubing, purchased from Clark Rubber Franchising Pty Ltd. It was found that the tubing samples from *Vapourtec* and PVC had poor transmittance (see Figure S1)

All tubing, that was not directly located above the acrylic mirrors, was covered with cloth tape to ensure consistent irradiation profiles. The reactor was constructed in a circulating design using a peristaltic pump (MasterFlex L/S, supplied by John Morris) with silicone tubing. Before the peristaltic pump, a 30 mL centrifuge tube was installed into the circuit as reservoir for easy sample extraction. The reservoir was filled with about 2 mL of reaction solution during the experiments. Reservoir and pump were enclosed in an insulated box to avoid unwanted sun exposure, adverse temperature under the midday sun and contamination.

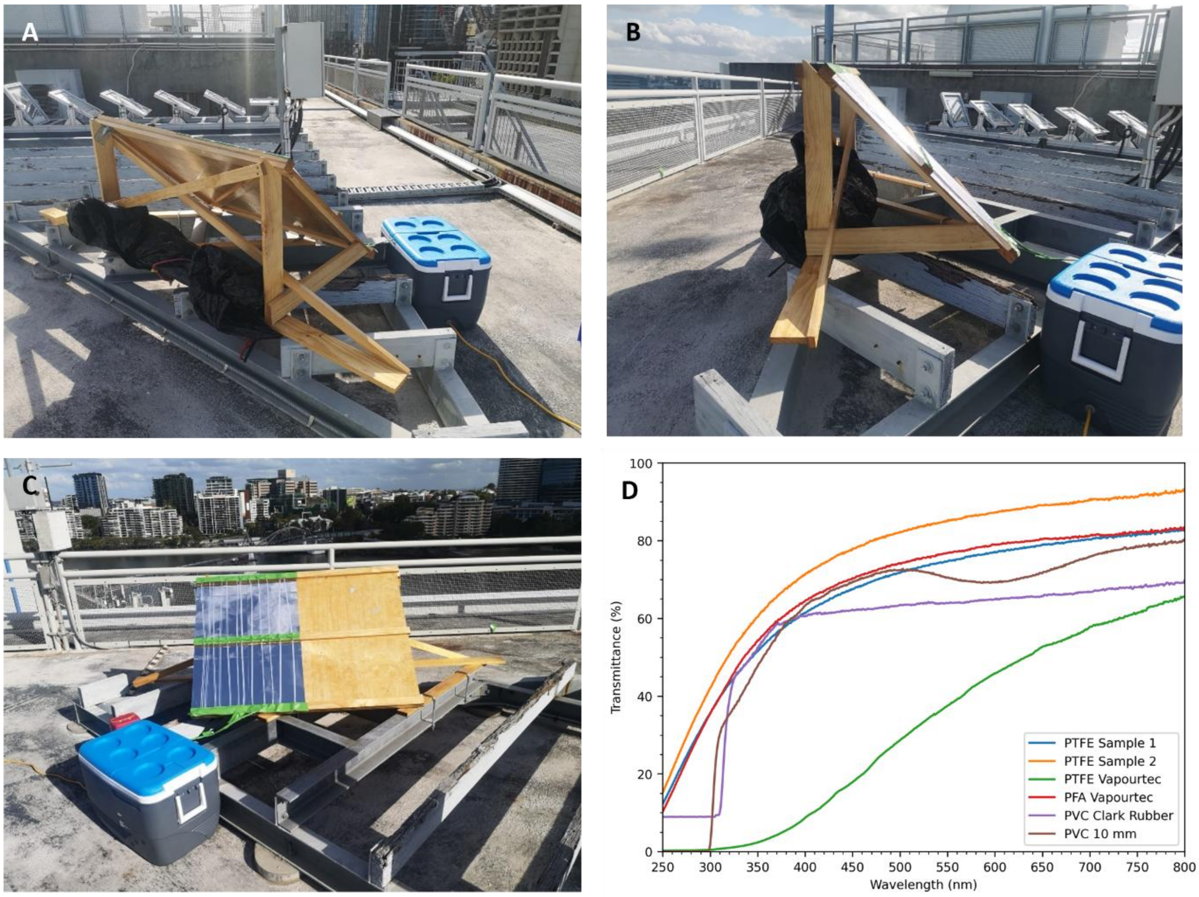

Figure S1. Construction of the solar flow reactor in Brisbane (Australia). **A**. Wooden structure. **B**. Orientation of the mirror and tubing towards the azimuth of the sun in zenith with an inclination of 45°. **C**. Overview of the set-up (reservoir and peristaltic pump were enclosed in an insulated box to avoid unwanted sun exposure and contamination). **D**. Transmittance of various tubing samples, of which PTFE Sample 2 was selected for the solar flow reactor.

Sun Flow Reactor in Karlsruhe (Germany)

The reactor for the experiments that were carried out in Karlsruhe (Germany) (Figure S2) was constructed in accordance with the reactor in Brisbane (Australia). A structure made of 4.5 cm aluminium profiles was used as the stand. The mirror consists of two acrylic mirrors, each measuring 50 cm × 70 cm with a thickness of 3 mm, supplied by the company Firstlaser GmbH (Germany). The mirror plates as well as the tubing was mounted on an OSP board from Hornbach (Germany). The wooden board was installed at an inclination angle of 45°.

The exact same PTFE tubing (3 mm inner diameter) was used, together with a Hei-FLOW Ultimate peristaltic pump with silicone tubing. All tubing that was not directly located above the acrylic mirrors was covered with aluminum foil. Before the peristaltic pump, a 45 mL centrifuge tube reservoir was installed into the circuit for sample extraction. At the start of the experiment, the reservoir was filled with 22 mL of reaction solution, which was reduced to 2 mL within the first 17 min of exposure time as 10 x 2 mL samples were extracted.

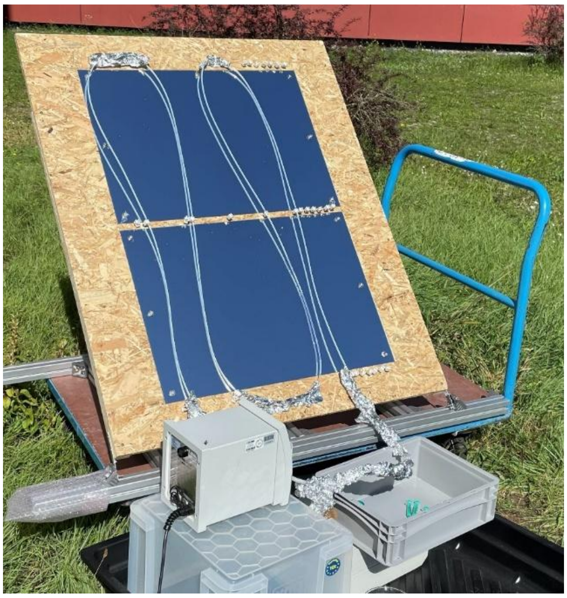

Figure S2. Construction of the solar flow reactor for the experiments in Karlsruhe (Germany), which follows the construction parameters of the solar flow reactor set-up in Brisbane (Australia).

2. Methods

Experiments were conducted in two locations, one in the southern hemisphere (Brisbane, Australia) and one in the northern hemisphere (Karlsruhe, Germany). All analytical equipment used in Karlsruhe is described separately. If no further information is provided, the analysis methodology was identical to that conducted on the data obtained from the equipment in Brisbane.

Scanning Electron Microscopy (SEM): SEM images were acquired using a Tescan MIRA3 using an in-lens, in-beam secondary electron (SE) or the beam deacceleration detector at a 1.8 mm to 4 mm working distance and 0.5 kV to 2 kV acceleration voltage. Samples were prepared by dispersing the particles in methanol and drop casting onto a silicon wafer glued onto a SEM stub using conductive carbon tape. Samples were then coated with a 5 nm platinum layer. For the quantitative analysis regarding size and dispersity, the size of the particles was analysed with an automated script[1] based on an edge detection and the *UCB Vison Science*'s "Hough Circle Transform" plugin for Fiji[2] (ImageJ)[3]. We calculated the dispersity as[4]

$$Đ = \left(\frac{\sigma}{\bar{d}}\right)^2 \quad \text{(S1)}$$

with $\bar{d}$ as the mean particle diameter and σ as its standard deviation. The complete size analyses with processed and segmented images, size distributions, and dispersities are displayed in Section S5.3.

Scanning Electron Microscopy (SEM), Karlsruhe: A Cressington Sputter Coater 108 auto was used to coat the 3D-printed microstructures with a 10 nm layer of gold prior to scanning electron microscopy (SEM) analyses. The SEM images were recorded using a Zeiss Leo 1530 operating at 10.0 kV electron energy.

Nuclear Magnetic Resonance (NMR) Spectroscopy: ^{1}H and ^{13}C-NMR spectra were recorded on a *Bruker* System 600 Ascend LH, equipped with a BBO-Probe (5 mm) with z-gradient (^{1}H: 600.13 MHz, ^{13}C 150.90 MHz) or on a Bruker AM 400 equipped with a PABBO-Probe (5 mm) (^{1}H: 400 MHz, ^{13}C: 101 MHz). The δ-scale was normalised relative to the residual solvent resonance of $CDCl_3$, DMSO-d_6, acetonitrile-d_3 or tetrahydrofuran-d_8 for ^{1}H spectra and ^{13}C spectra on the middle signal of $CDCl_3$ triplet, the DMSO-d_6 quintet, the acetonitrile-d_3 quintet or the tetrahydrofuran-d_8 singlet. The multiplicities were

reported using the following abbreviations: s = singlet, d = doublet, t = triplet, q = quartet, quin = quintet, m = multiplet and br = broad signal.

Nuclear Magnetic Resonance (NMR) Spectroscopy, Karlsruhe: ^{1}H NMR spectra were recorded on a Bruker AM 400, equipped with a 5 mm BBO-Probe (^{1}H: 400 MHz). The δ-scale was normalised relative to the residual solvent resonance of acetone-d6 (δ = 2.05 ppm).

Flash Chromatography: Flash chromatography was performed on a Interchim XS420+ flash chromatography system consisting of a SP-in-line filter 20-µm, an UV-VIS detector (200-800 nm). The separations were performed using an Interchim dry load column and a Interchim Puriflash Silica HP 30 µm column after deposition on Celite® 565 (Sigma-Aldrich).

Centrifuge: The particles were isolated by centrifugation using a Sigma 3-16 L centrifuge, as specified in the experimental details in the Section S4.3.

Size Exclusion Chromatography (SEC): The SEC measurements were conducted on a PSS SECurity system consisting of a PSS SECurity Degasser, PSS SECurity TCC6000 Column Oven (35 °C), PSS SDV Column Set (8x150 mm 5 µm Precolumn, 8x300 mm 5 µm Analytical Columns, 100000 Å, 1000 Å and 100 Å) and an Agilent 1260 Infinity Isocratic Pump, Agilent 1260 Infinity Standard Autosampler, Agilent 1260 Infinity Diode Array and Multiple Wavelength Detector (A: 254 nm, B: 360 nm), Agilent 1260 Infinity Refractive Index Detector (35 °C). HPLC grade THF, stabilized with BHT, is used as eluent at a flow rate of 1 mL·min^{-1}. Narrow disperse linear poly(styrene) (M_n: 266 g·mol^{-1} to 2.52x106 g·mol^{-1}) and poly(methyl methacrylate) (M_n: 202 g·mol^{-1} to 2.2x106 g·mol^{-1}) standards (PSS ReadyCal) were used as calibrants. All samples were dissolved to the concentration of 1mg mL^{-1} and subsequently filtered through a 0.22 µm PTFE membrane. Molecular weight and dispersity analysis was performed in PSS WinGPC UniChrom software (version 8.2).

Dynamic Light Scattering (DLS): The z-averaged size of the nanoparticles was measured on a Malvern Zetasizer NanoZS with the Malvern Zetasizer software version 7.13. For each sample, three individual measurements, consisting of three 10 s measurement runs each, were acquired and averaged. The measurements were done in water at 20 °C.

Dynamic Light Scattering (DLS), Karlsruhe: The z-averaged size of the nanoparticles was measured on a Malvern Zetasizer Nano System ZEN 3600. The same conditions and settings were used as in the Brisbane experiments.

UV-vis spectroscopy: UV-vis spectra were acquired on a Shimadzu UV-2700 spectrometer at room temperature with quartz cuvettes. For the measurement of the absorbance of the reaction solution, a concentration of 0.001 mmol L^{-1} was used. For the measurement of the transmittance of the tubing, the tubing was cut open longitudinally and flattened under a weight for a day. The flattened tubing was then measured in a quartz cavity of about 1 mm thickness.

3. Monomer Synthesis

All of the following compounds were synthesised and characterised in accordance with previously published work.[5] Purity was assessed via ^{1}H NMR spectroscopy and matched literature values.

3.1. Synthesis of 4-hydroxy-5-isopropyl-2-methylisophthalaldehyde

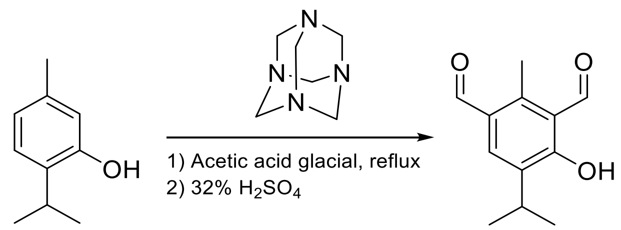

Scheme S1. Synthesis of 4-hydroxy-5-isopropyl-2-methylisophthalaldehyde

3.2. Synthesis of AA Monomer - 5-isopropyl-4-(2-(2-(2-methoxyethoxy)ethoxy) ethoxy)-2-methylisophthalaldehyde

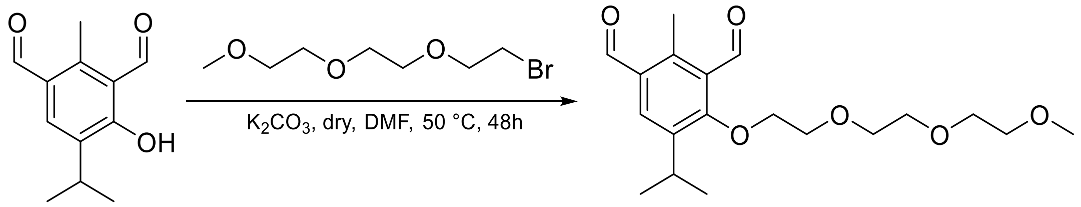

Scheme S2. Synthesis of 5-isopropyl-4-(2-(2-(2-methoxyethoxy)ethoxy) ethoxy)-2-methylisophthal-aldehyde (AA)

4. Particle synthesis using the solar flow reactor and related sample extraction

4.1. Particle synthesis using the solar flow reactor

A 10 mmol L^{-1} equimolar stock solution of the AA and BB monomer was prepared in a 1:1 mixture of water and acetone (the monomers were first dissolved in acetone before adding the Milli-Q water). The solution was subsequently passed through a 0.2 µm PTFE syringe filter and then bubbled with argon for 5 minutes. The solar flow reactor tubing was flushed with dichloromethane, followed by acetone. Subsequently, the stock solution was sent through the solar flow reactor at a predetermined flow speed (refer to the main article), using an rpm controlled peristaltic pump. The reaction solution was then continuously recirculated for one to two hours and collected afterwards (refer to Section S5.1 for details). The sun exposure time was calculated by multiplying the exposure time with the ratio of the time necessary to pass the exposed tubing length and the time of a full pass.

Before and after each run, the tubing was purged with dichloromethane followed by acetone to clean it. We note that this cleaning procedure does not dissolve the polymer deposit on the tubing wall. However, we still performed the cleaning procedure before and after each experiment for consistency and comparability with previous experiments. The cleaning procedure occasionally led to the removal of the snakeskin-like polymer film deposit from the tubing wall by swelling and shrinkage of the film, resulting in its detachment. A mechanical cleaning step by flushing cotton balls or yarn pieces through the tubing was later identified as more efficient. This procedure was applied when a large amount of polymer deposit built-up on the 3 mm tubing wall, as the detachment by swelling and shrinkage was not efficient for the larger inner diameter.

4.2. DLS sample extraction and preparation

During circulation, 10 µL of reaction solution was periodically taken from the sample reservoir of the solar flow reactor and added to 1.3 mL of water. The time interval between the sample extractions, which were used for DLS analysis, was the time necessary for a full pass of the reaction solution through the reactor, or an integer multiple thereof. To avoid measuring mixed samples with different exposure times, the samples were always taken in the middle of a pass.

4.3. Particle isolation and work up

After the particle synthesis, the resulting turbid solution was collected and centrifuged at 9000 rpm for 5 min (time started when the centrifuge reached 9000 rpm). Subsequently, the supernatant was decanted off, and the solid particle pellet was washed by redispersing it in 25 mL of THF by shaking or sonicating, as required. The dispersion was then centrifuged again at 9000 rpm for 1 min 30 s and then decanted. The isolated particles were then redispersed in methanol (ca. 5 mL) by shaking and sonication as required. A small quantity of the dispersion (10 µL) was drop cast onto a Si wafer piece for SEM analysis. The rest of the dispersion was transferred into pre-weighed centrifuge tubes and dried at 60 °C in an oven.

4.4. SEC sample preparation

For selected samples, the supernatant of the first centrifugation step was filtered with a 0.22 µm syringe filter and dried under reduced pressure in amber vials. These residues were subsequently analysed by SEC.

5. Experimental results

5.1. Experiment overview

An overview of all experiments and their experimental parameters is given in the tables below (Table S1 and Table S2). The plots to determine the time to completion and size at completion are depicted in Section S5.2. For some samples, no size to completion or related values could be given, as either the time per pass was too high to obtain a useful time resolution, no particle formation occurred (relative yield = 0), or the DLS samples degraded before measurement.

Table S1. Experiment overview for 2 mm diameter tubing.

Experiment	Start	Flow rate (mL min^{-1})	Circulation time (min)	Exposure time (min)	Flow speed (m min^{-1})	Average UV-index[6]	Time to completion (min)	Time to completion UV index normalised	Size at completion (nm)	Relative yield (%)
83a*	04.06.2024 09:37	2.39	110	63	0.75	3.3	23	78	288	92%
83b	04.06.2024 14:00	3.59	92	53	1.13	1.8	13	25	208	57%
83c	05.06.2024 10:50	4.79	73	42	1.51	3.6	11	41	203	49%
83d	05.06.2024 12:19	1.80	80	46	0.57	3.6	21	75	224	66%
83e	05.06.2024 14:02	7.18	63	37	2.26	1.7	9	16	199	51%
83f‡	06.06.2024 11:23	2.39	60	35	0.75	3.7	19	73	195	36%
83g	06.06.2024 12:53	11.97	57	33	3.77	3.1	16	48	209	42%
83l	28.06.2024 11:17	47.89	63	36	15.07	3.6	13	49	230	9%
83m	28.06.2024 12:55	95.78	106	61	30.14	2.2	13	28	188	1%

83n	05.07.2024 10:35	23.94	63	36	7.53	2.6	13	34	197	13%
83zc	18.10.2024 13:02	0.37	80	46	0.12		*No time resolution*			32%
83zd	23.10.2024 09:54	0.37	80	46	0.12		*No time resolution*			42%
83ze	23.10.2024 11:35	0.37	80	46	0.12		*No time resolution*			45%
83zf	23.10.2024 13:19	0.37	80	46	0.12		*No time resolution*			48%

*First run with new tubing. Excluded from calculation of average time to completion as outlier.
‡Excluded from Figure 3 (main text) as outlier.

Table S2. Experiment overview for 3 mm diameter tubing. The experiment conducted in Karlsruhe (Germany) is listed as last entry, below the experiments in Brisbane (Australia).

**Experiment**	**Start**	**Circulation time (min)**	**Exposure time (min)**	**Flow rate (mL min^{-1})**	**Flow speed (m min^{-1})**	**Average UV-index[6]**	**Time to completion (min)**	**Time to completion UV index normalised**	**Size at completion (nm)**	**Relative yield (%)**
83s*	19.07.2024 11:50	115	75	53.20	7.53		*Sample degradation*			36%
83t	25.07.2024 12:10	126	83	26.60	3.76	4.1	27	112	234	33%
83u	29.08.2024 12:42	119	78	53.20	7.53	5.2	17	90	236	27%
83v	02.09.2024 13:16	91	60	106.40	15.05	5.2	11	57	235	33%
83x	05.09.2024 11:22	45	30	212.80	30.11		*No yield*			0%
83y	10.09.2024 10:15	60	40	159.60	22.58	4.8	10	45	198	13%
83z	17.10.2024 10:15	60	40	1.84	0.26		*No time resolution*			41%
83za	17.10.2024 11:42	76	50	5.32	0.75	8.5	10	85	223	37%
83zb	18.10.2024 11:06	68	45	0.07	0.01		*No time resolution*			36%
Karlsruhe** (Germany)	2.10.2025 14:48	147	82	120.40	17.00	2.5**	14	34	226	23%

*First run with new tubing.

**The average UV index for the experiment in Karlsruhe (Germany) was calculated from minute wise, simulated UV index data provided by the Apple weather app for the location Eggenstein-Leopoldshafen. It was a clear sky day, which makes the prediction of the UV index accurate. The obtained UV index was also compared to the closest UV index measurement stations around in Germany on that day (Federal Office for Radiation Protection: Stuttgart, Tholey, Weißenburg)[7], which showed excellent agreement.

5.2. Determination of time to completion by DLS

5.2.1. 2 mm diameter tubing

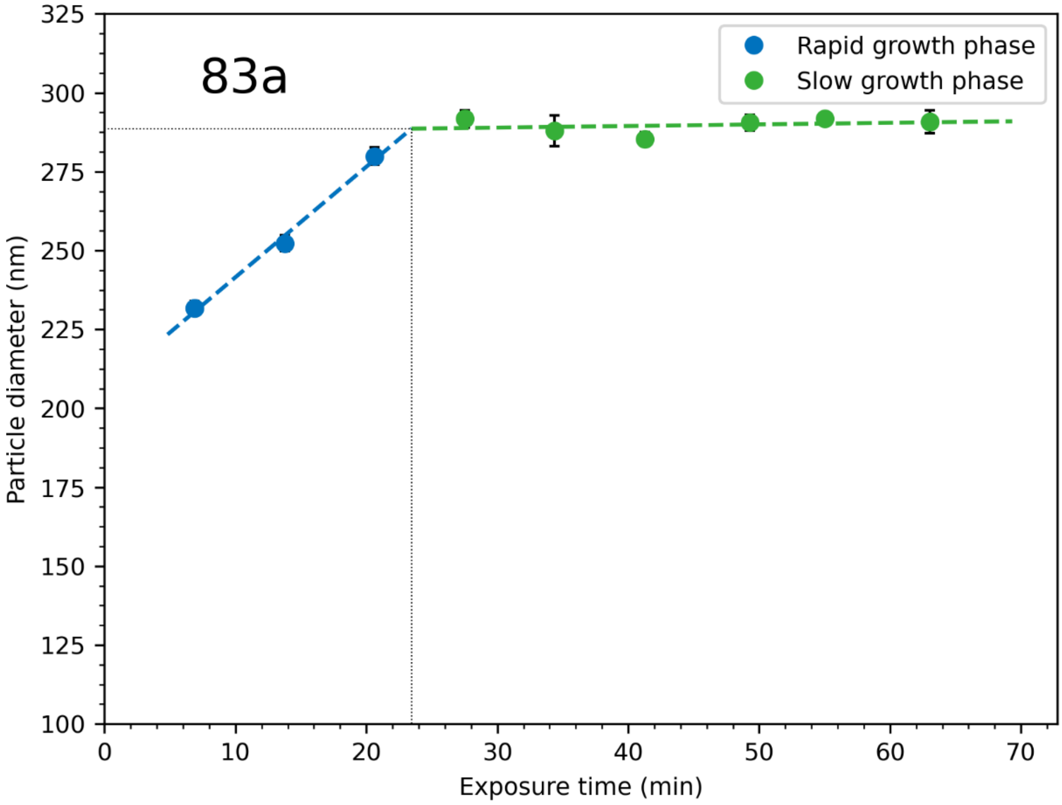

Figure S3. Time of completion and size at completion determination from DLS size analysis. Top left: experiment number.

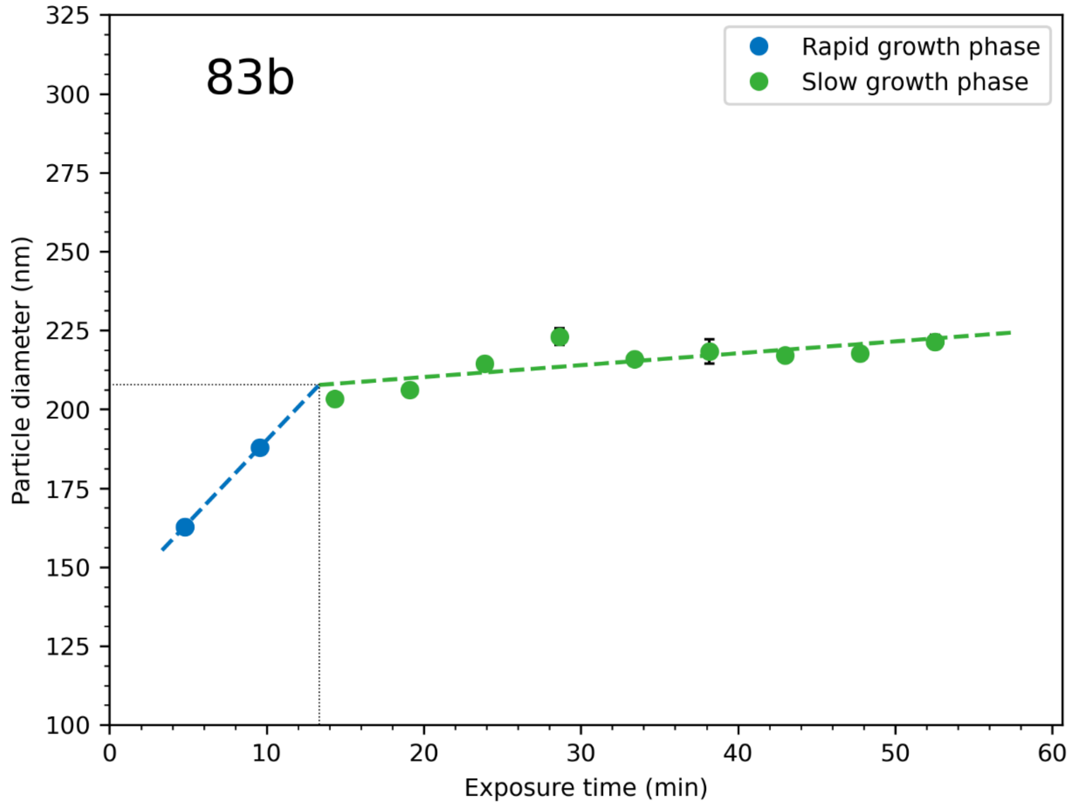

Figure S4. Time of completion and size at completion determination from DLS size analysis. Top left: experiment number.

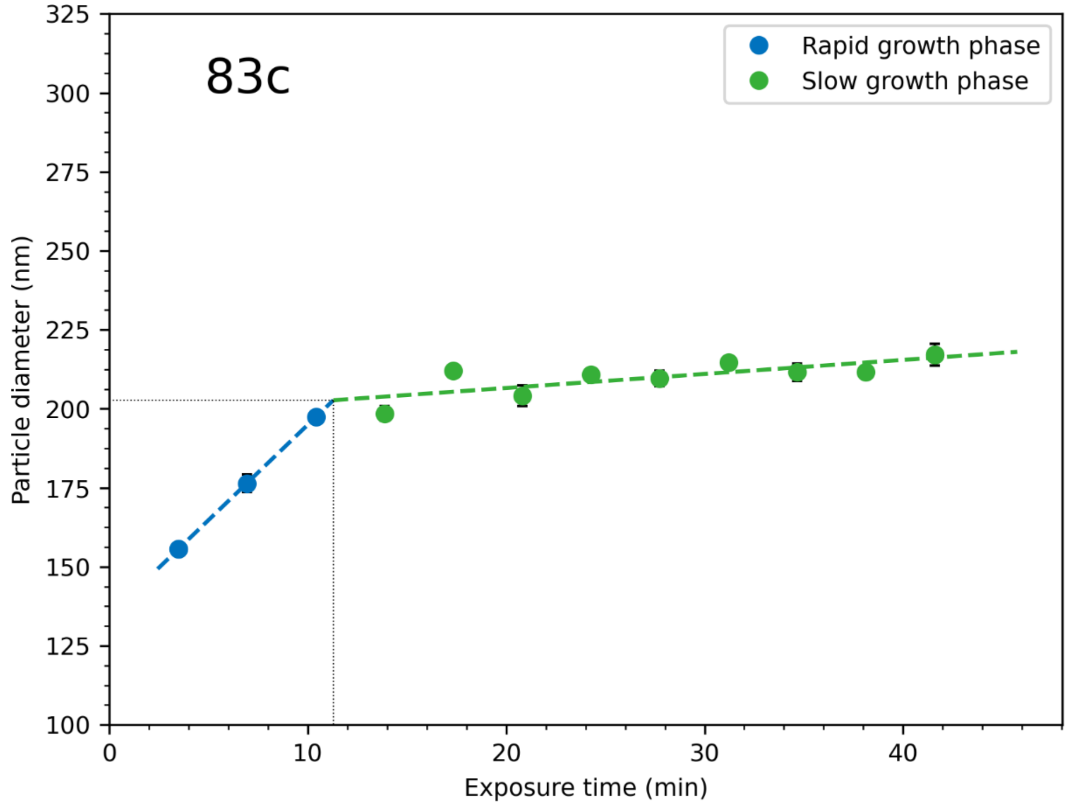

Figure S5. Time of completion and size at completion determination from DLS size analysis. Top left: experiment number.

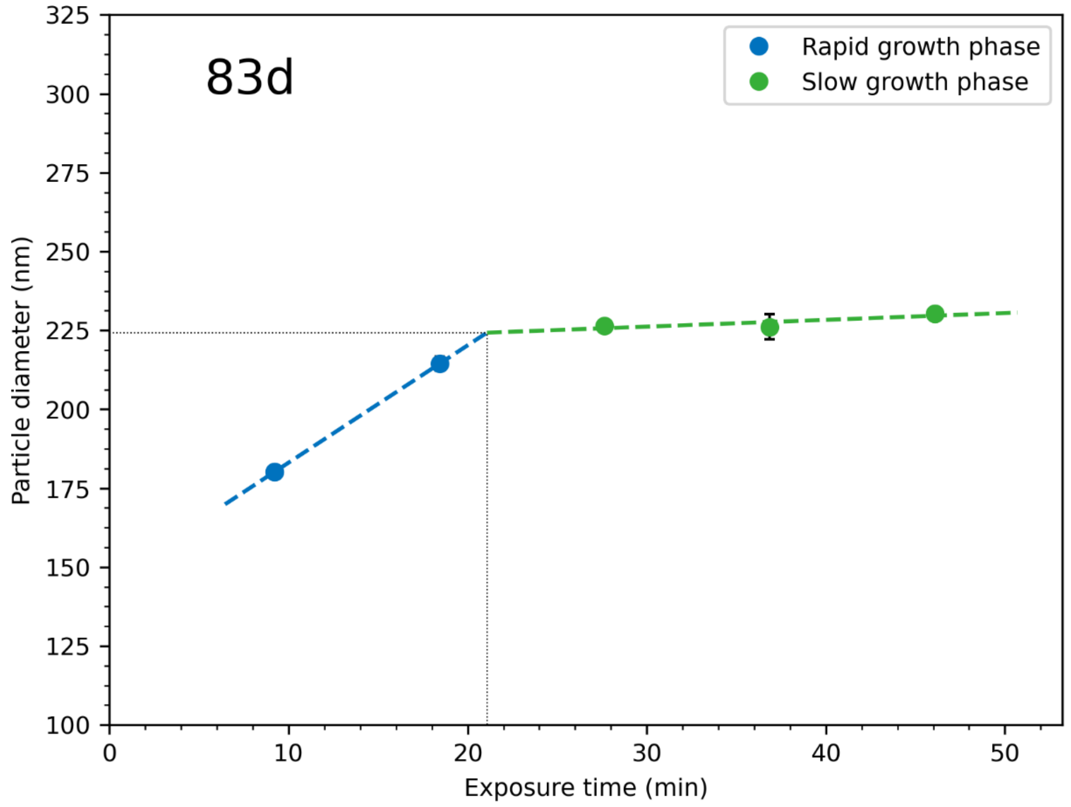

Figure S6. Time of completion and size at completion determination from DLS size analysis. Top left: experiment number.

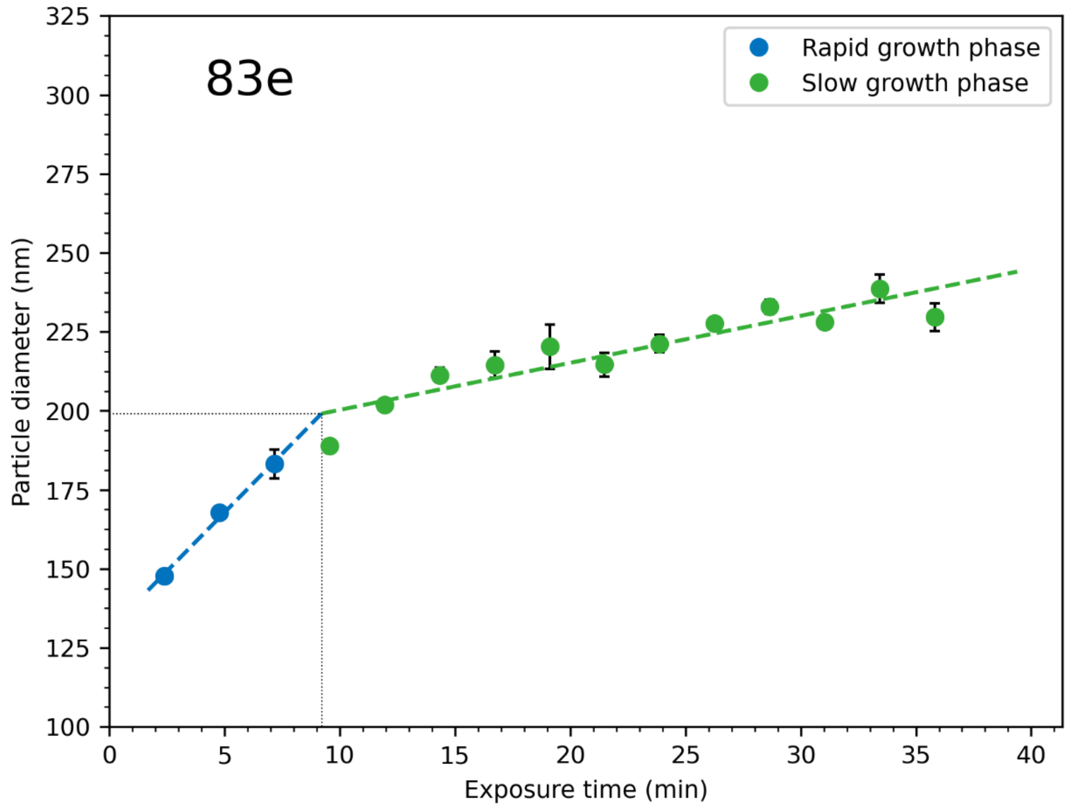

Figure S7. Time of completion and size at completion determination from DLS size analysis. Top left: experiment number.

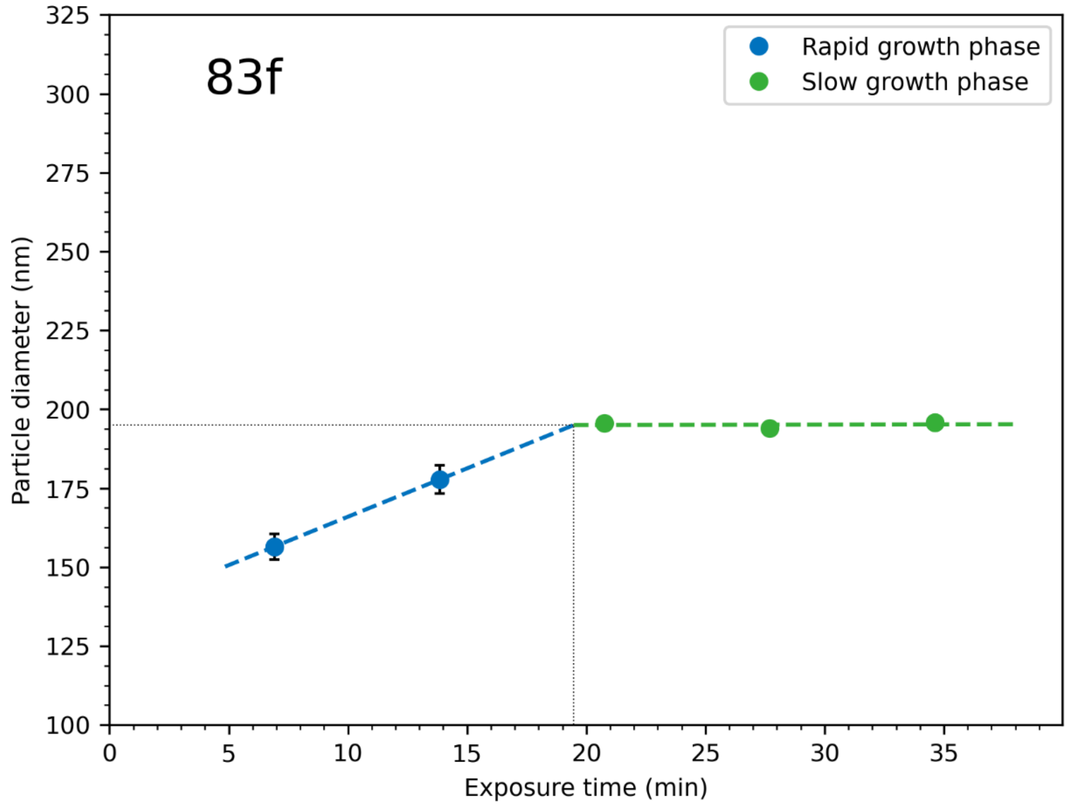

Figure S8. Time of completion and size at completion determination from DLS size analysis. Top left: experiment number.

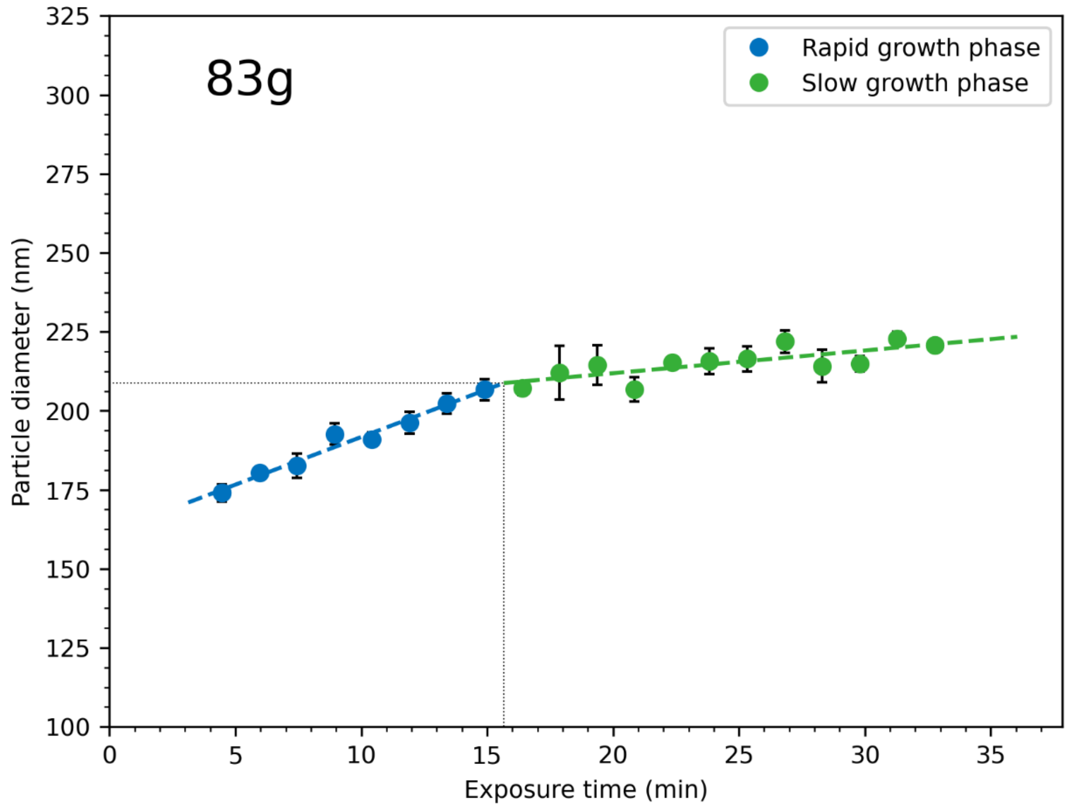

Figure S9. Time of completion and size at completion determination from DLS size analysis. Top left: experiment number.

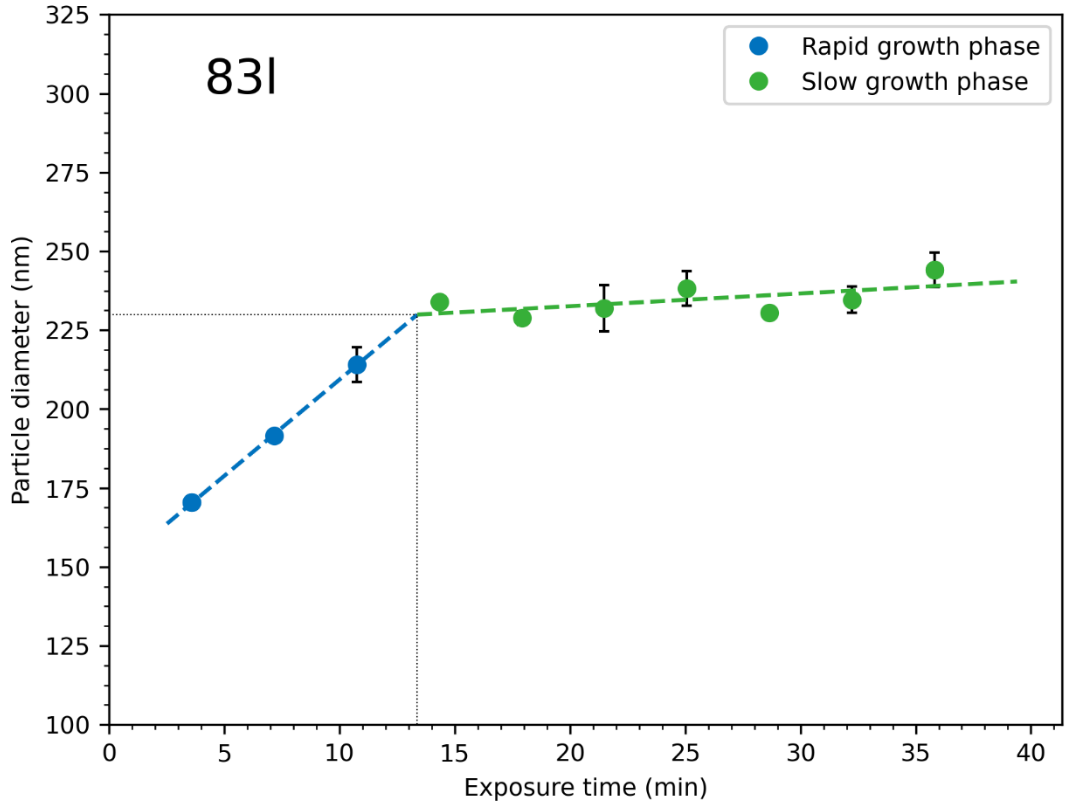

Figure S10. Time of completion and size at completion determination from DLS size analysis. Top left: experiment number.

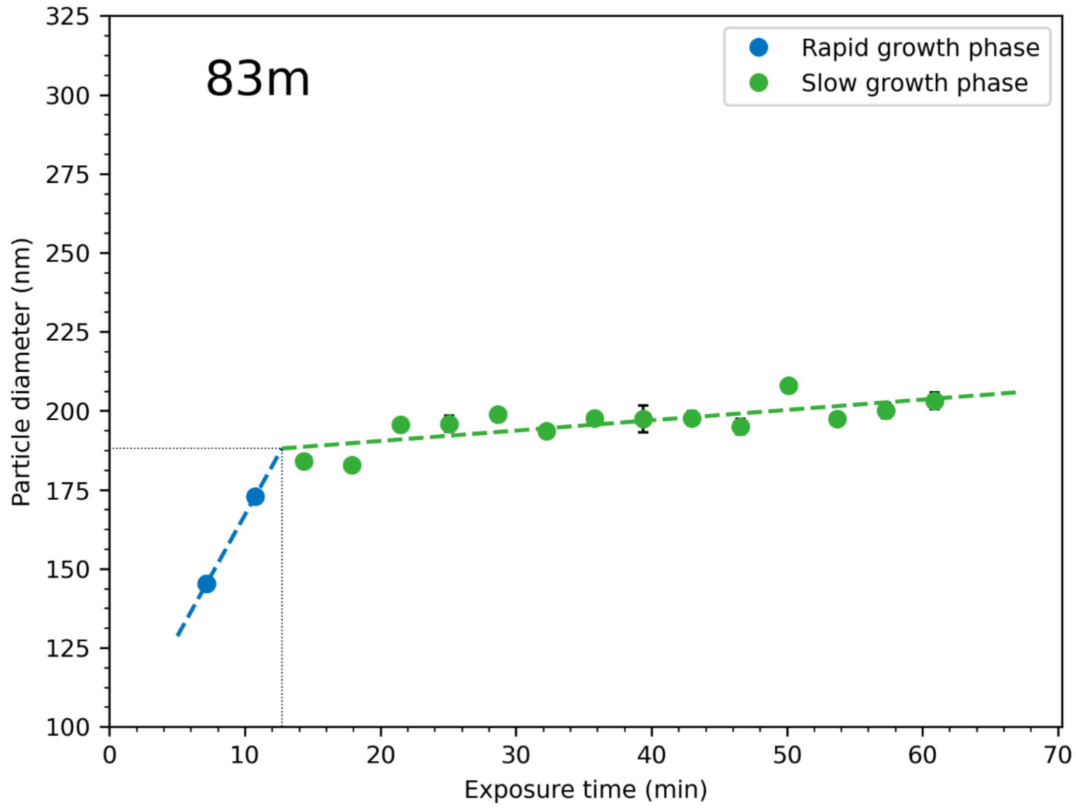

Figure S11. Time of completion and size at completion determination from DLS size analysis. Top left: experiment number.

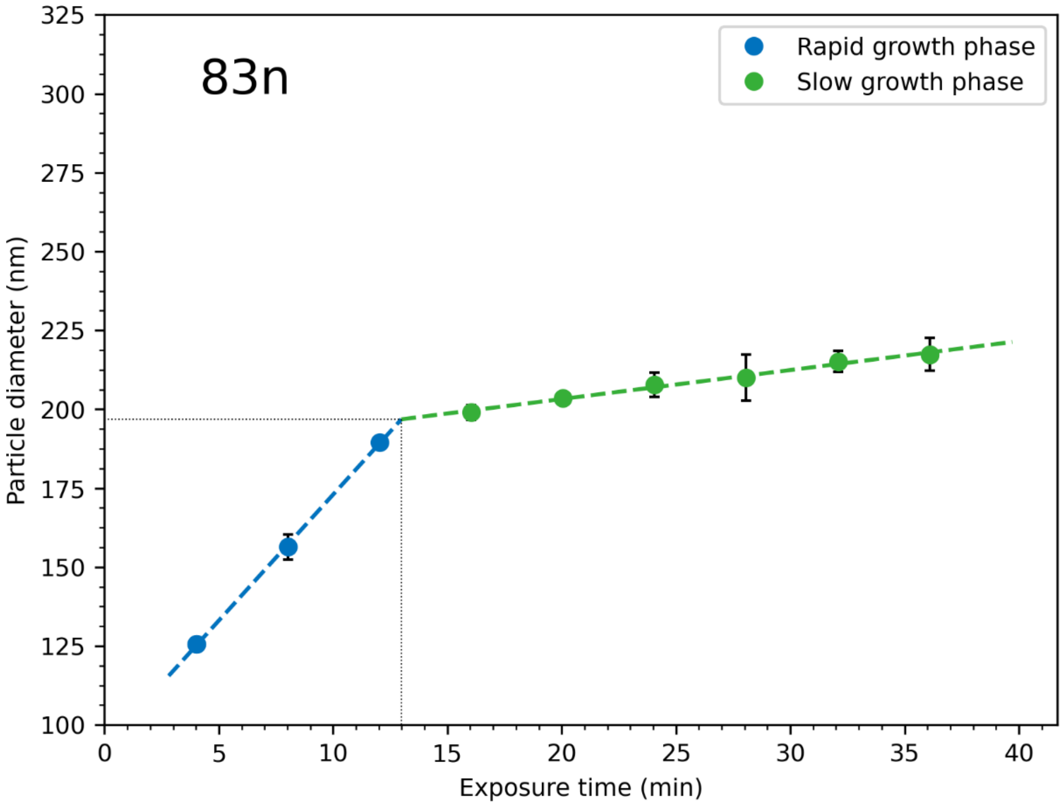

Figure S12. Time of completion and size at completion determination from DLS size analysis. Top left: experiment number.

5.2.2. 3 mm diameter tubing

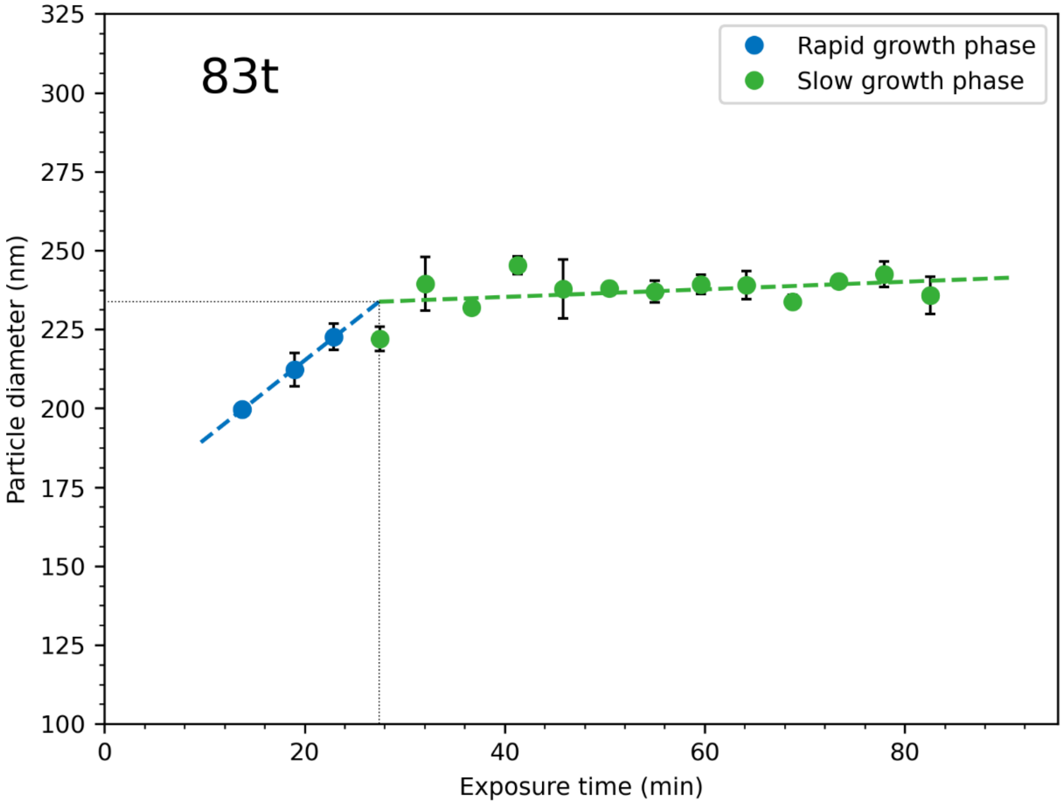

Figure S13. Time of completion and size at completion determination from DLS size analysis. Top left: experiment number.

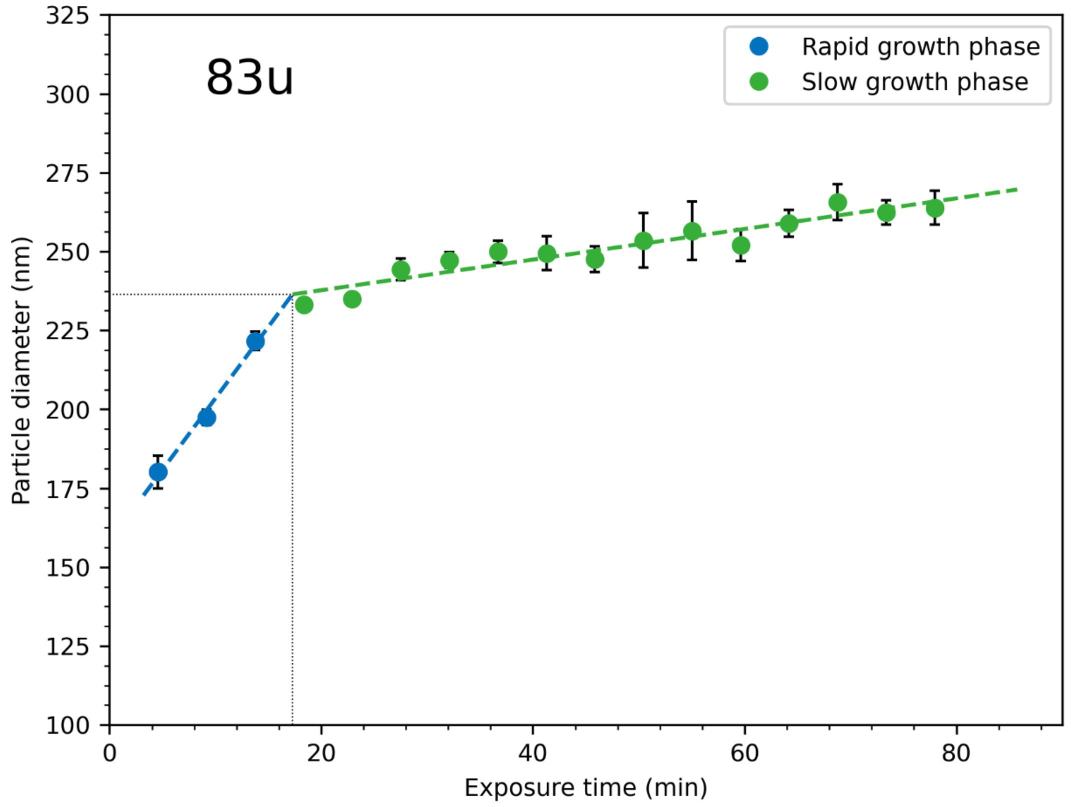

Figure S14. Time of completion and size at completion determination from DLS size analysis. Top left: experiment number.

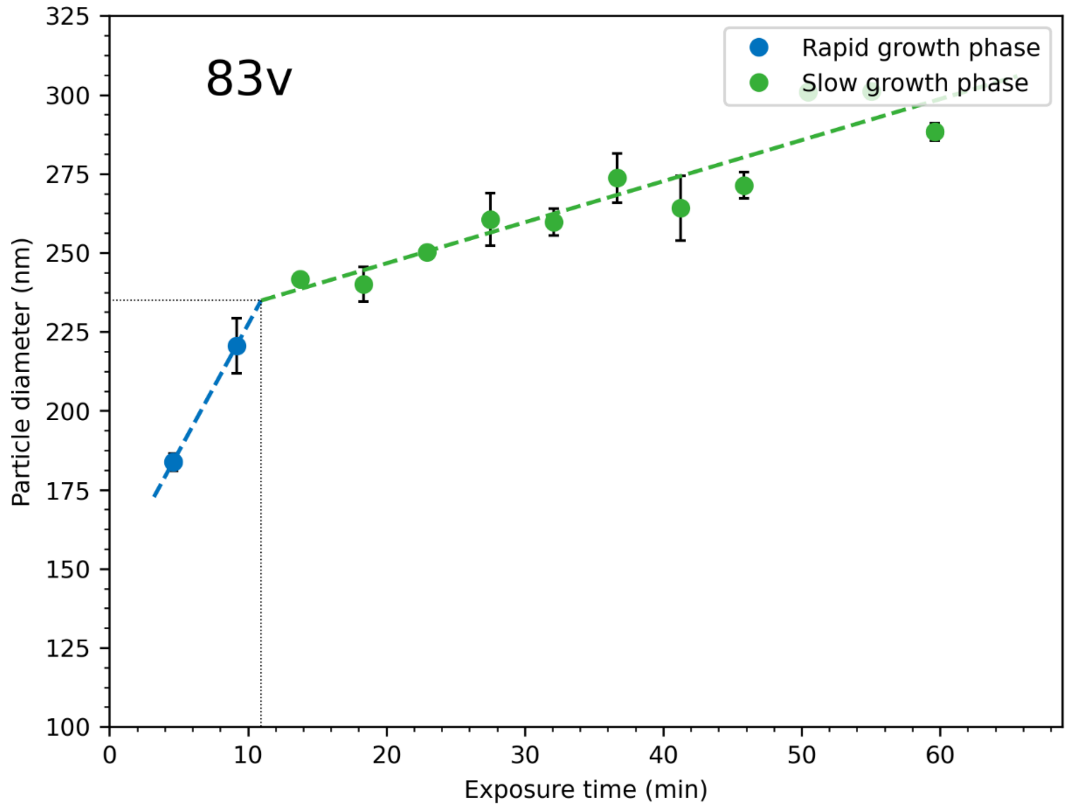

Figure S15. Time of completion and size at completion determination from DLS size analysis. Top left: experiment number.

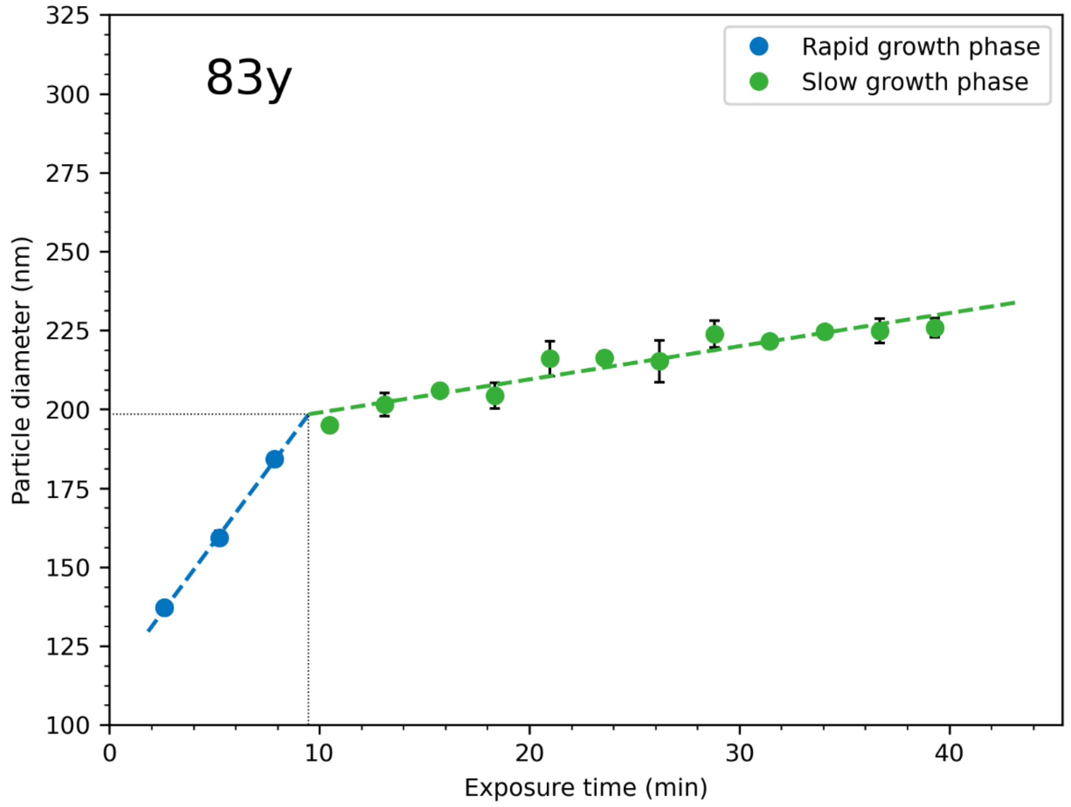

Figure S16. Time of completion and size at completion determination from DLS size analysis. Top left: experiment number.

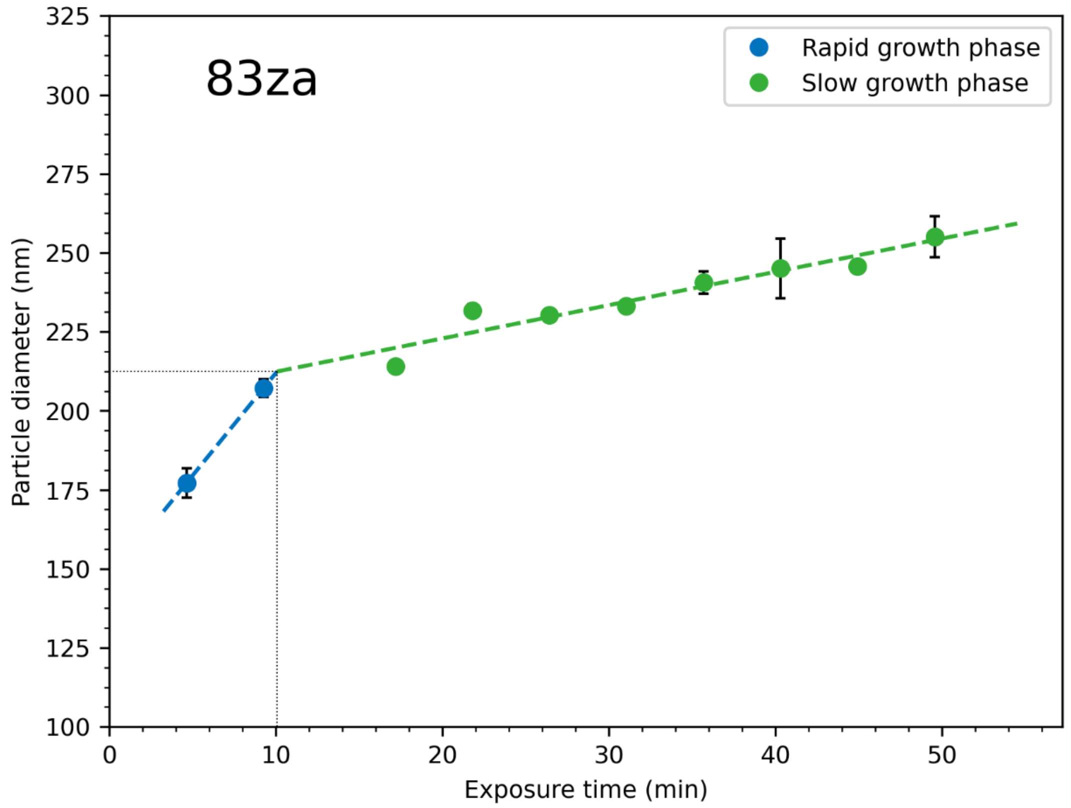

Figure S17. Time of completion and size at completion determination from DLS size analysis. Top left: experiment number.

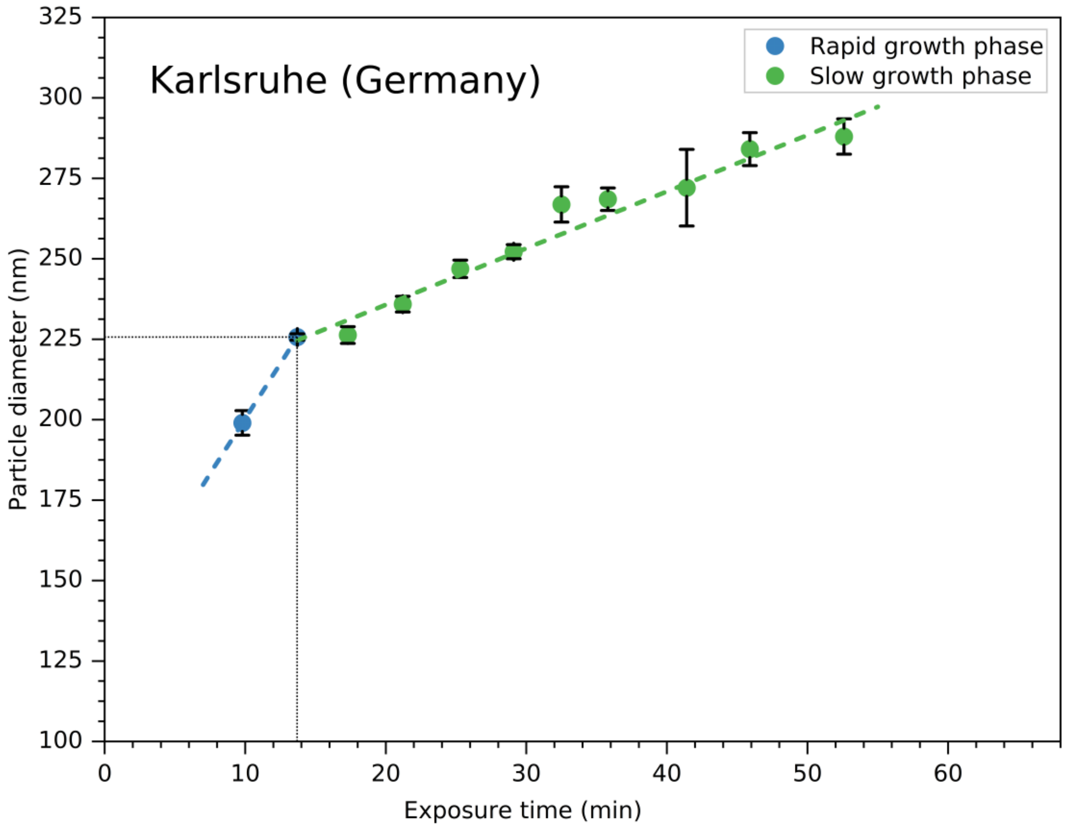

Figure S18. Time of completion and size at completion determination from DLS size analysis for the experiment conducted in Karlsruhe (Germany).

5.3. Size and dispersity analysis by SEM

Below are the results of the size and dispersity analysis by SEM listed. This comprises the overviews in Table S3 and Table S4, as well as the plots of the dispersity as a function of the flow speed in Figure S19 and Figure S20. The corresponding SEM images with segmented particles are shown in Section S5.3.1 and 5.3.2, and their histograms in Section S5.3.3 and 5.3.4.

Table S3. Results of the SEM size and dispersity analysis for 2 mm diameter tubing.

Experiment	Flow speed (m min^{-1})	Diameter mean (nm)	N particles measured	Đ
83d	0.57	185	1216	0.037
83a*	0.75	206	198	0.035
83f	0.75	175	389	0.046
83b	1.13	175	530	0.034
83c	1.51	182	1864	0.045
83e	2.26	184	1080	0.027
83g	3.77	175	683	0.020
83n	7.53	213	546	0.014
83l	15.07	263	703	0.014

*First run with new tubing

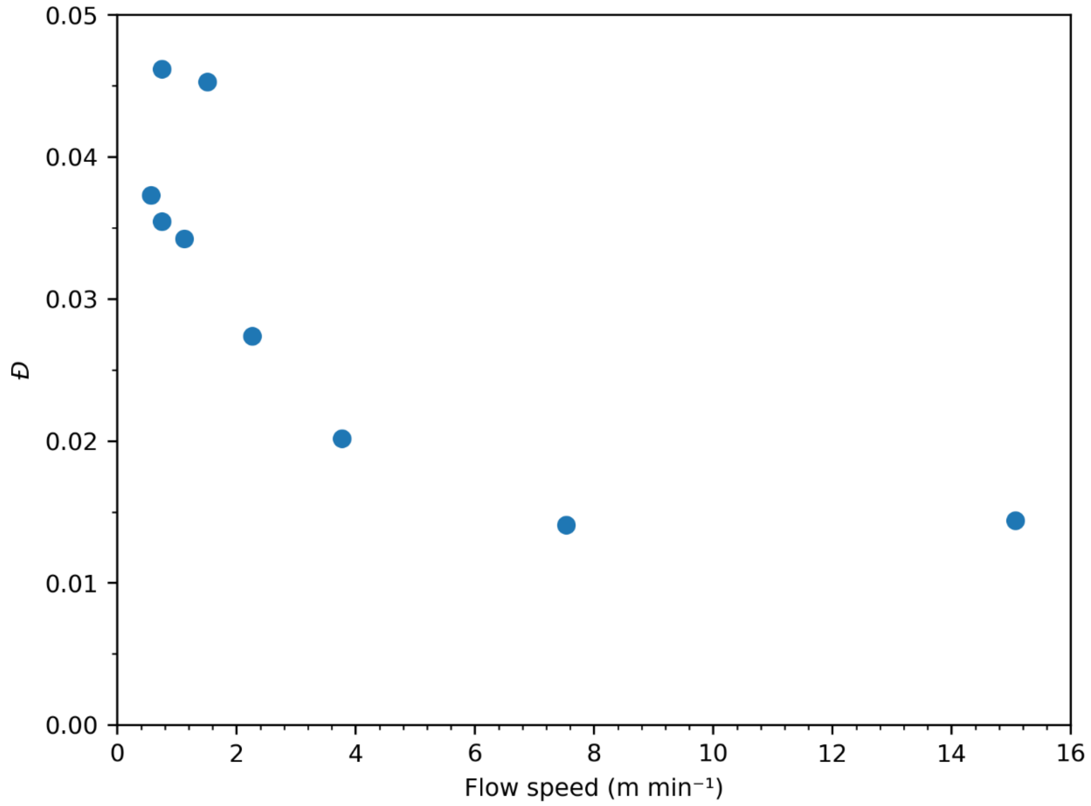

Figure S19. Dispersity against flow speed from the SEM analysis of the 2 mm samples.

Table S4. Results of the SEM size and dispersity analysis for 3 mm diameter tubing.

Experiment	Flow speed (m min^{-1})	Diameter mean (nm)	N particles measured	Đ
83zb	0.01	312	498	0.029
83z	0.26	362	377	0.034
83za	0.75	286	448	0.024
83t	3.76	257	707	0.021
83s*	7.53	426	224	0.008
83u	7.53	291	647	0.020
83v	15.05	318	529	0.011
83y	22.58	242	402	0.029
Karlsruhe (Germany)	17.00	234	266	0.011

*First run with new tubing

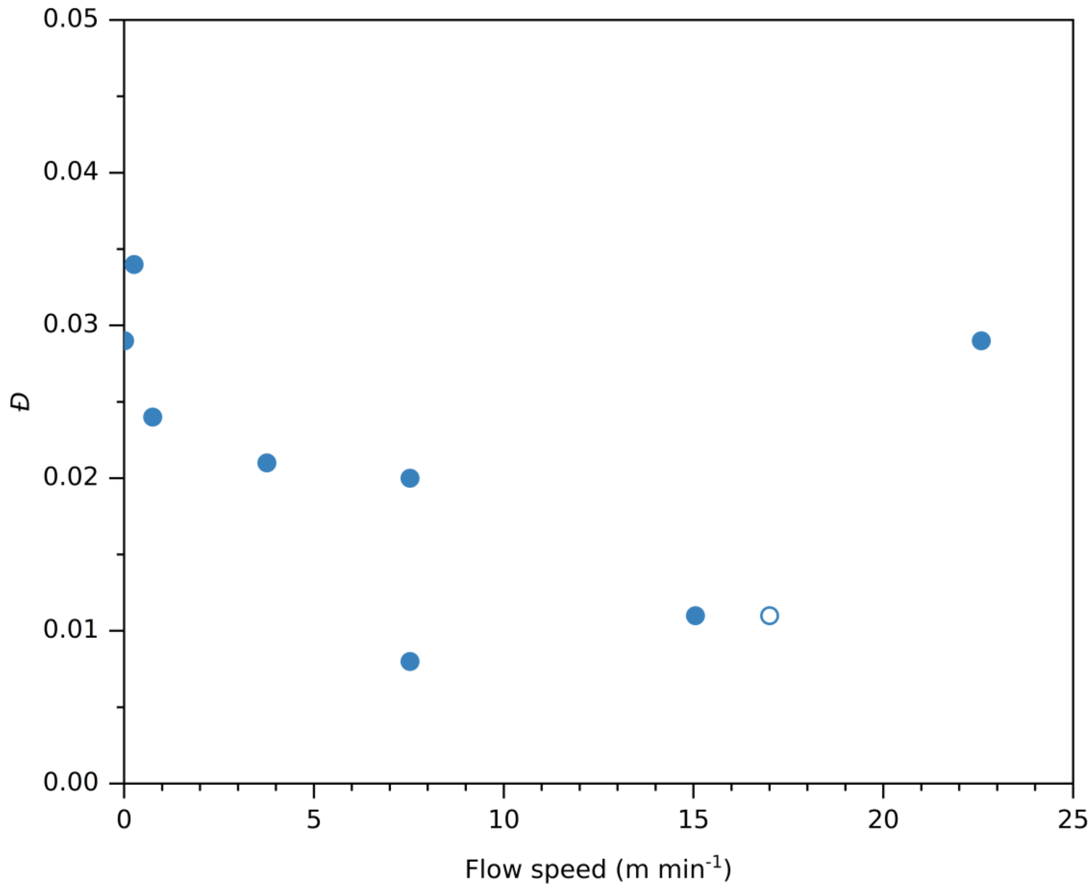

Figure S20. Dispersity vs flow speed from the SEM analysis of the 3 mm samples. The empty data point was obtained with an identical reactor set-up with 3 mm tubing in Karlsruhe (Germany).

5.3.1. Segmented SEM images: 2 mm diameter tubing

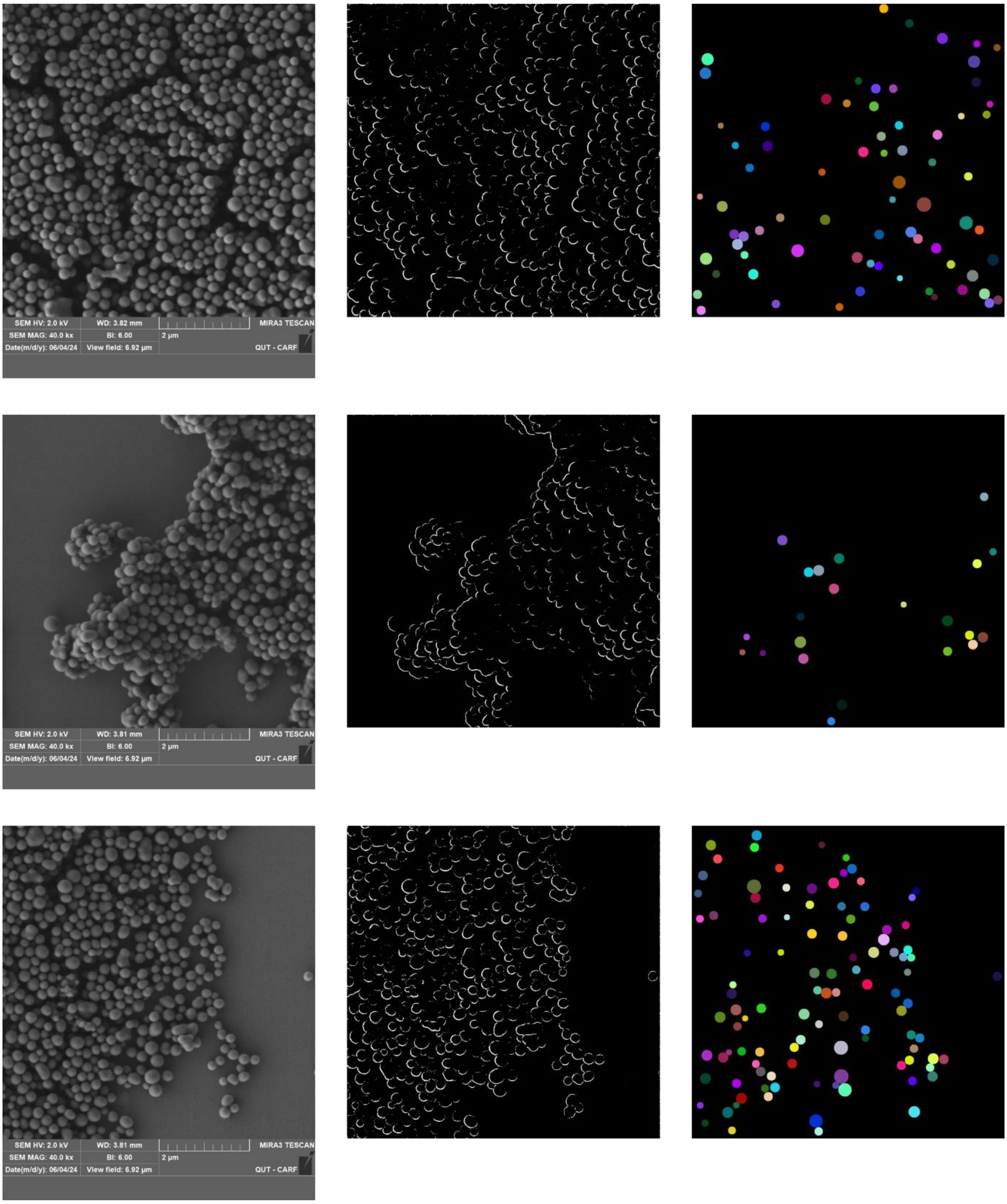

Figure S21. SEM size analysis via automated segmentation with edge detection and Circle Hough Transform for sample 83a.

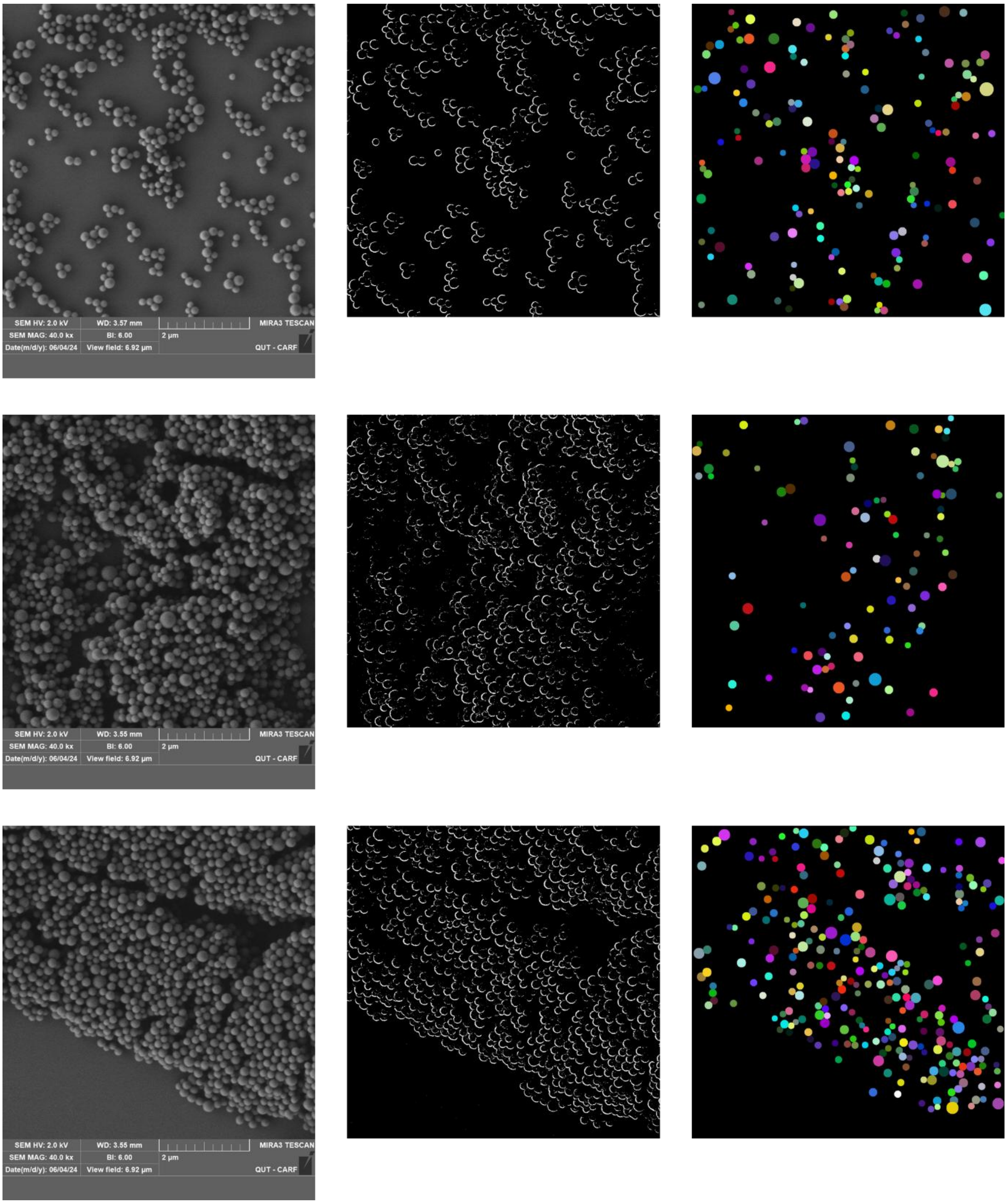

Figure S22. SEM size analysis via automated segmentation with edge detection and Circle Hough Transform for sample 83b.

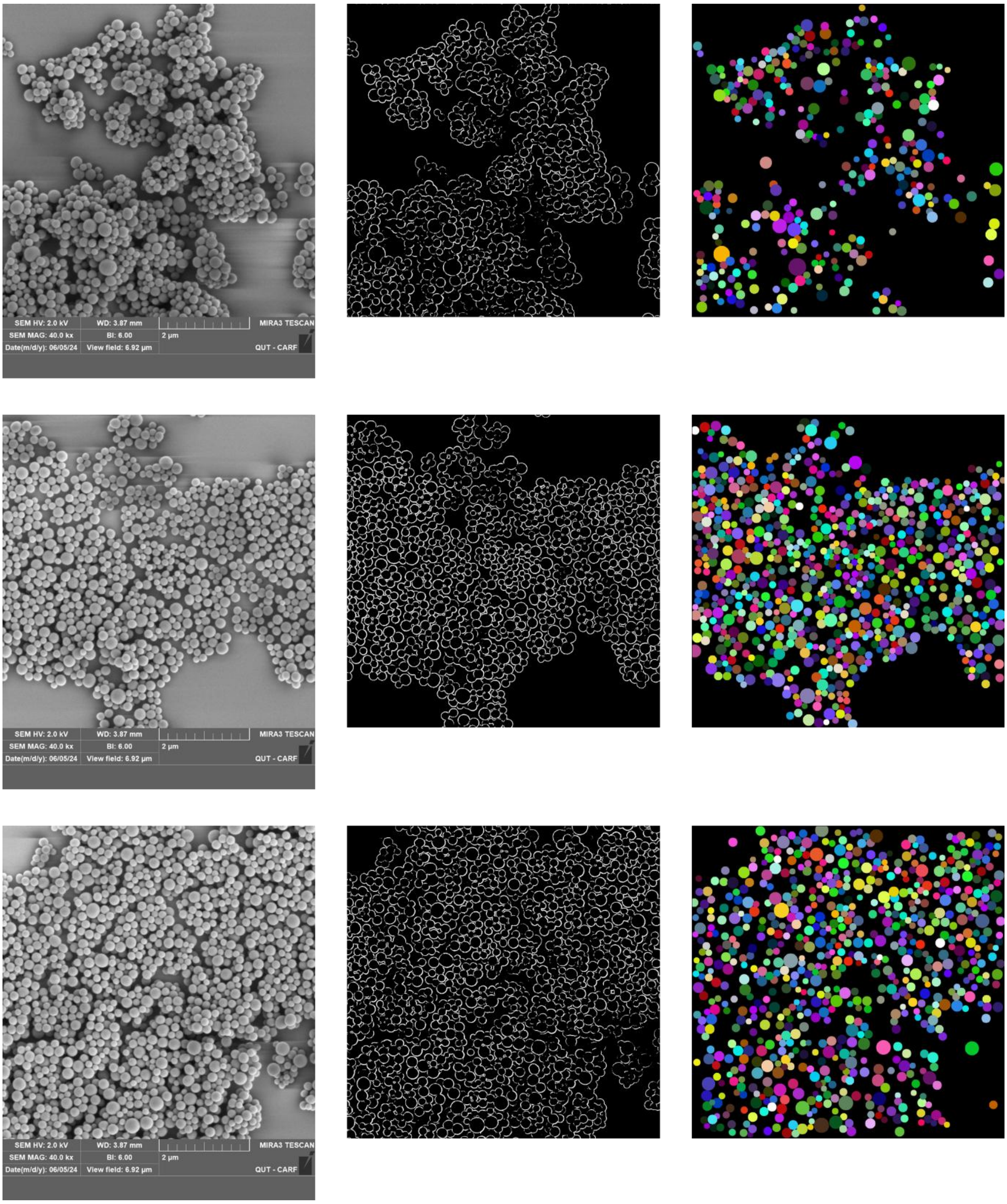

Figure S23. SEM size analysis via automated segmentation with edge detection and Circle Hough Transform for sample 83c.

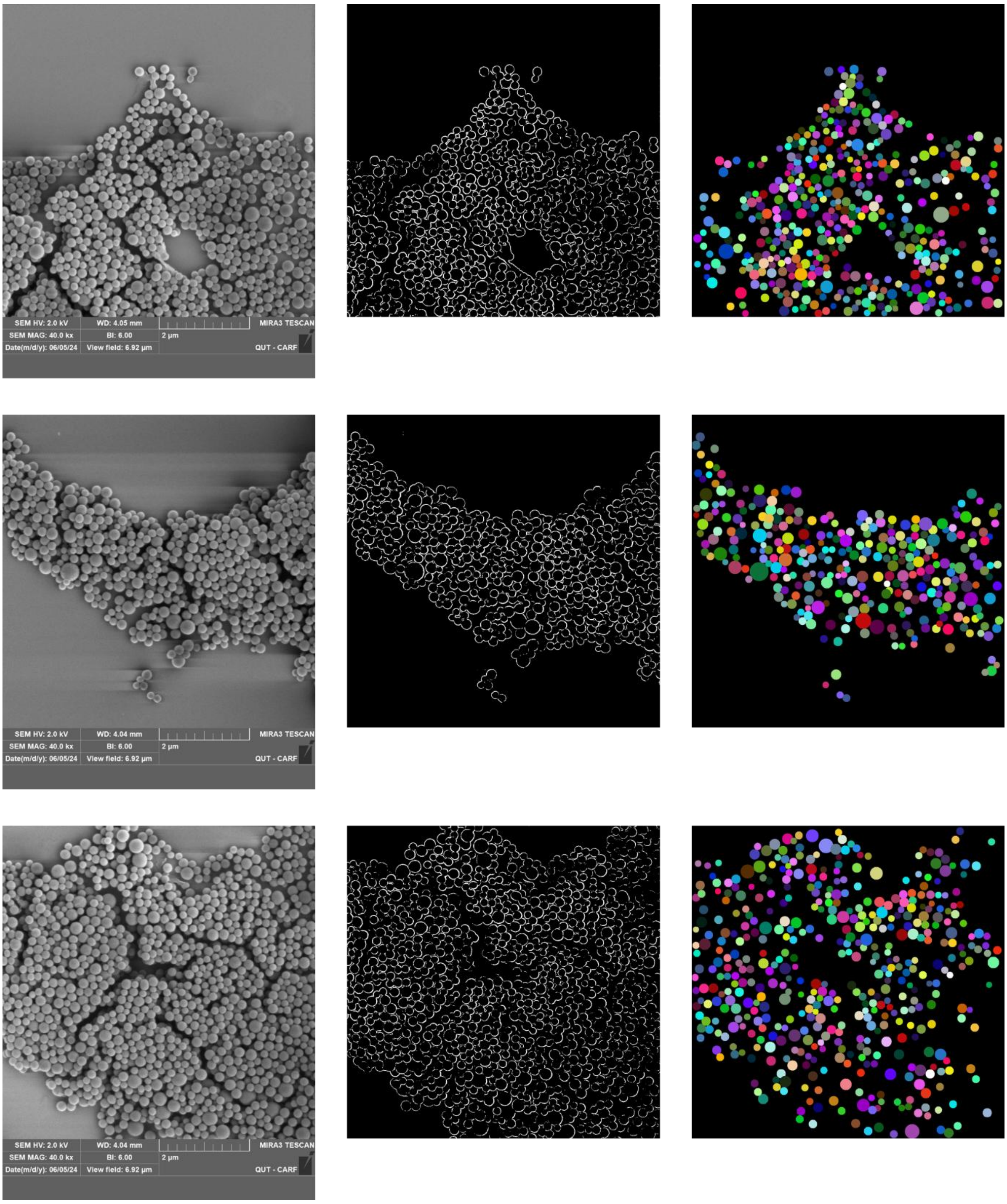

Figure S24. SEM size analysis via automated segmentation with edge detection and Circle Hough Transform for sample 83d.

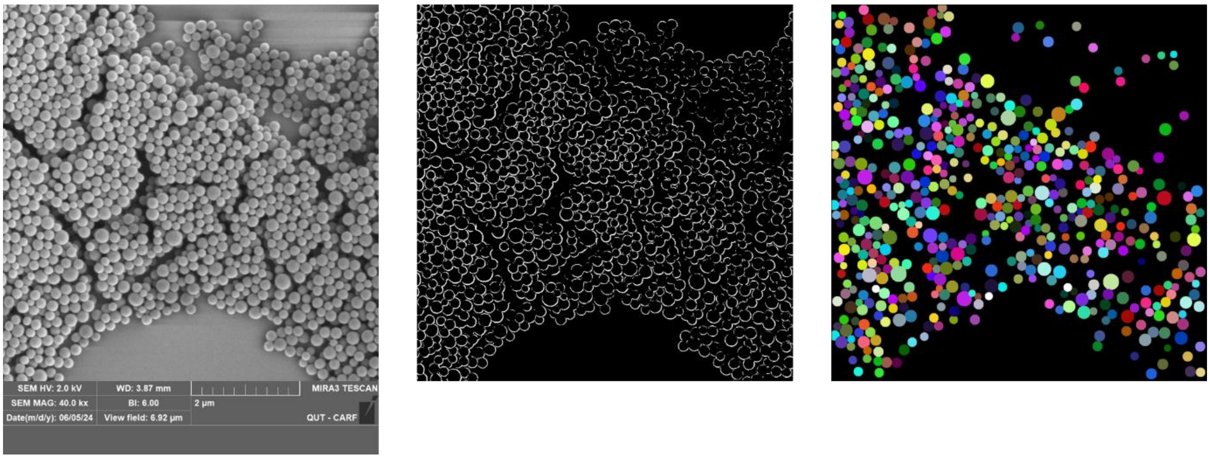

Figure S25. SEM size analysis via automated segmentation with edge detection and Circle Hough Transform for sample 83e.

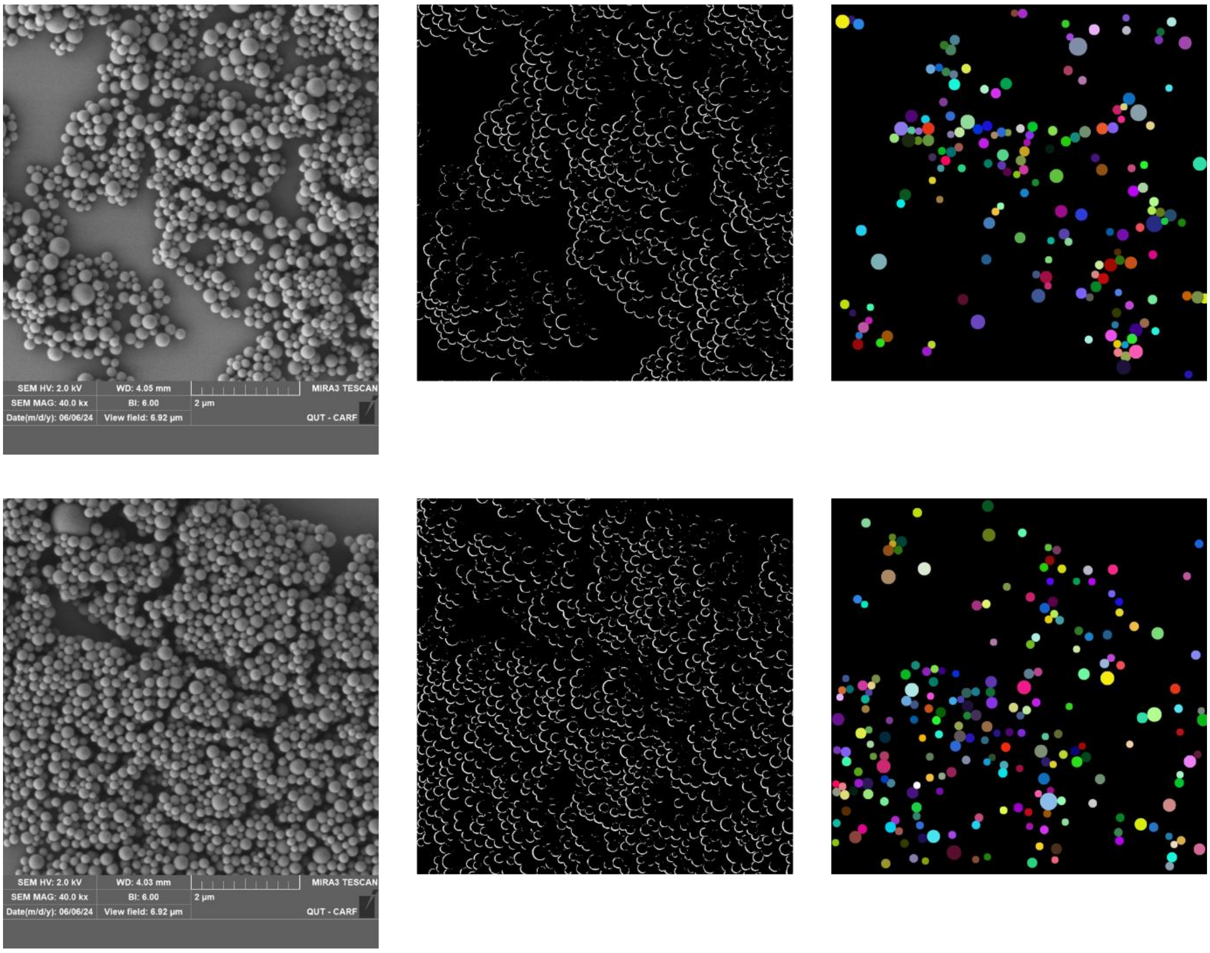

Figure S26. SEM size analysis via automated segmentation with edge detection and Circle Hough Transform for sample 83f.

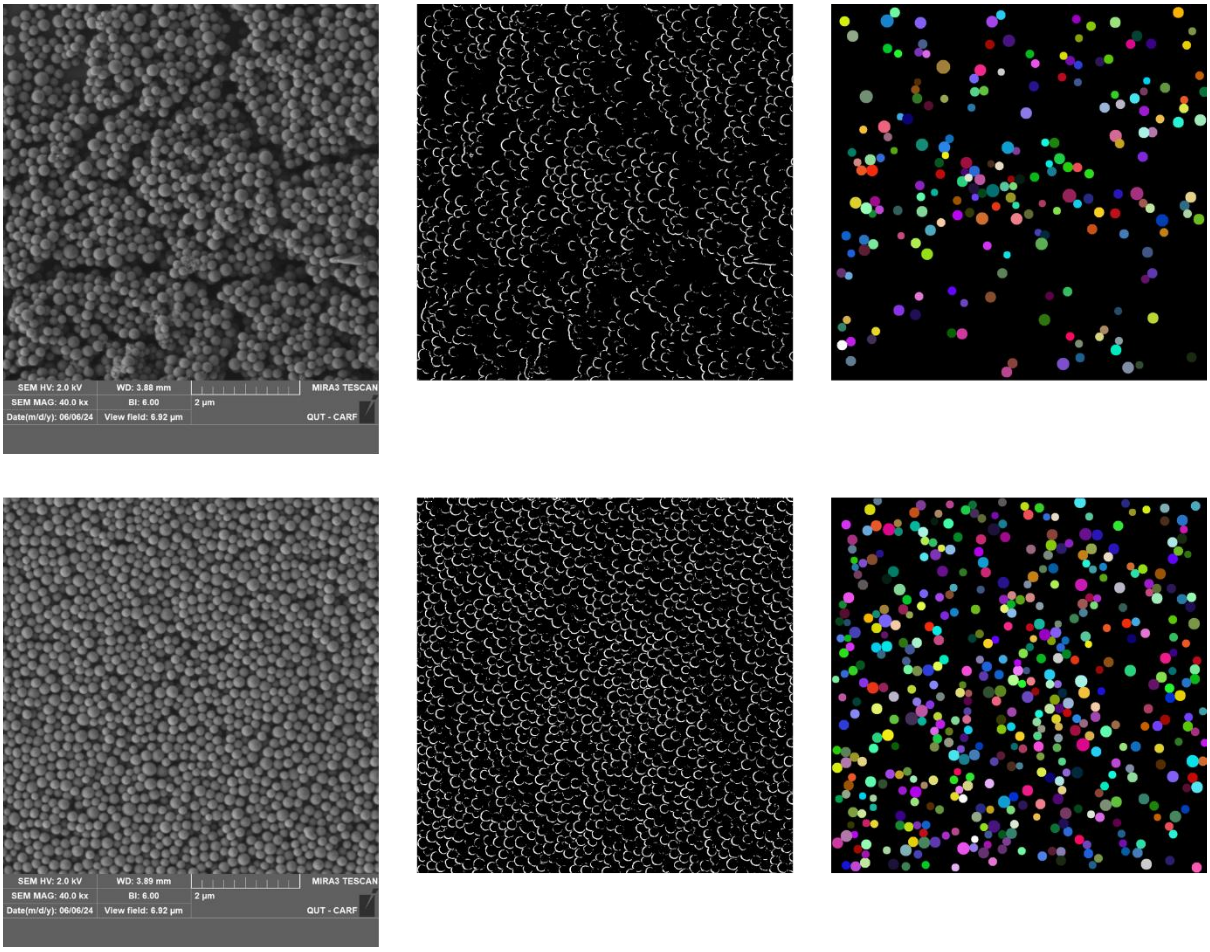

Figure S27. SEM size analysis via automated segmentation with edge detection and Circle Hough Transform for sample 83g.

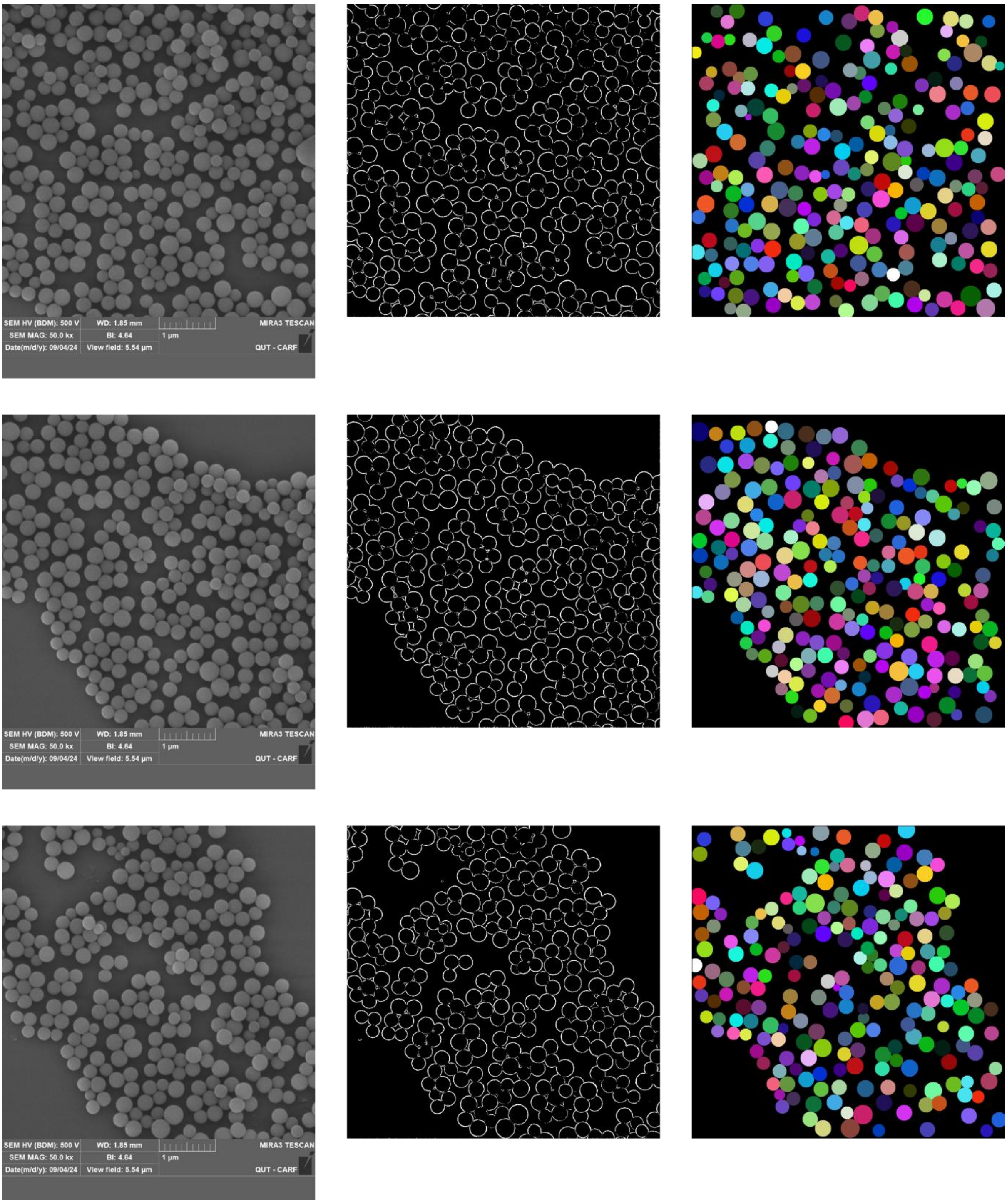

Figure S28. SEM size analysis via automated segmentation with edge detection and Circle Hough Transform for sample 83l.

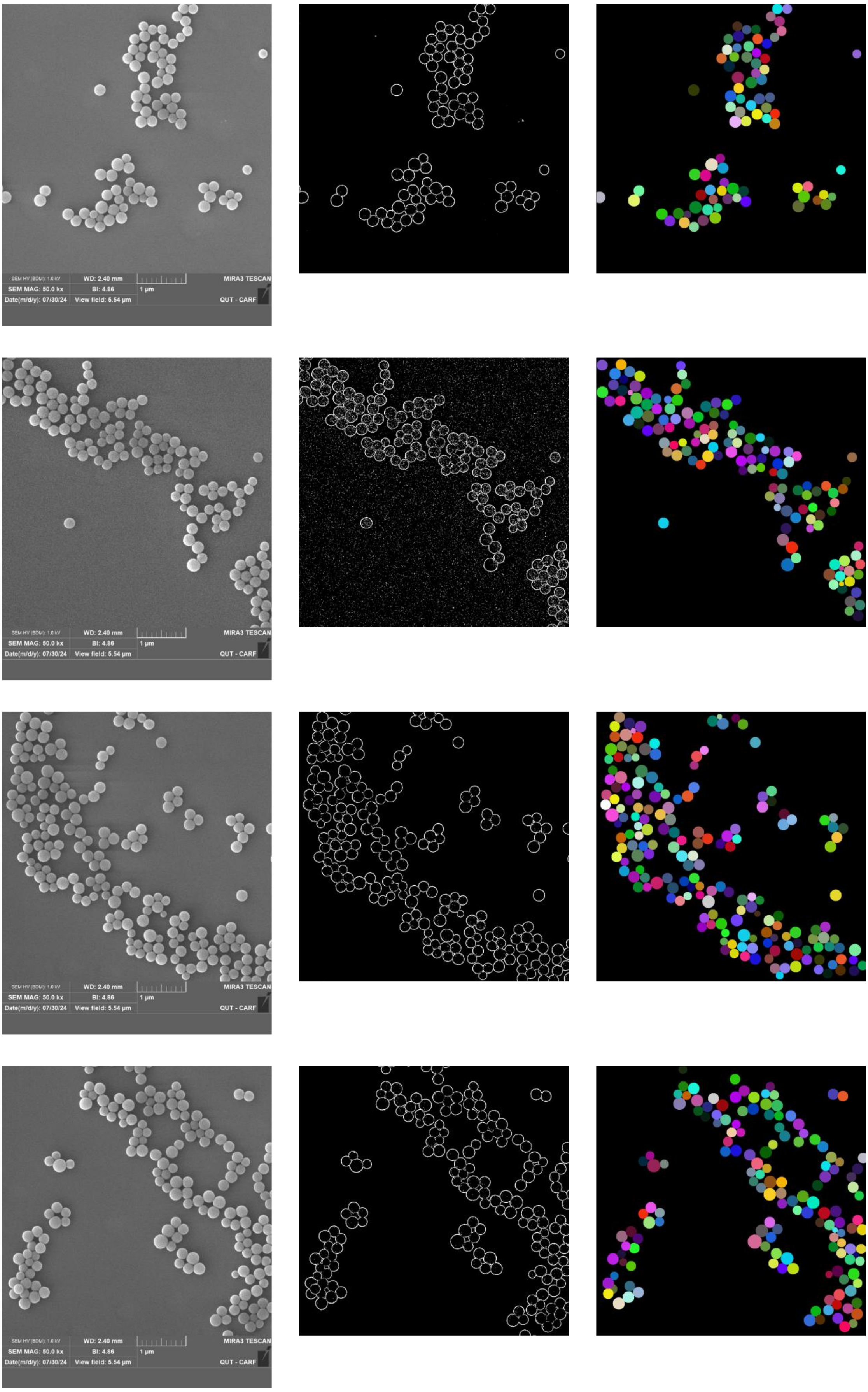

Figure S29. SEM size analysis via automated segmentation with edge detection and Circle Hough Transform for sample 83n.

5.3.2. Segmented SEM images: 3 mm diameter tubing

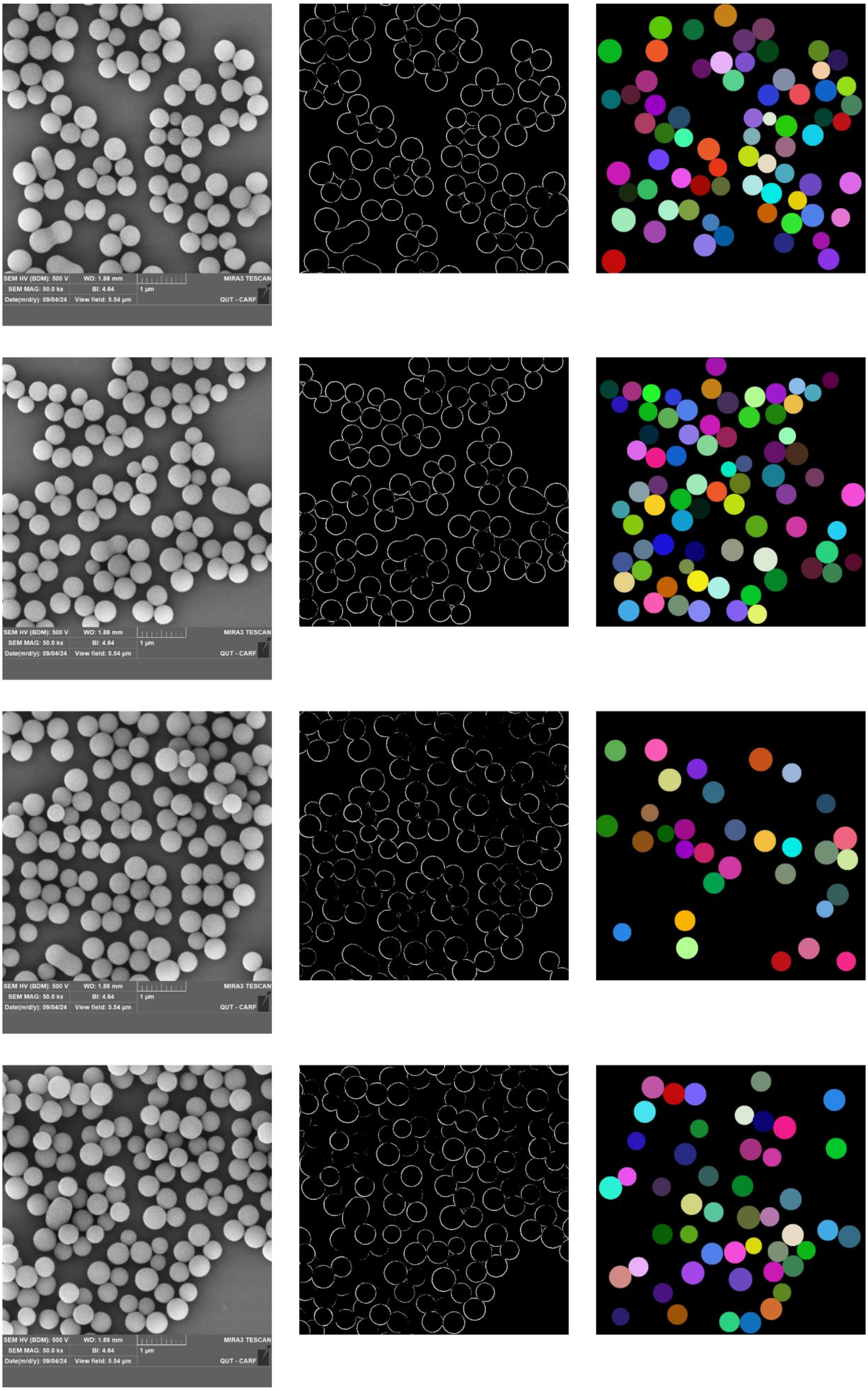

Figure S30. SEM size analysis via automated segmentation with edge detection and Circle Hough Transform for sample 83s.

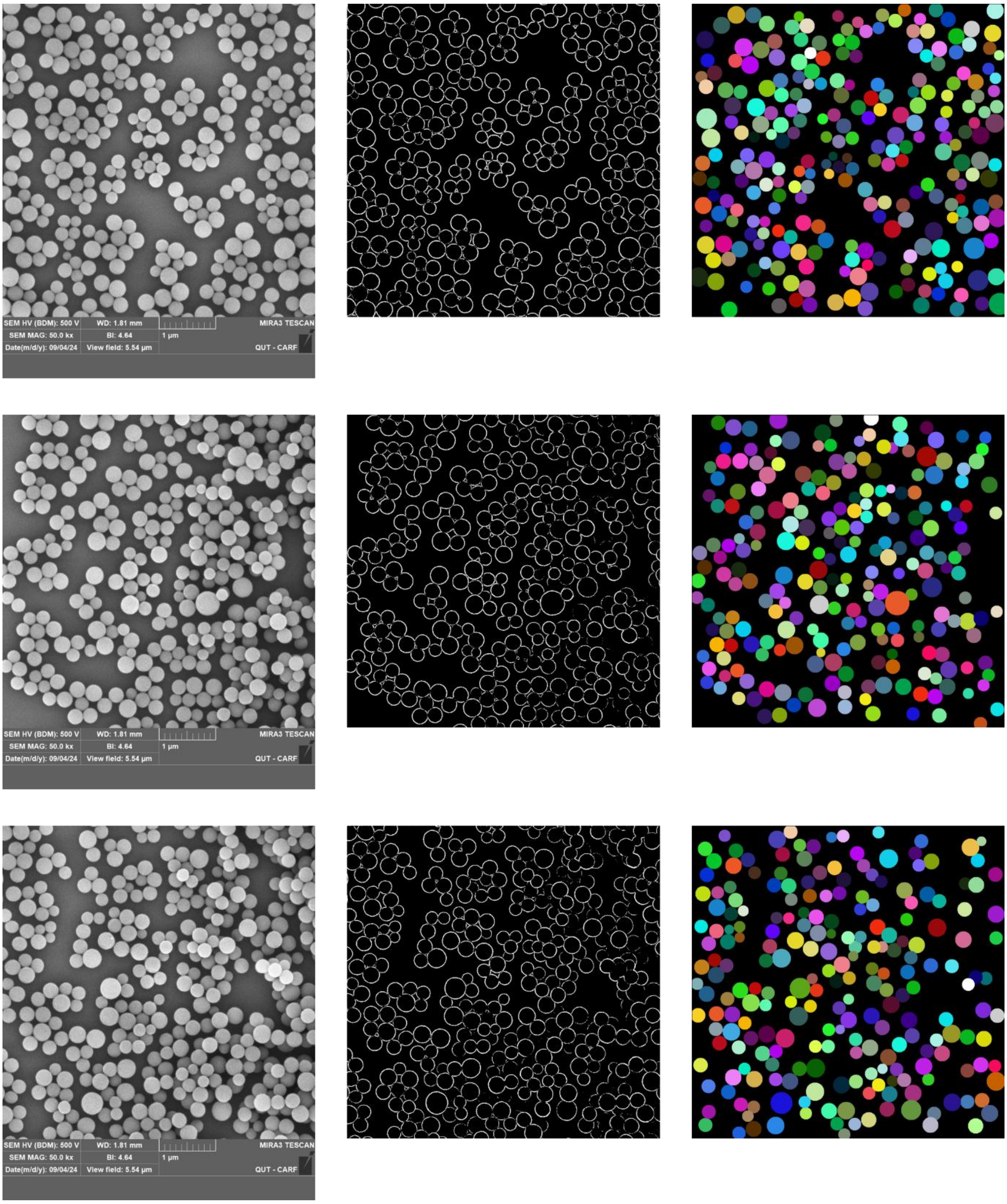

Figure S31. SEM size analysis via automated segmentation with edge detection and Circle Hough Transform for sample 83t.

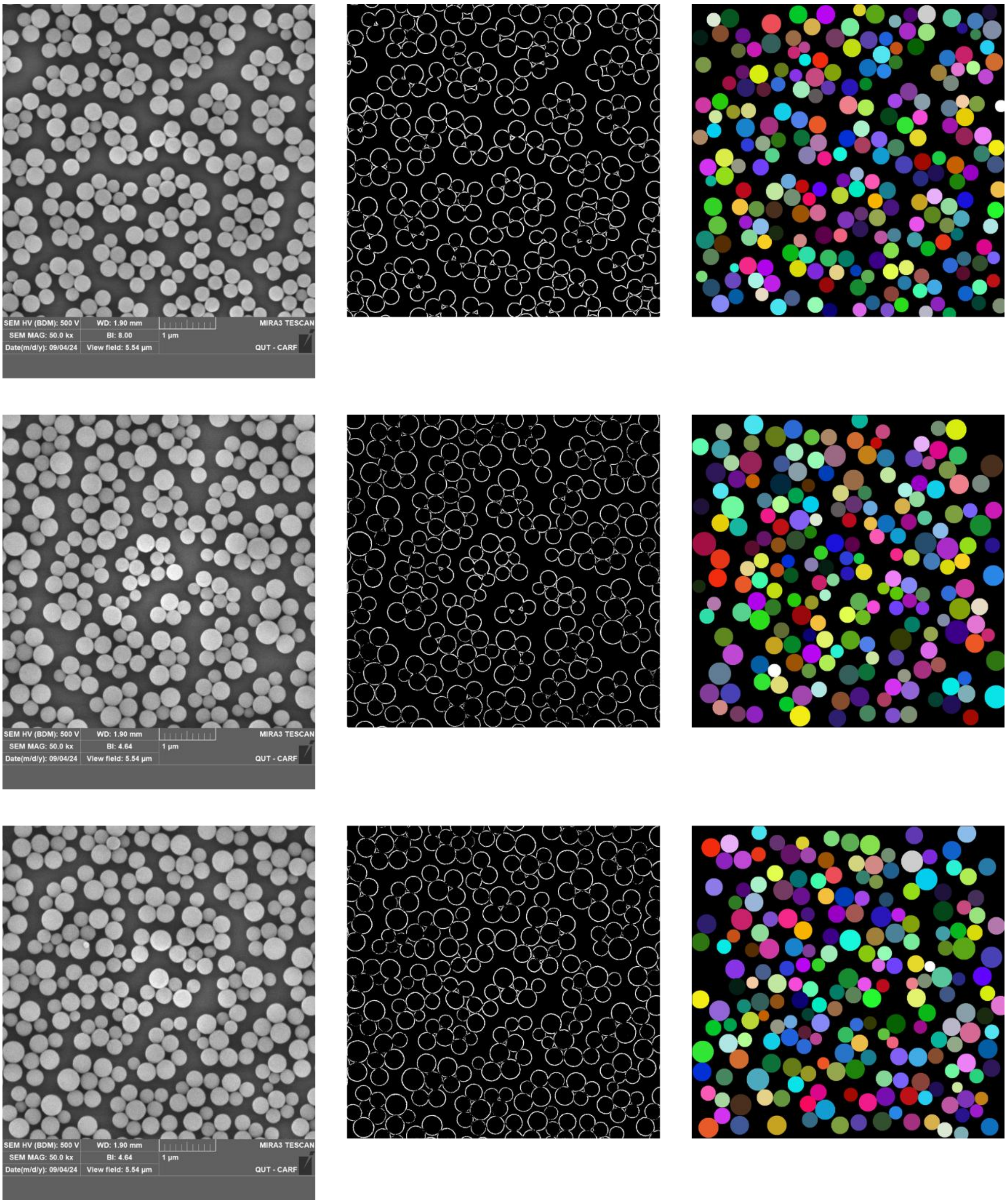

Figure S32. SEM size analysis via automated segmentation with edge detection and Circle Hough Transform for sample 83u

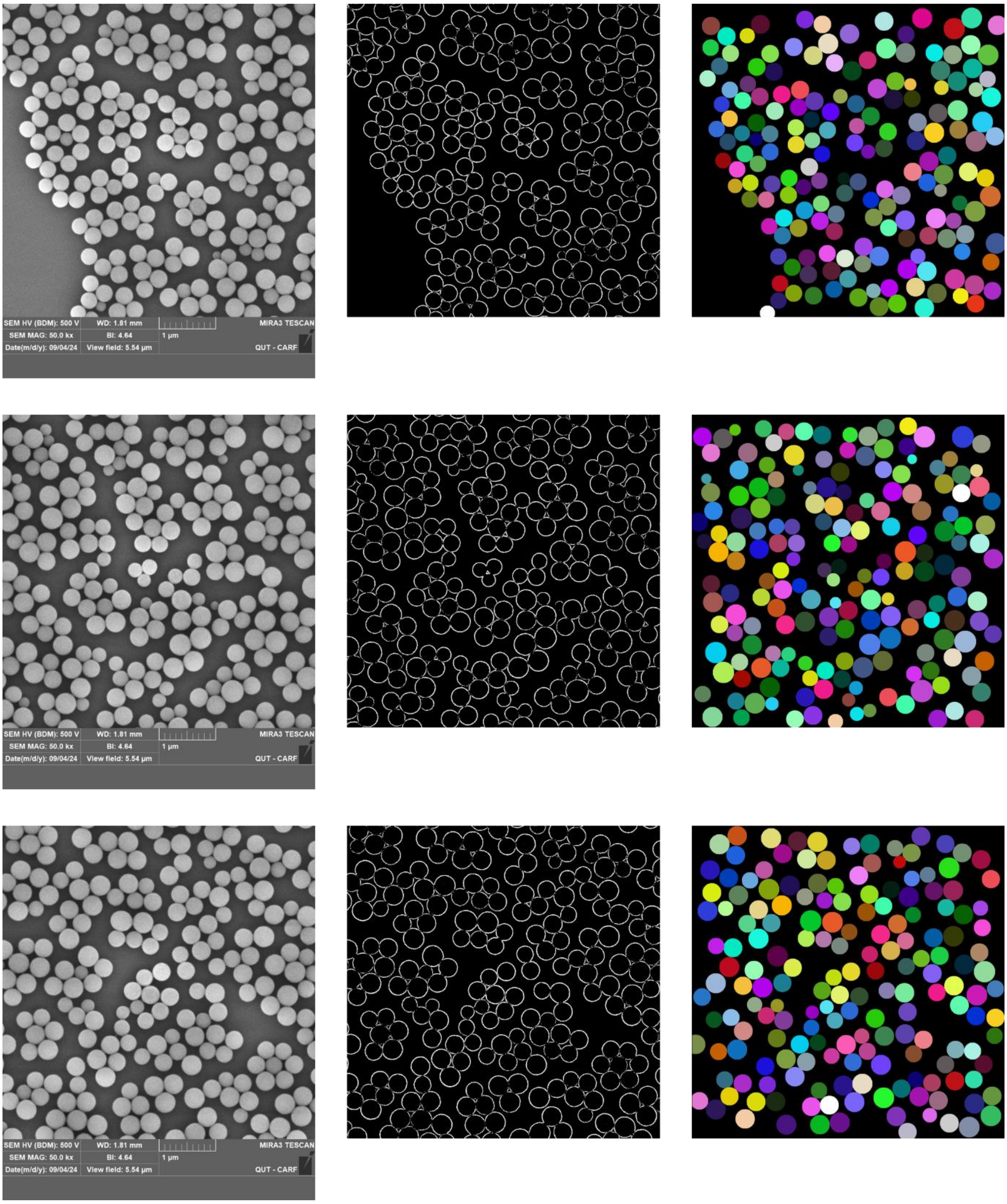

Figure S33. SEM size analysis via automated segmentation with edge detection and Circle Hough Transform for sample 83v.

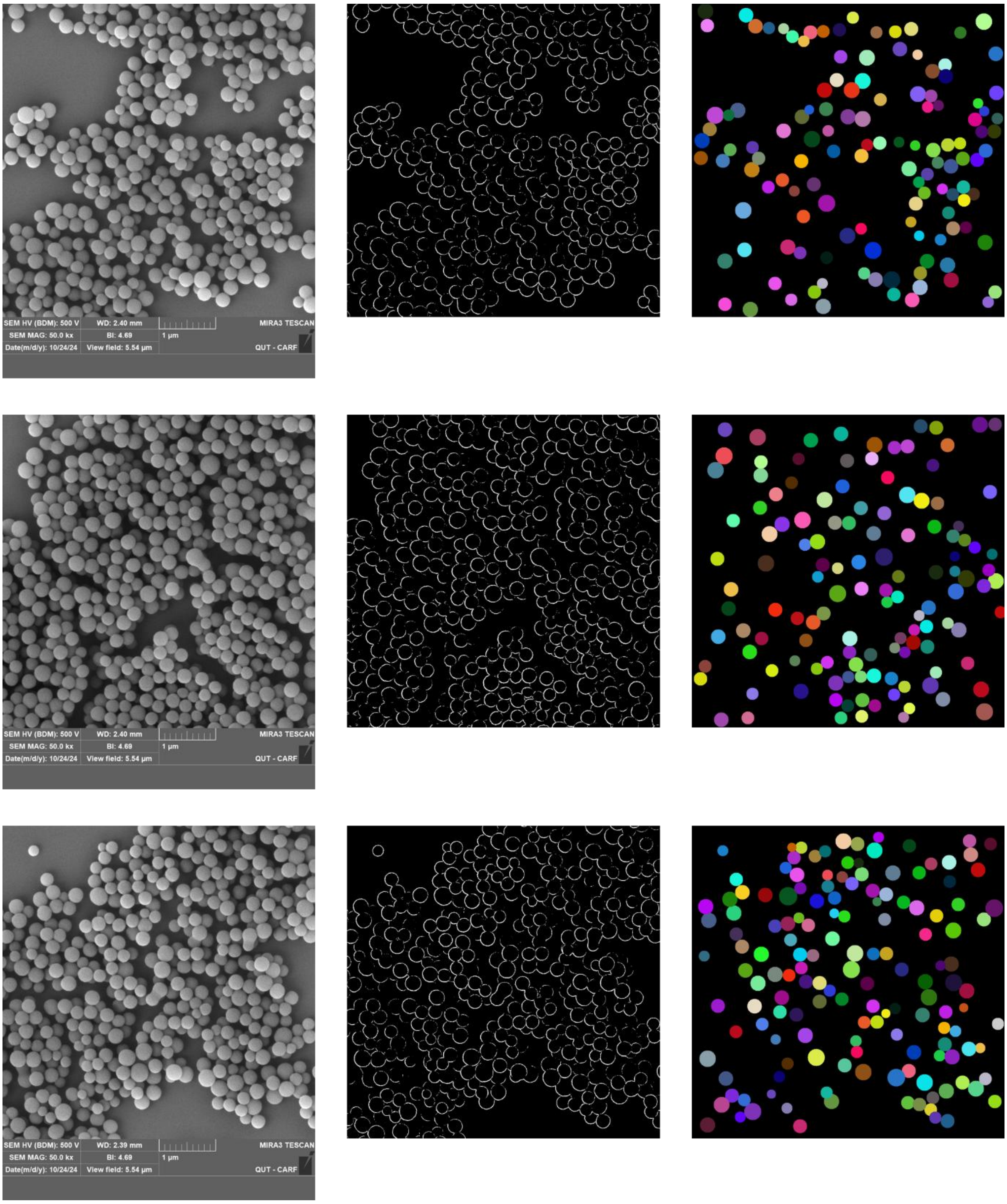

Figure S34. SEM size analysis via automated segmentation with edge detection and Circle Hough Transform for sample 83y.

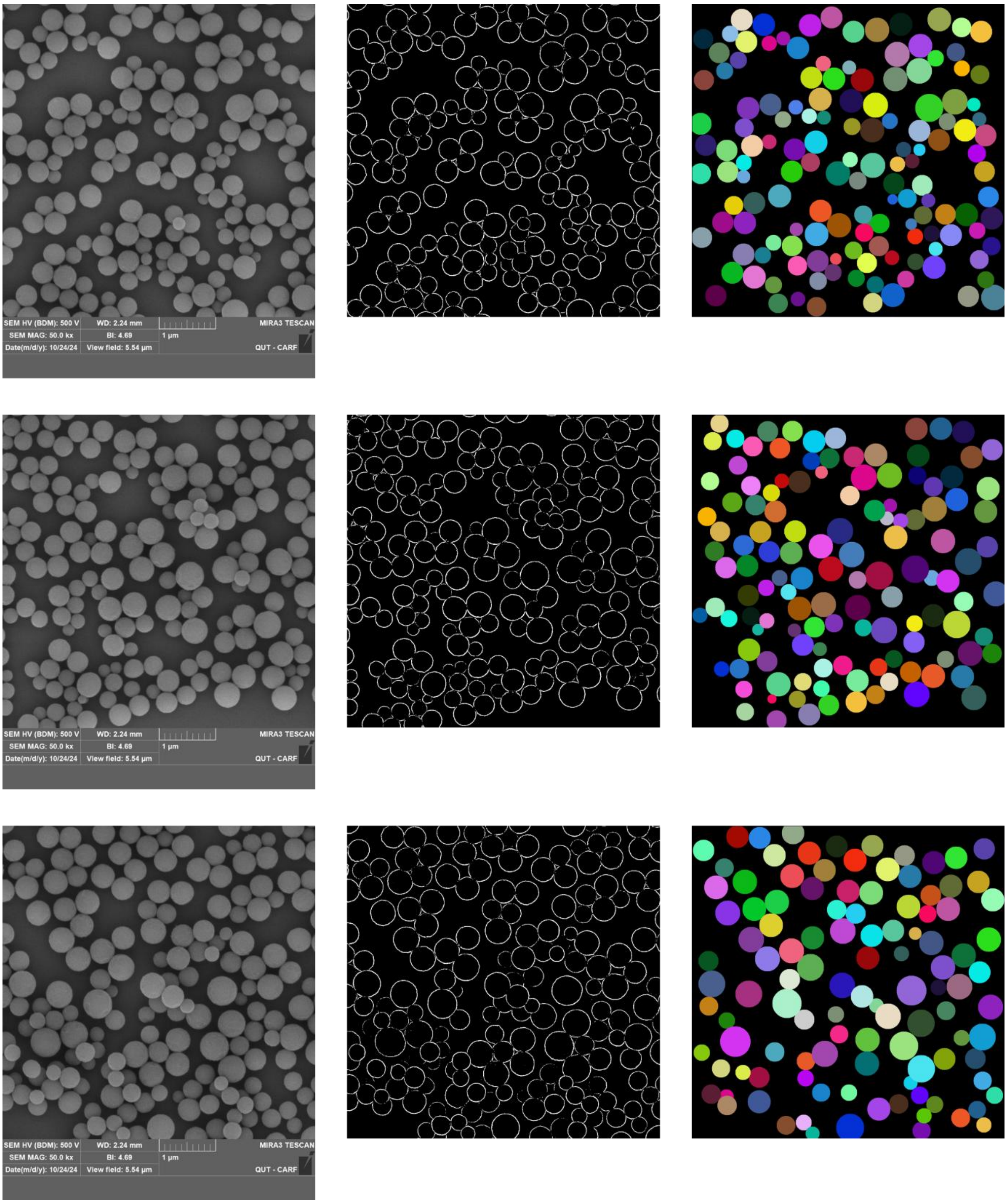

Figure S35. SEM size analysis via automated segmentation with edge detection and Circle Hough Transform for sample 83z.

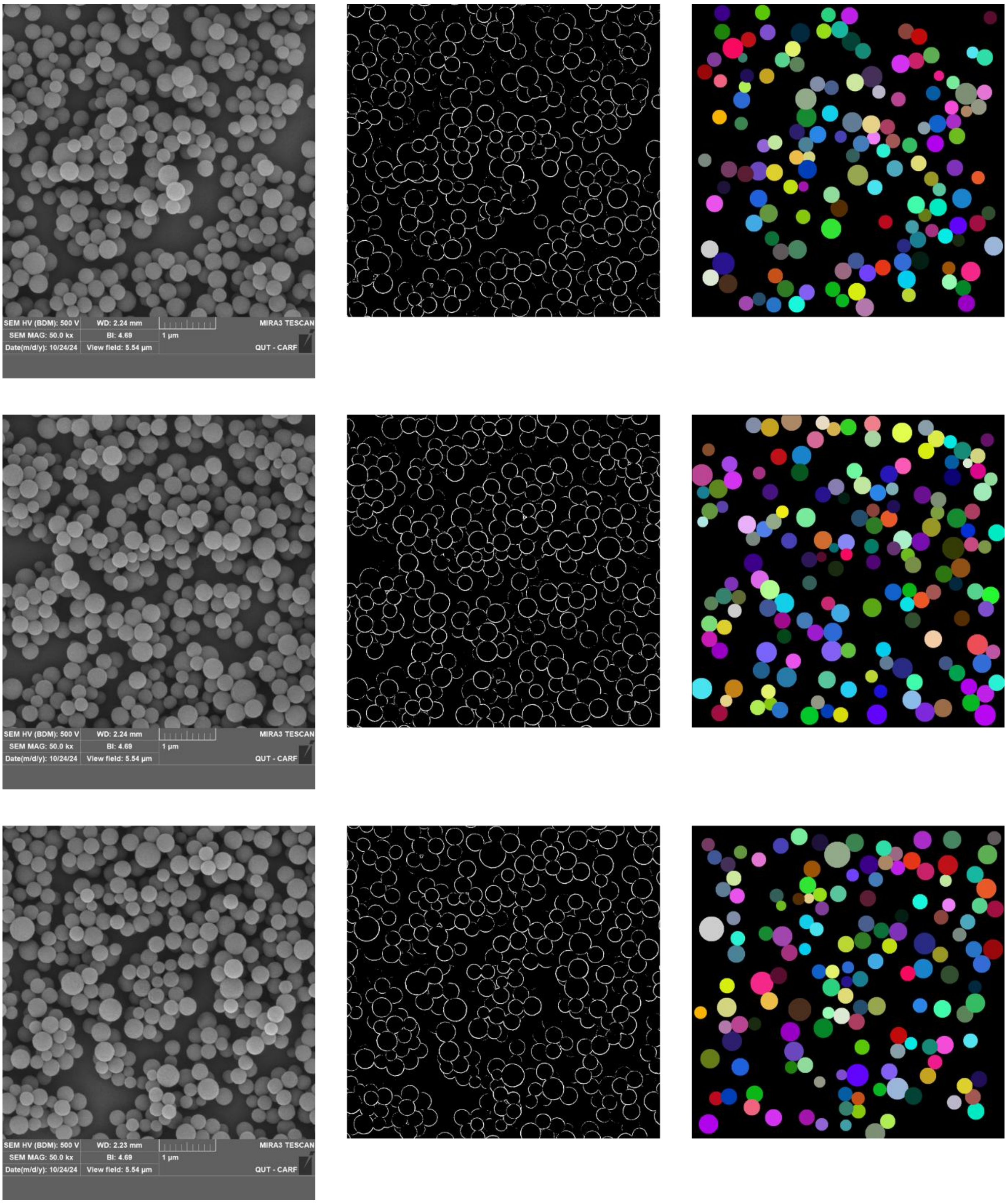

Figure S36. SEM size analysis via automated segmentation with edge detection and Circle Hough Transform for sample 83za.

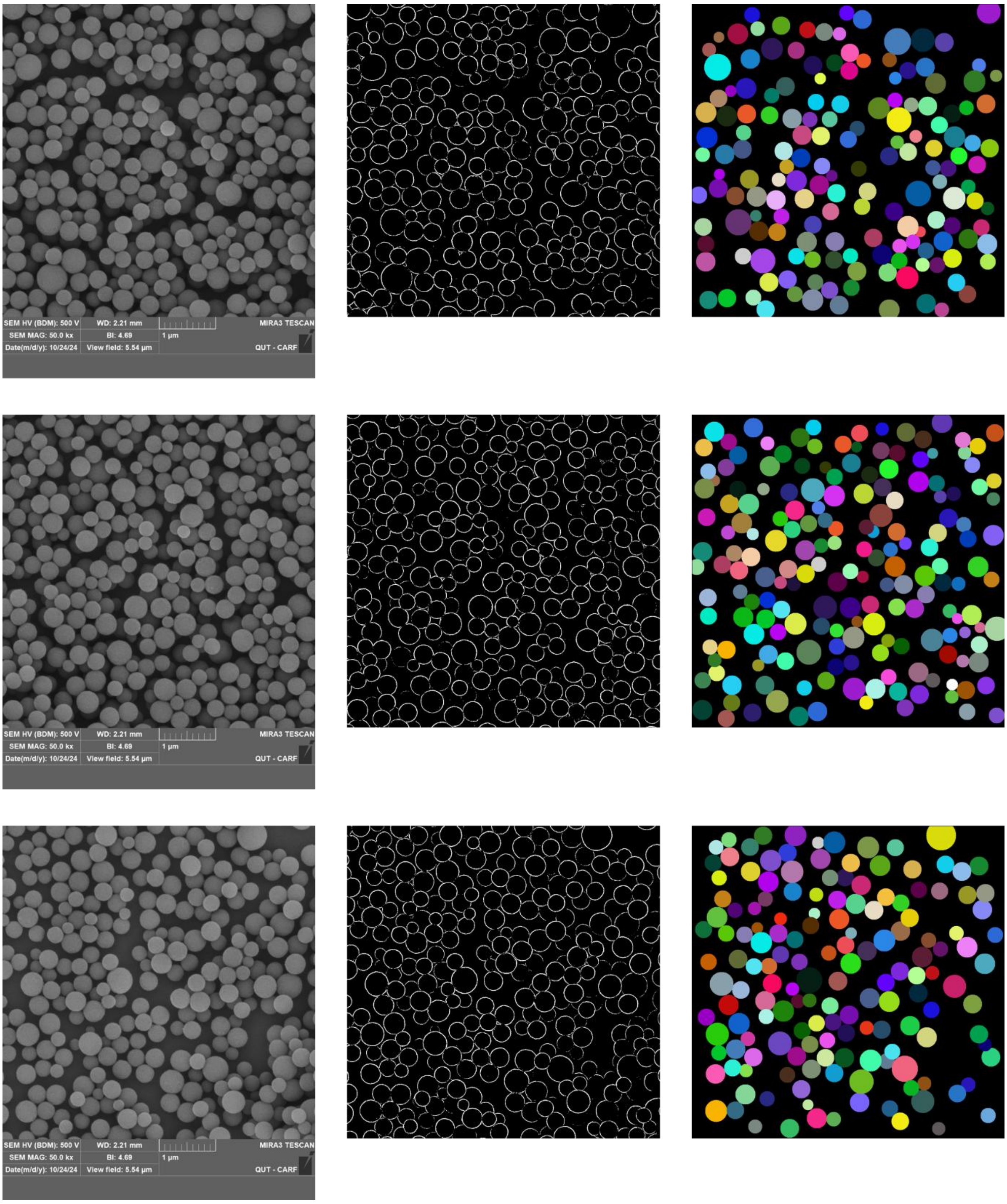

Figure S37. SEM size analysis via automated segmentation with edge detection and Circle Hough Transform for sample 83zb.

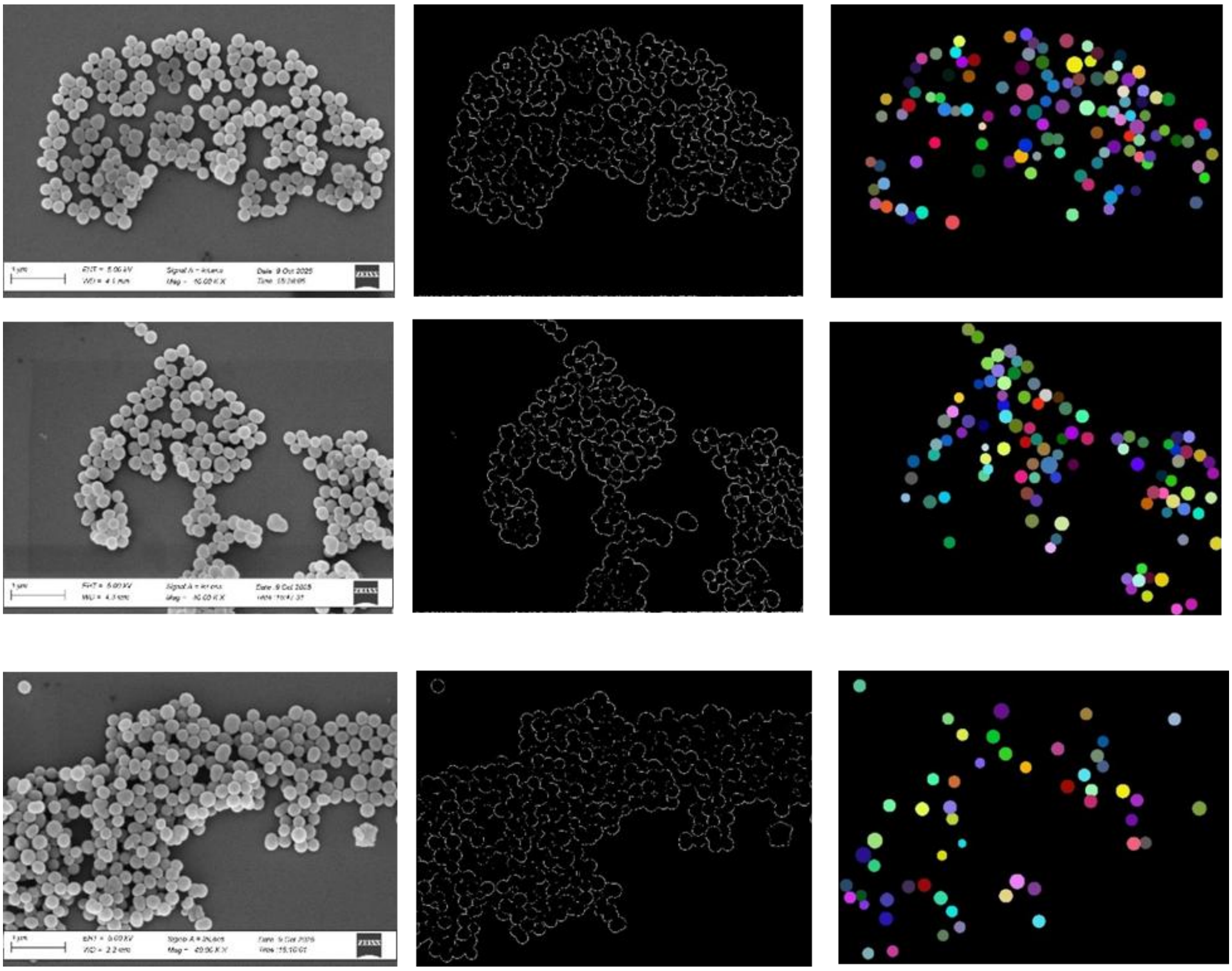

Figure S38. SEM size analysis via automated segmentation with edge detection and Circle Hough Transform for sample from experiment in Karlsruhe (Germany).

5.3.3. Histograms: 2 mm diameter tubing

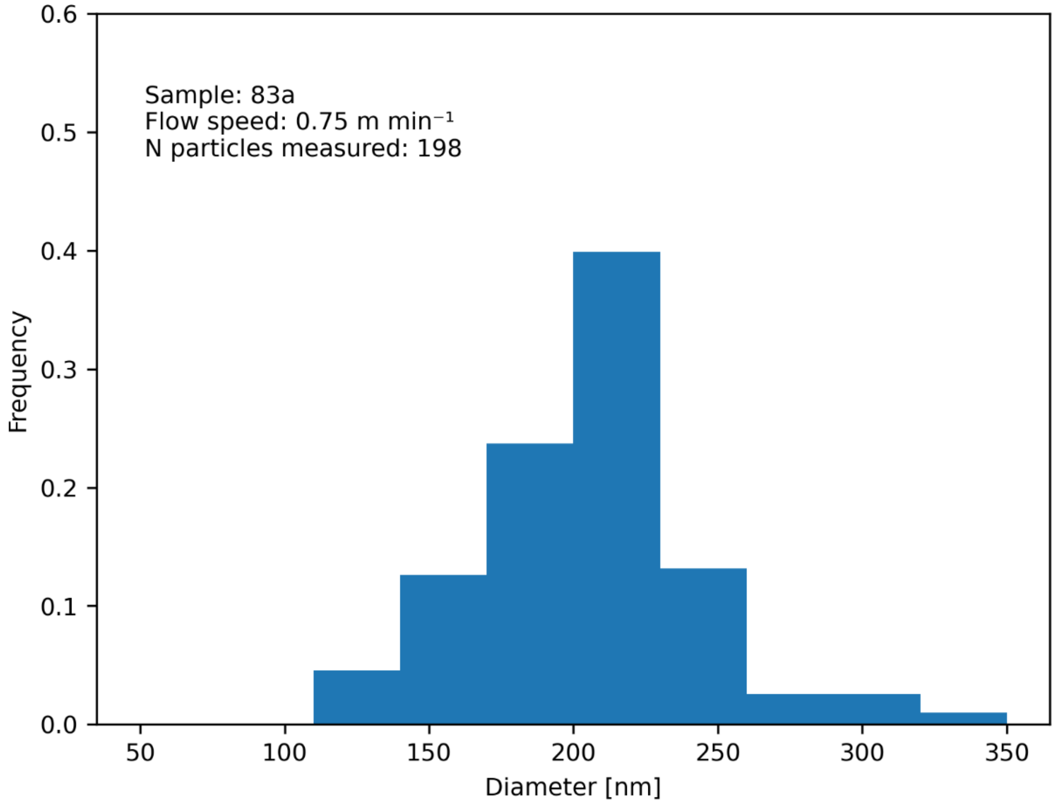

Figure S39. Size distribution from SEM analysis.

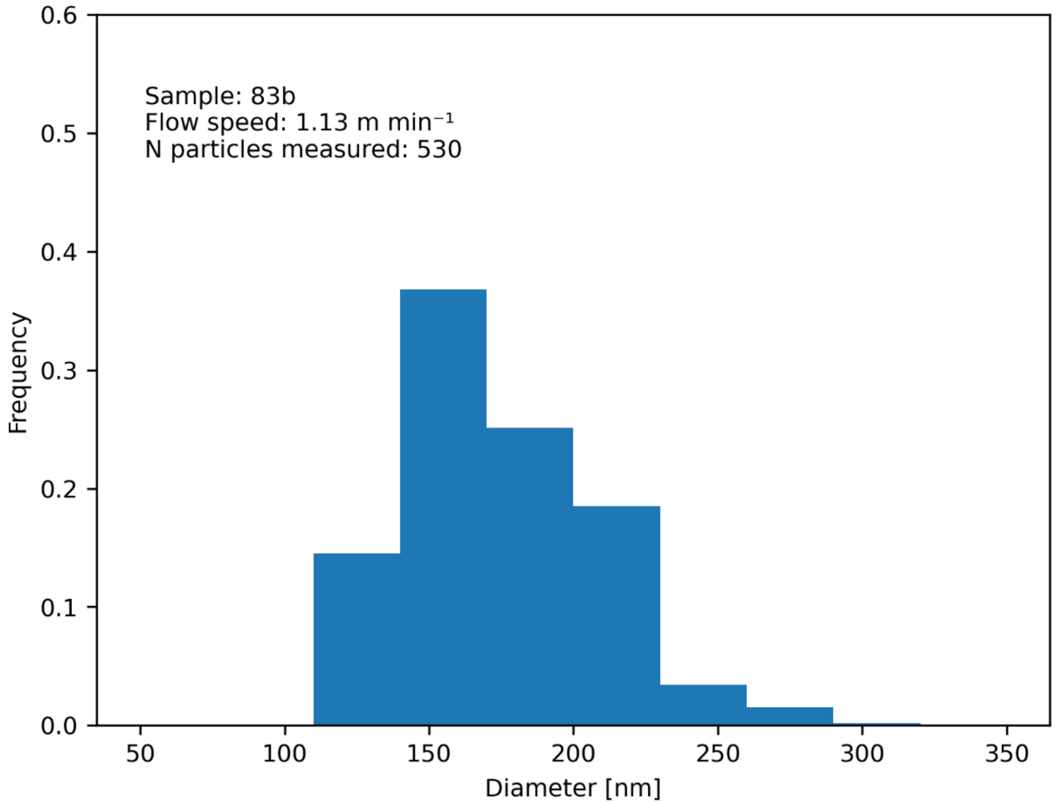

Figure S40. Size distribution from SEM analysis.

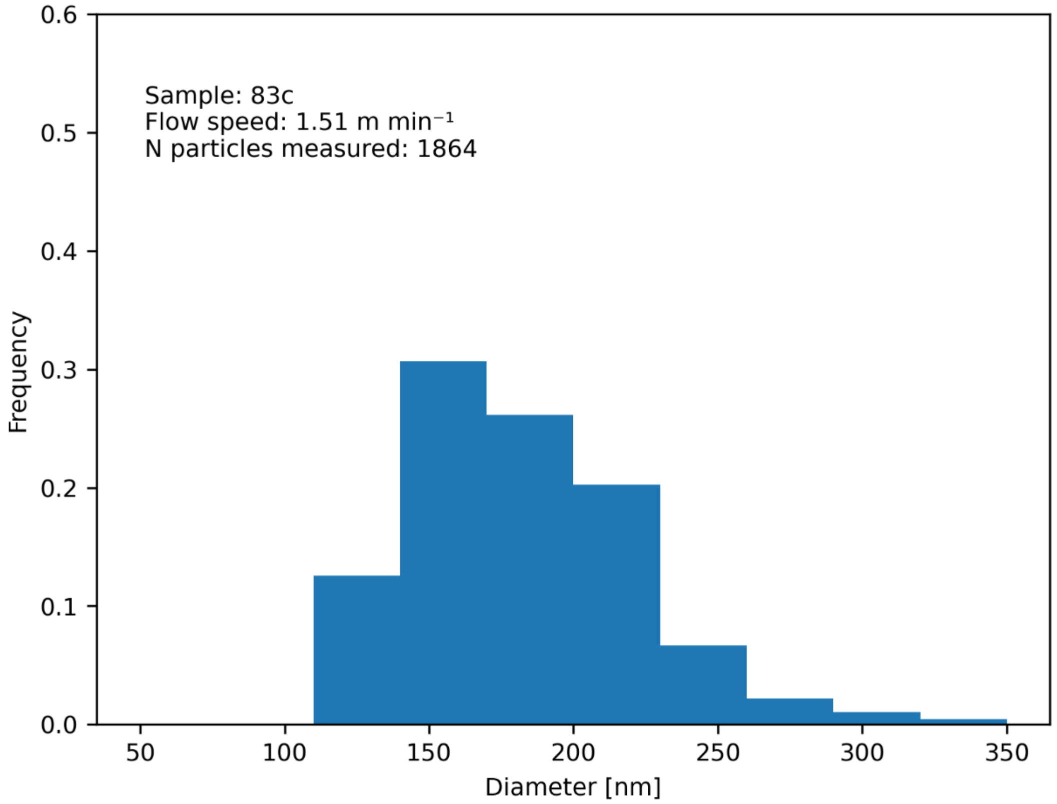

Figure S41. Size distribution from SEM analysis.

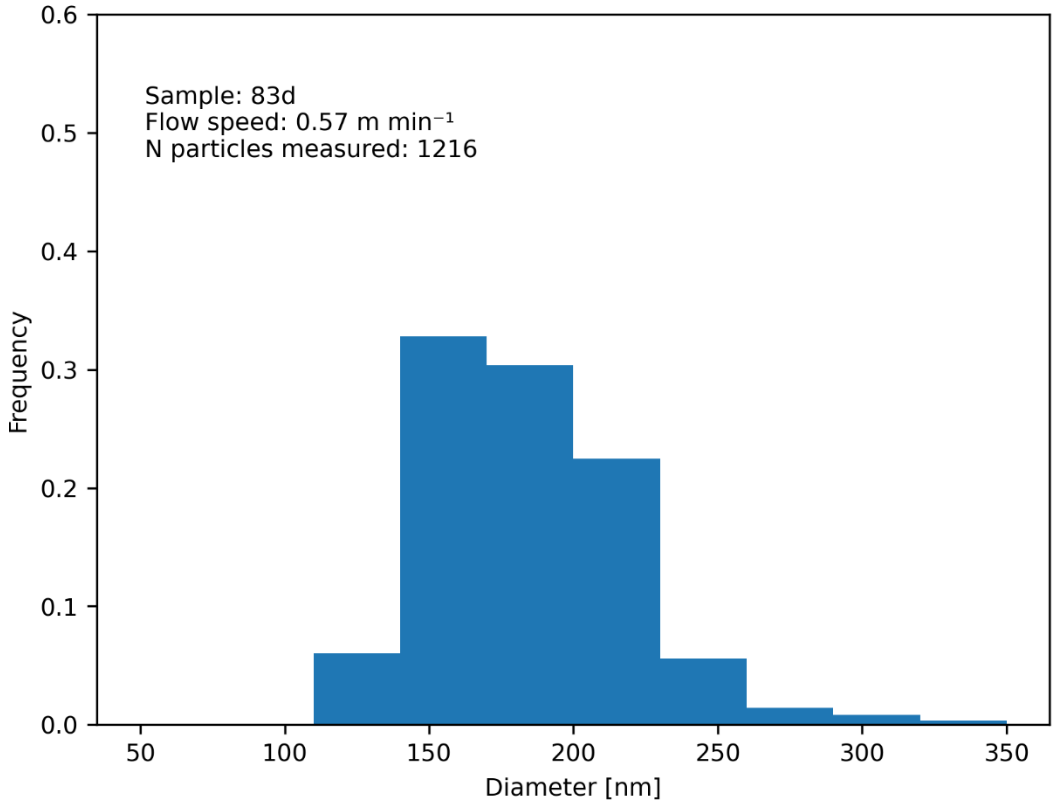

Figure S42. Size distribution from SEM analysis.

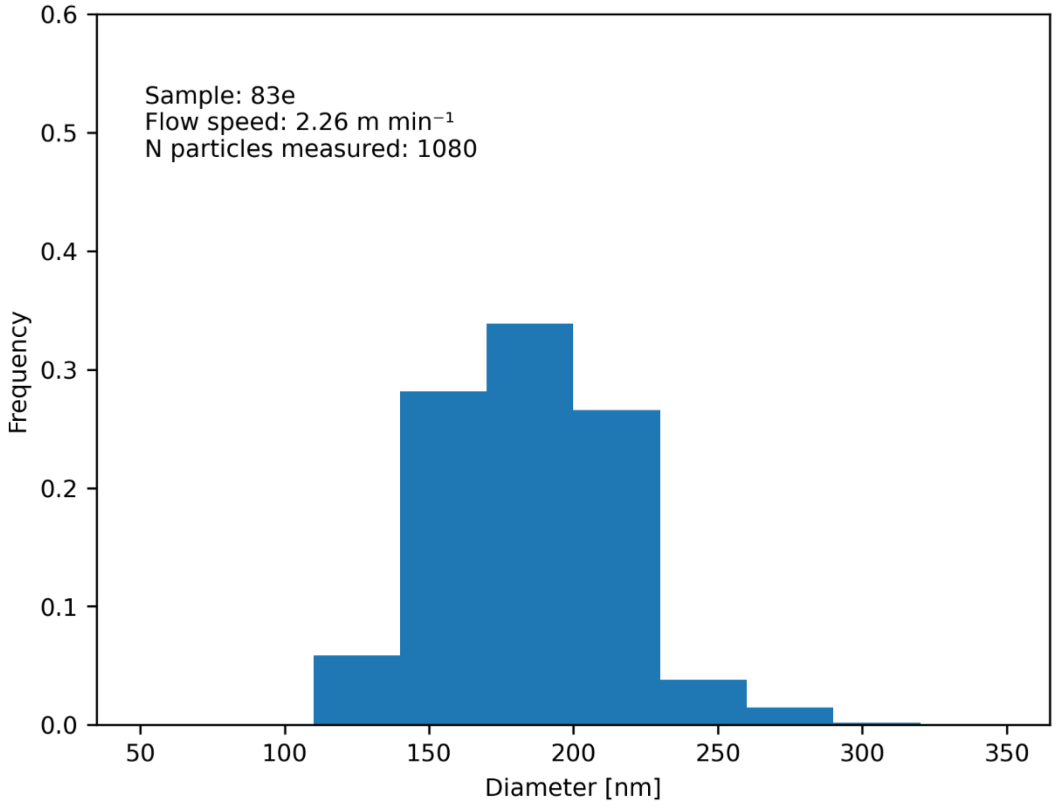

Figure S43. Size distribution from SEM analysis.

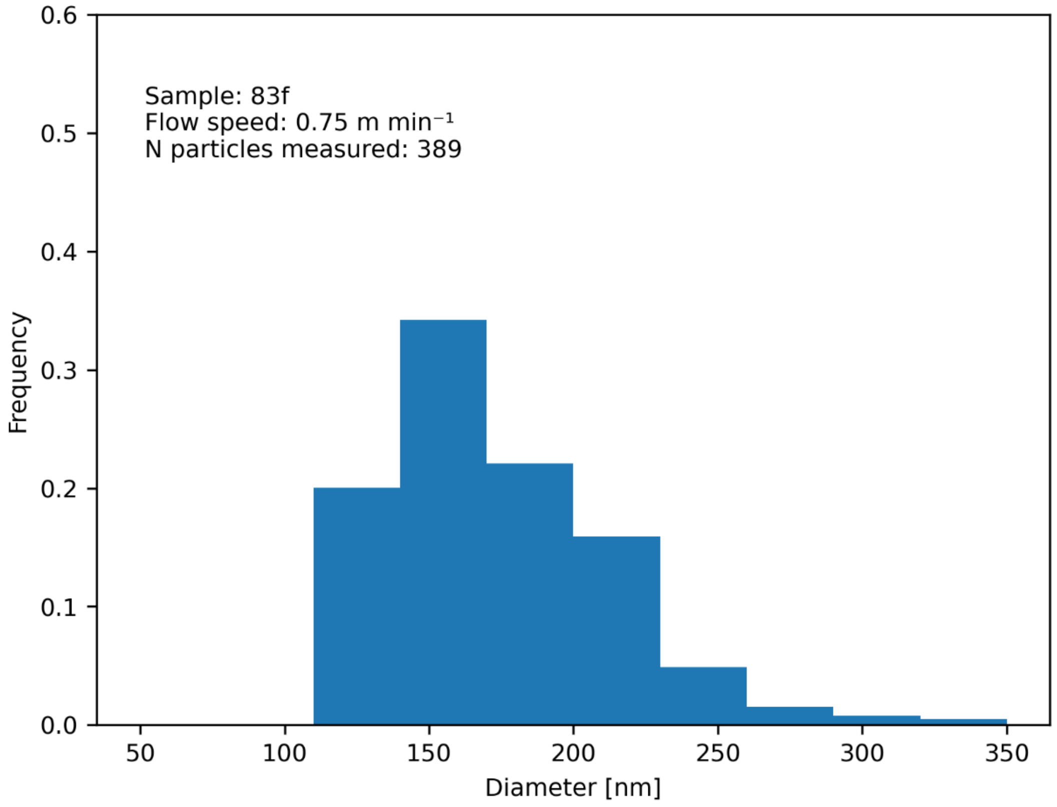

Figure S44. Size distribution from SEM analysis.

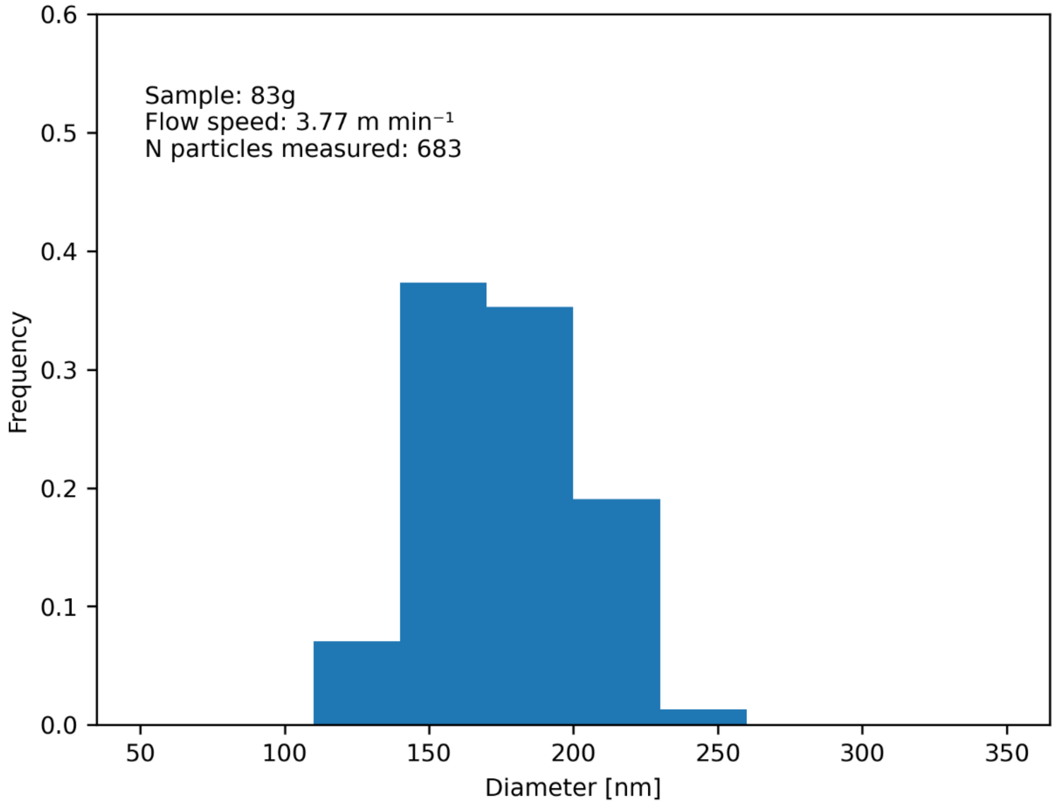

Figure S45. Size distribution from SEM analysis.

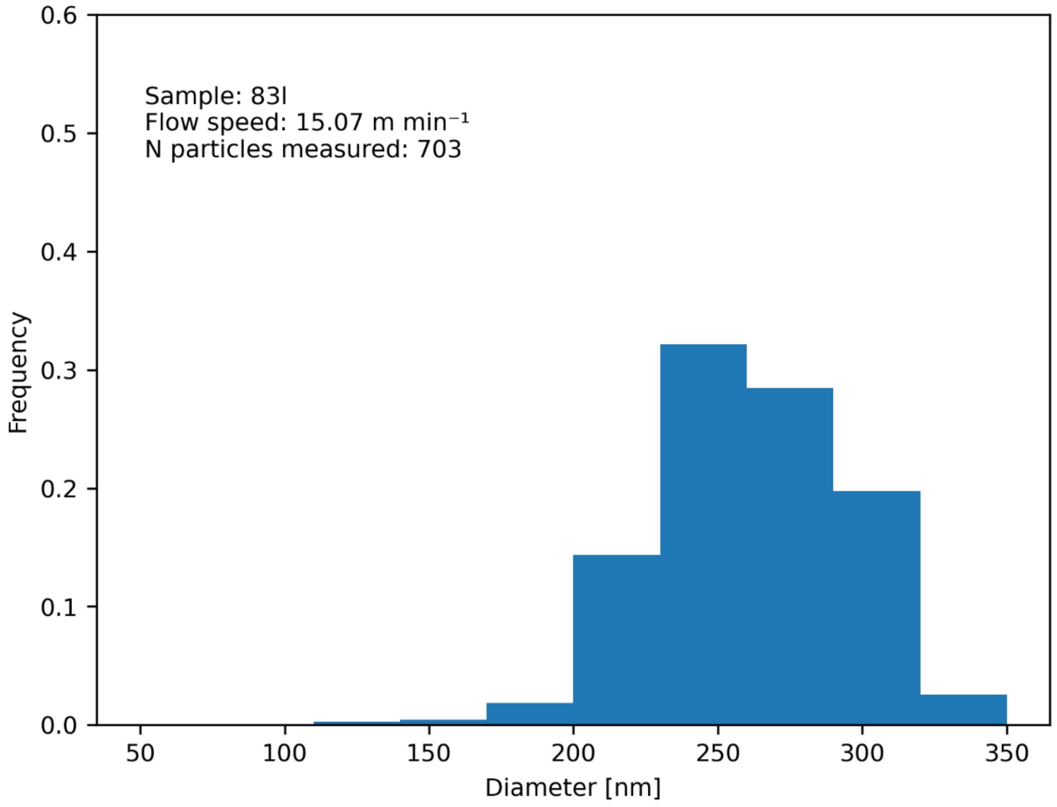

Figure S46. Size distribution from SEM analysis.

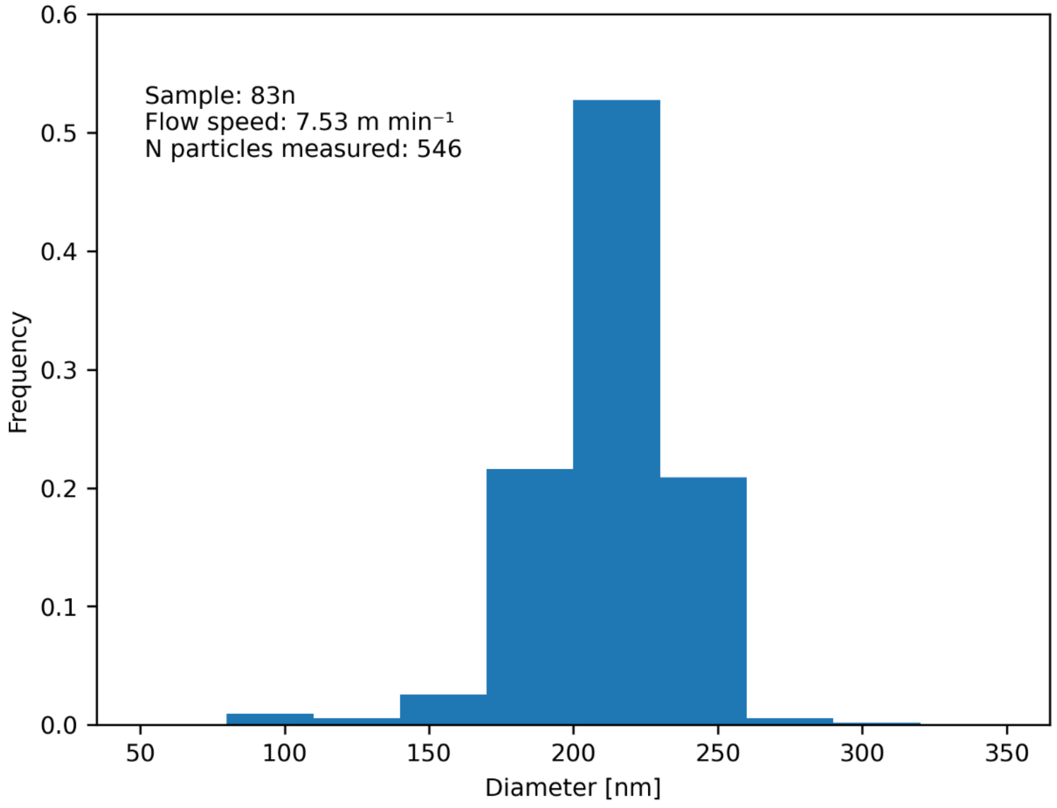

Figure S47. Size distribution from SEM analysis.

5.3.4. Histograms: 3 mm diameter tubing

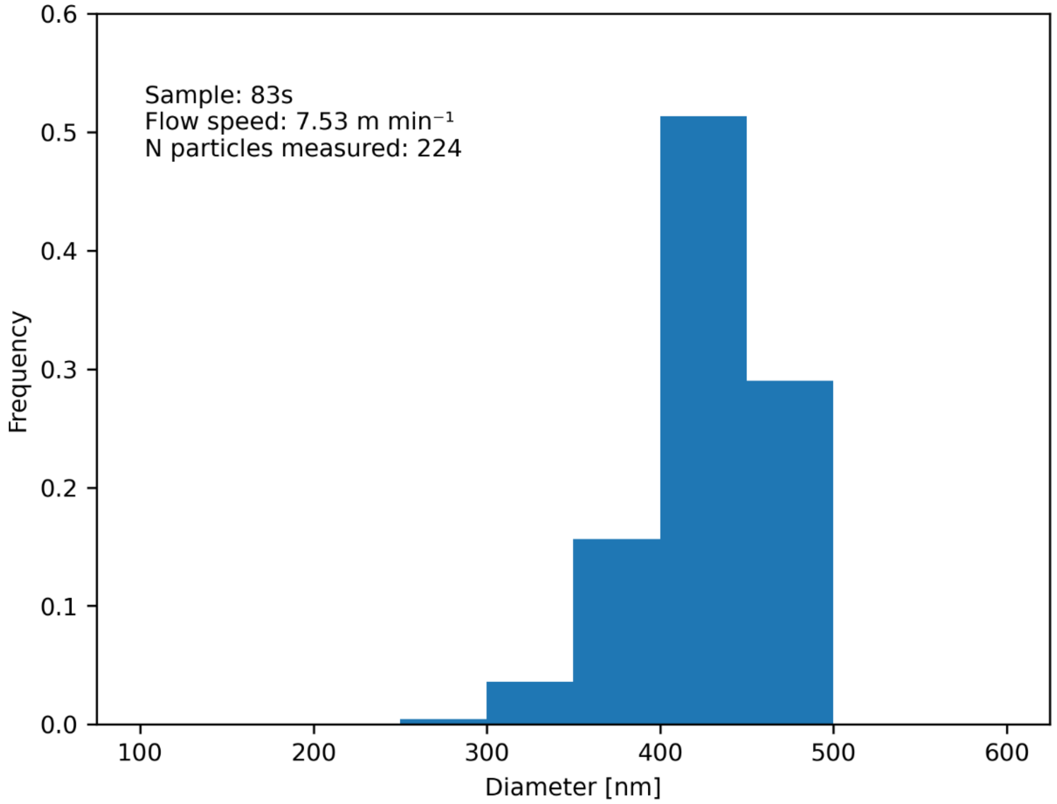

Figure S48. Size distribution from SEM analysis.

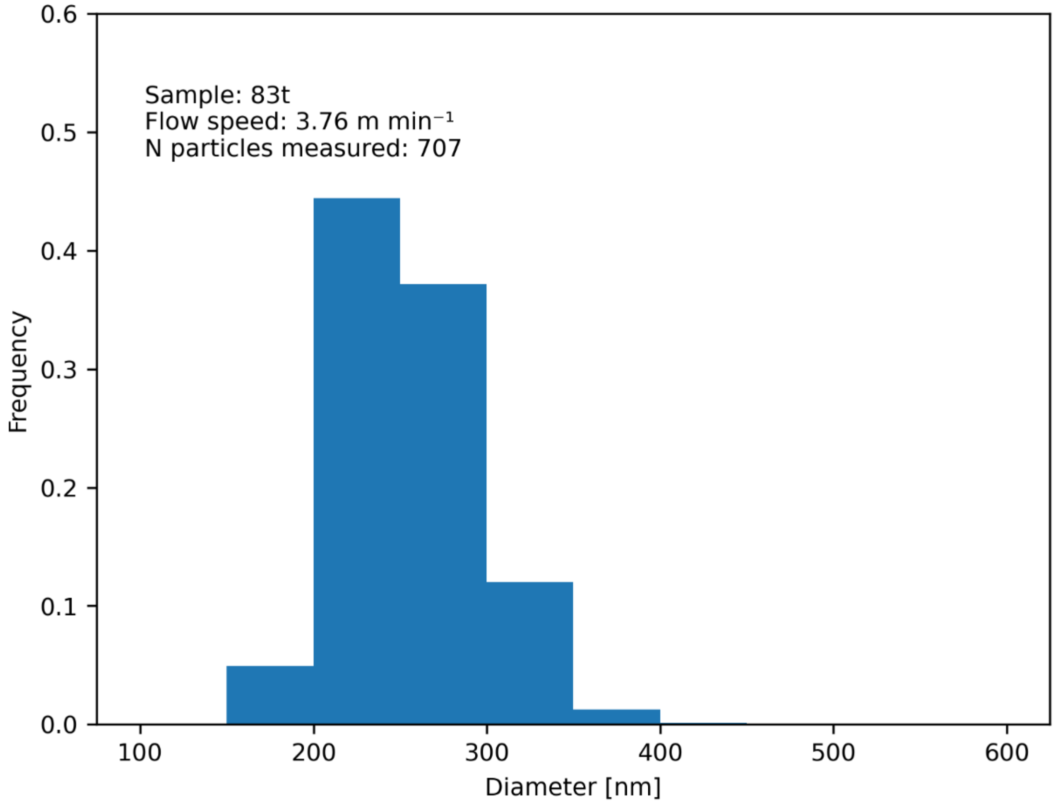

Figure S49. Size distribution from SEM analysis.

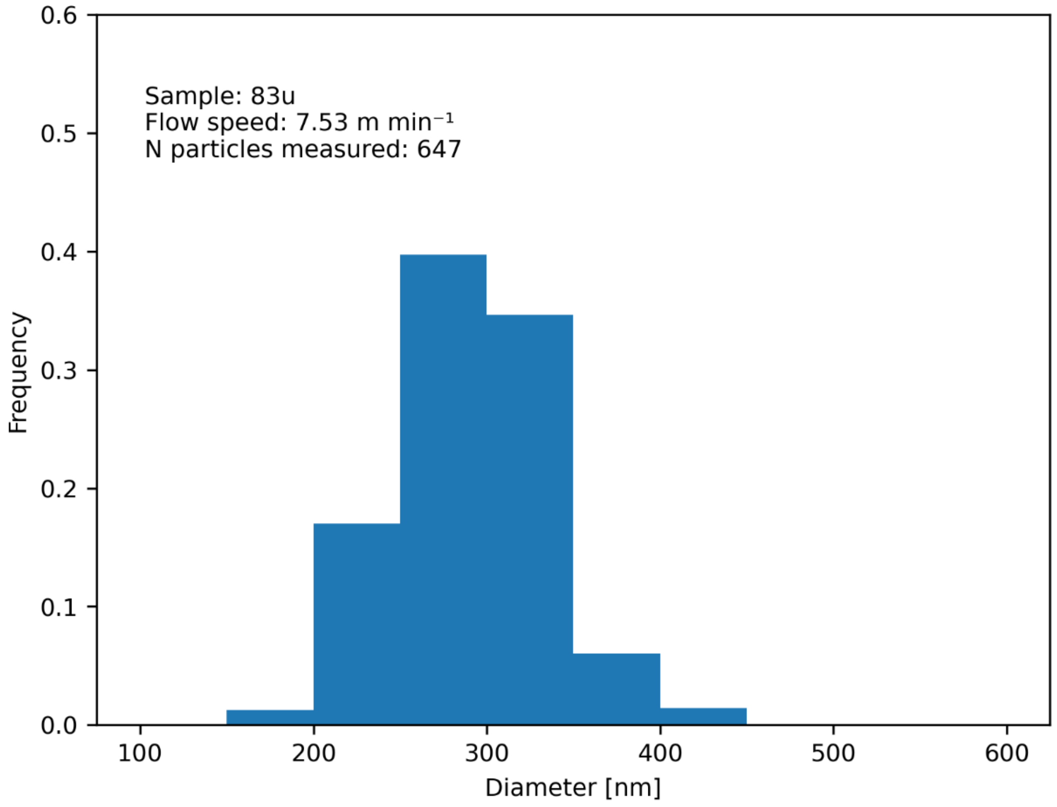

Figure S50. Size distribution from SEM analysis.

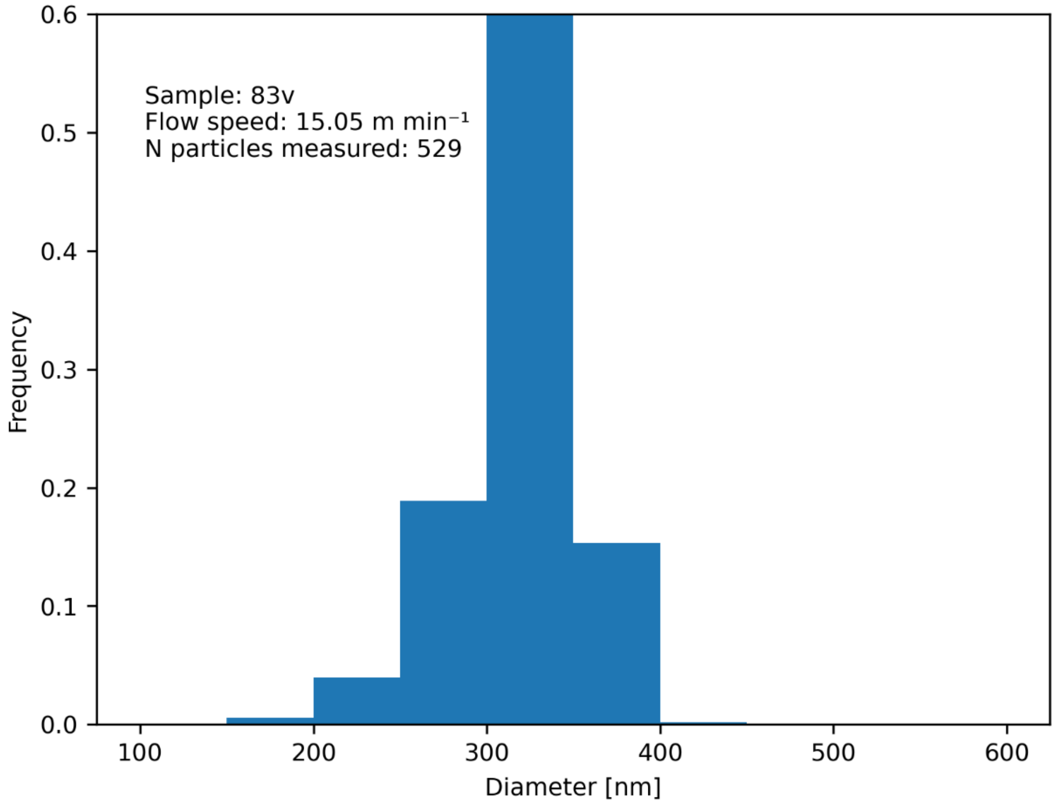

Figure S51. Size distribution from SEM analysis.

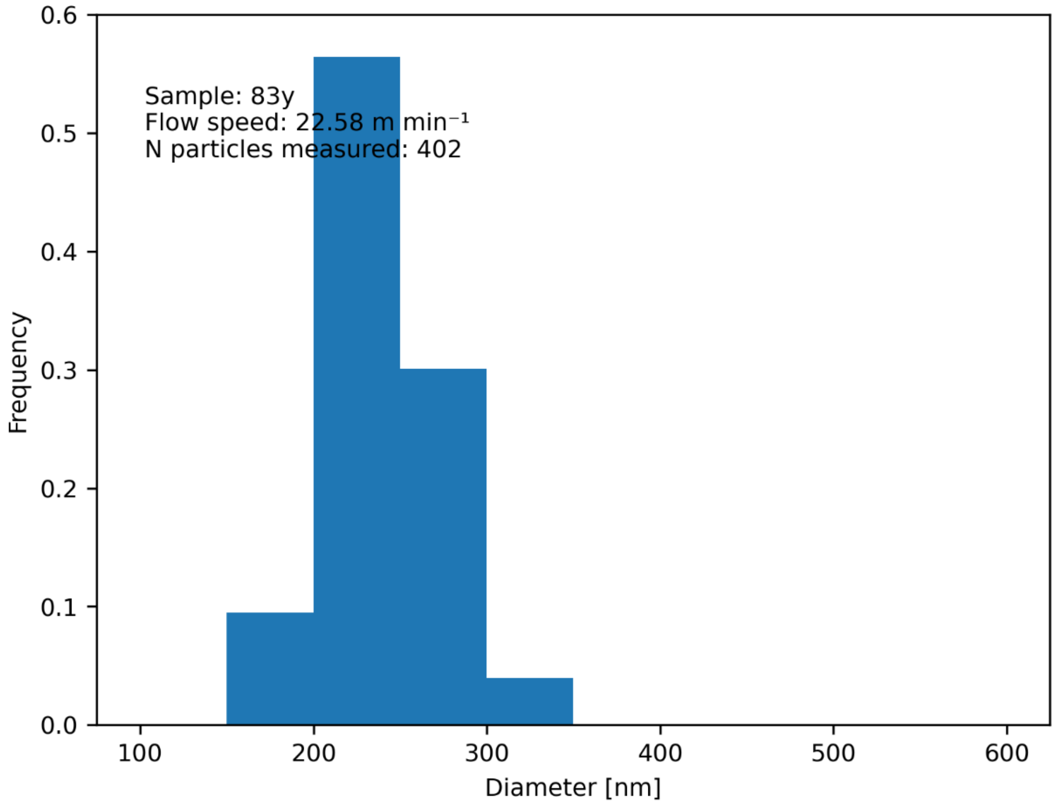

Figure S52. Size distribution from SEM analysis.

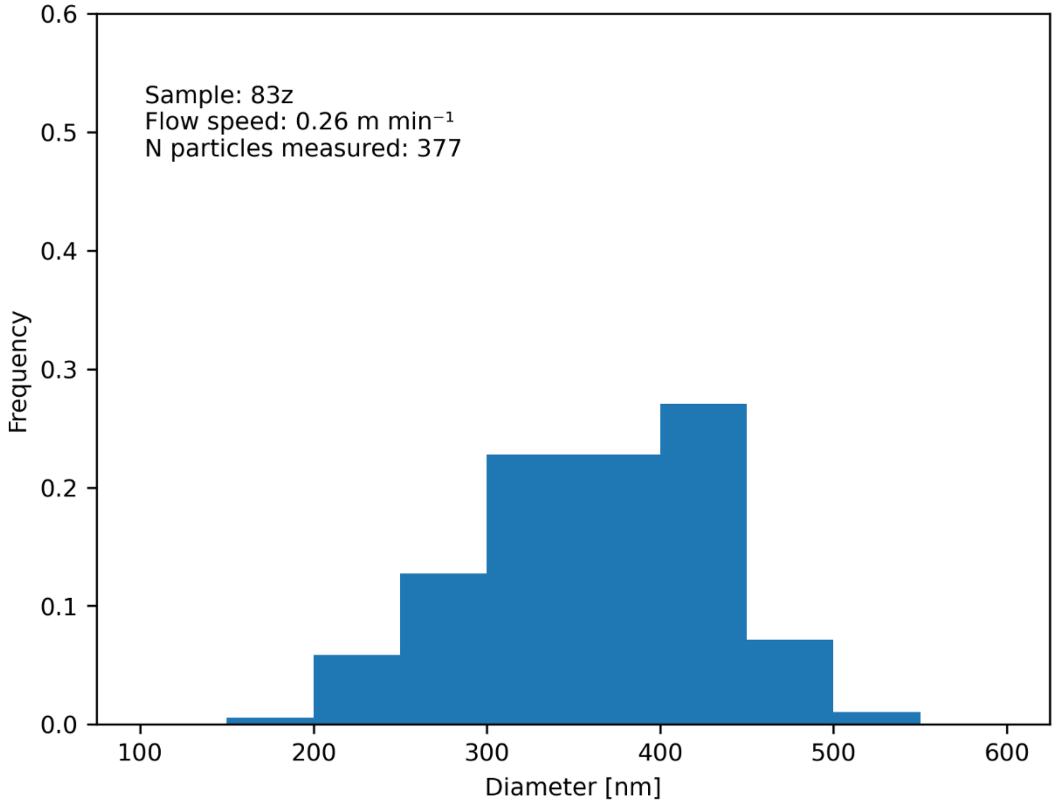

Figure S53. Size distribution from SEM analysis.

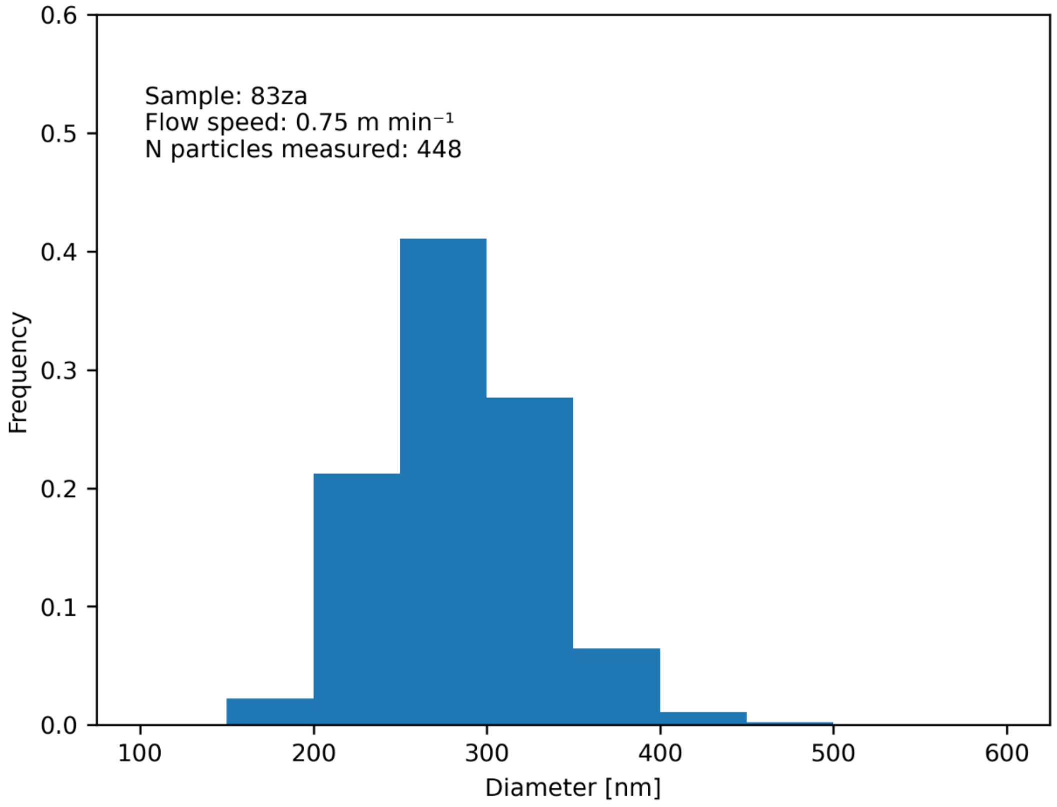

Figure S54. Size distribution from SEM analysis.

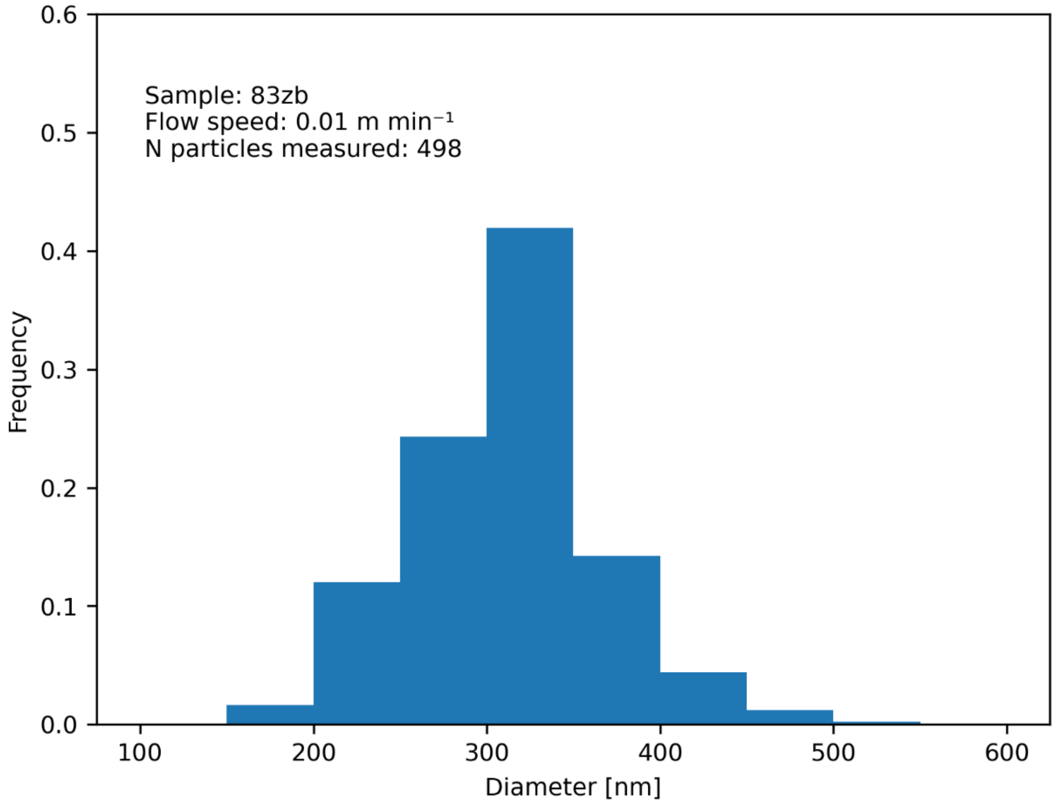

Figure S55. Size distribution from SEM analysis.

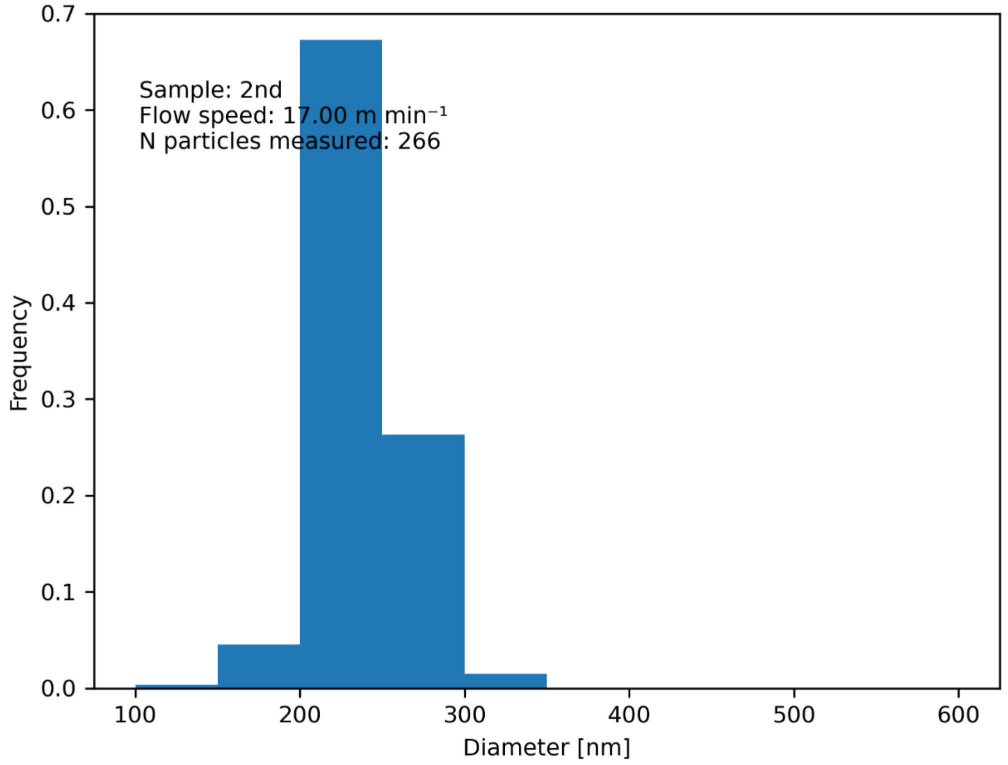

Figure S56. Size distribution from SEM analysis for experiments conducted in Karlsruhe (Germany).

5.4. SEC analysis

Oligomers with an apparent molecular weight >0.7 kDa begin to precipitate. This is evidenced by the highest oligomer peaks of each sample appearing before, or at this molecular weight (see chromatograms below).

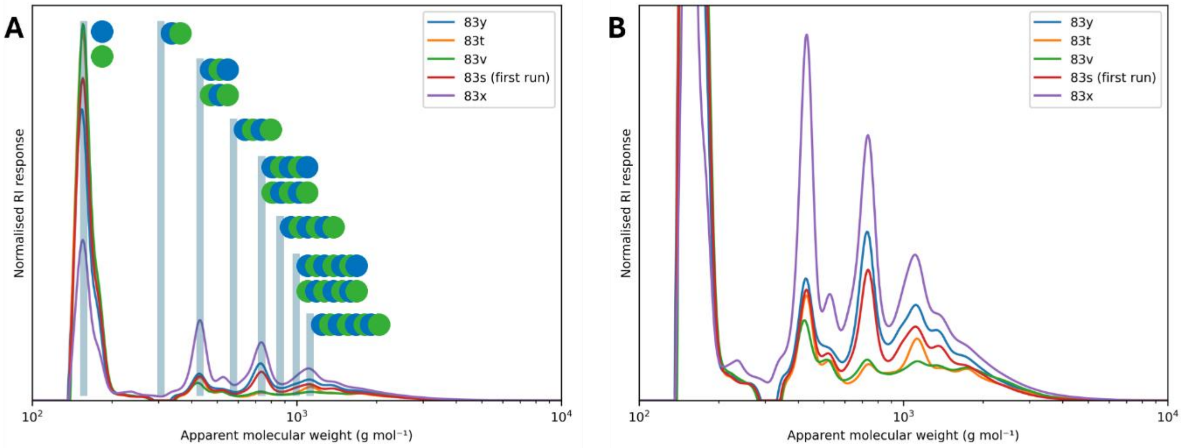

Figure S57. SEC chromatograms of the analysed samples. **A.** Full data with indicated oligomer peaks. **B.** zoom in.

6. Relative yield and yield per time calculation

The relative yield (Figure 3, main text) was calculated using the equation

$$y = \frac{m_{particle}}{m_{monomer}} = \frac{m_{particle}}{c \cdot V} \quad \text{(S2)},$$

where $m_{particle}$ is the mass of the isolated particles and $m_{monomer}$ is the used monomer mass, calculated from the monomer concentration, c, and the collected volume, V.

The yield per time (Figure 3, main text, dotted lines) was calculated as

$$\dot{y} = y \cdot Q \cdot c \quad \text{(S3)},$$

with the flow rate using

$$Q = \pi \cdot r^2 \cdot u \quad \text{(S4)},$$

where r is the inner tubing radius, and u the flow speed.

Combining Equation S3 and 4 yields the full expression for the yield per unit time

$$\dot{y} = \pi \cdot y \cdot r^2 \cdot u \cdot c \quad \text{(S5)}.$$

7. Derivation of the formula for yield per area and day of a solar chemical plant

Below, the derivation of Equation 1 (main text) for the yield per area and day of a hypothetical solar chemical plant is described in detail. The calculations are also available as python script in the Jupyter Notebook *SI_global_solar_photochemical_potential.ipynb* as Supporting Information together with the anaconda environment *SI_worldmaps.txt* to execute it.

The starting point for the calculations is the formulation for the yield per area and day

$$Y = \frac{\dot{y} \cdot t_{\mathrm{reac}}}{A} \quad \text{(S6)}.$$

Thereby $\dot{y}$ is the yield per unit time, t_{reac} the runtime of the reaction per day, and A the area requirement of the plant as

$$A = l \cdot w \cdot 2.5 \quad \text{(S7)},$$

where l is the tube length and w the lateral space requirement, for which we chose 1 cm as centre-to-centre distance between two adjacent tubes. The factor of 2.5 accounts for the space requirement for supporting structures and to avoid shadowing of neighbouring panels. The value of 2.5 is based on the typical space requirement for solar power plants, where an area of two to three times the area of the solar modules is needed.[8]

The tubing length necessary to complete the reaction is

$$l = u \cdot t_{\mathrm{comp}} \cdot 1.1 \quad \text{(S8)}$$

as a function of the flow speed, u, and the time to reaction completion, t_{comp}. The additional factor of 1.1 accounts for a 10 % extra tubing length as safety margin for the completion of the reaction. The time to completion is given as

$$t_{\mathrm{comp}} = \frac{T}{UVI} \quad \text{(S9)},$$

which is determined by the UV index normalised exposure time to completion T, and the UV index, UVI. The UVI varies with time and date. The used UV index dataset[9] provides the monthly average

UV index, i.e., the UV index averaged over every day and night of the month. To calculate the UV index over the course of a day, we approximated it by a cosine function, as follows:

$$UVI(t) = \frac{1}{2} \cdot UVI_{peak} \cdot \left[\cos\left(\frac{\pi t}{\frac{1}{2} \cdot t_{sun}}\right) + 1\right] \qquad \text{(S10).}$$

We defined this function symmetrically around the zenith ($t = 0$), when the UV index peaks with UVI_{peak}(see Figure S58). t_{sun} is the daylight hours per day and $\frac{1}{2} \cdot (-t_{sun})$ and $\frac{1}{2} \cdot t_{sun}$. corresponds to sunrise and sunset, respectively. Outside of this range, we define $UVI(t)$ as 0. The comparison of our cosine function for $t_{sun} = 12$ h and the predicted UV index during autumnal equinox in Brisbane, Australia, (20st March 2024)[6] in Figure S58 show good agreement.

For the calculation of the daylight hours per day t_{sun}, we used the difference between the time for sunrise and sunset, calculated with the python library *SunTime*.[10] All monthly average daylight hours are plotted in Section S8.1.

To calculate the peak UV index from the averaged UV index data, we used the following expression for the average UV index:

$$\overline{UVI} = \frac{\int_{-0.5 \cdot t_{sun}}^{0.5 \cdot t_{sun}} UVI(t)\, \mathrm{d}t}{t_{sun}} = \frac{\left[UVI_{peak}\left[\frac{t_{sun} \cdot \sin\left(\frac{2\pi t}{t_{sun}}\right)}{4\pi} + \frac{t}{2}\right] + constant\right]_{-0.5 \cdot t_{sun}}^{0.5 \cdot t_{sun}}}{t_{sun}} = \frac{UVI_{peak}}{2} \qquad \text{(S11).}$$

Therefore, the peak UV index can simply be approximated by

$$UVI_{peak} = 2 \cdot \overline{UVI} \qquad \text{(S12).}$$

Combining Equation S6 with the Equations S5, and S7 to S9, we obtain Equation 1 from the main text, which is the full expression for the yield per area and day of the solar plant using our reactor design. From a construction point of view, the solar plant must be constructed with a fixed tubing length. The length of the tubing is (at a fixed flow rate) determined by the UV index (see Equation S8, and S9). To achieve the highest possible yield throughout the year, the UV index at the start of the reaction, $UVI(t_o)$ must be chosen optimally, as it determines the tubing length of the reactor. Thereby, t_o is the time of the reaction start, which varies throughout the year, as the daylight hours per day t_{sun} and UVI_{peak} are seasonal. As the function for the time dependent UV index (Equation S10) is symmetric to $t = 0$ (zenith), the reaction time - i.e., the time until the UV index is too low again to drive the reaction to completion - is linked to the starting time of the reaction by

$$t_{reac}(t_0) = 2 \cdot |t_o| \qquad \text{(S13).}$$

Equation 1 (main text) was then optimised for the UV index at reaction start, $UVI(t_o)$ that yielded the highest possible yields throughout the year. The corresponding time of reaction start, t_o was calculated by solving Equation S10 for t, and the related time of reaction, $t_{reac}(t_0)$ of Equation S13.

As the peak UV index, and the daylight hours vary significantly throughout the seasons, the optimisation of Equation 1 (main text) was performed based on monthly data. The monthly average daylight hours per day and monthly peak UV-indices are plotted in Section S8.1, and S8.2, respectively. The optimal UV index at reaction start is plotted in Section S8.3, and the monthly yields per area and day in Section S8.4.

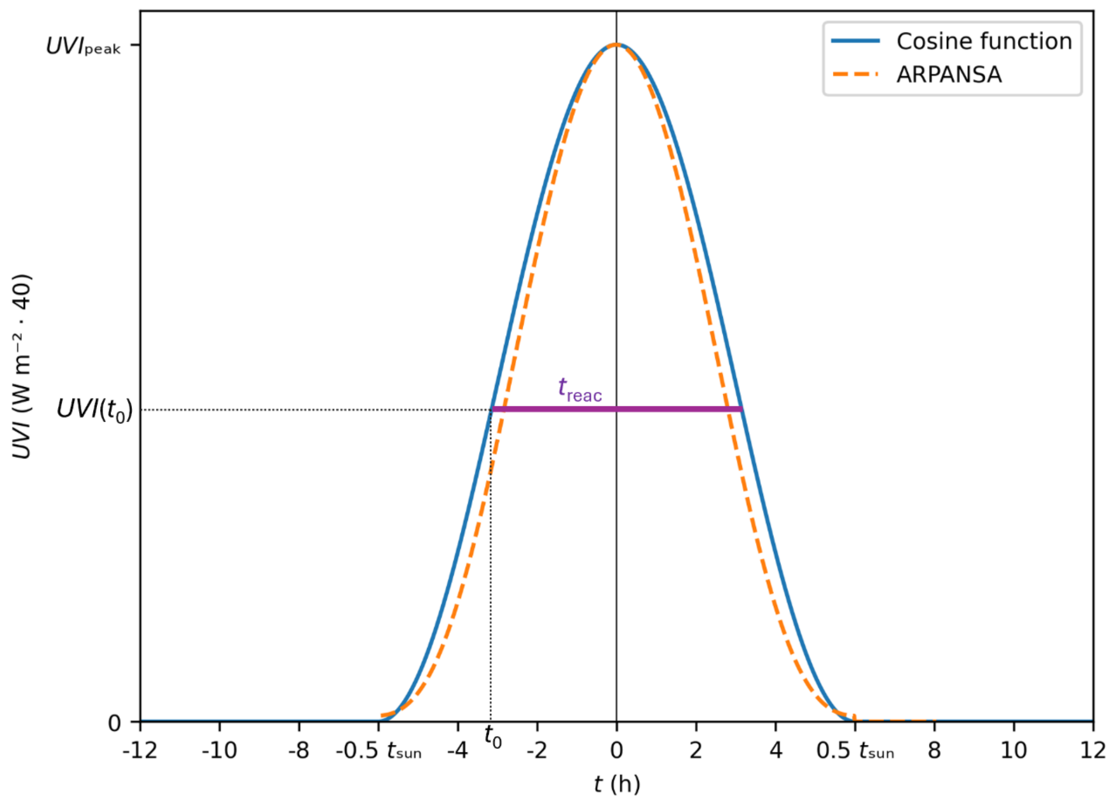

Figure S58. Daytime dependent UV index from Equation S9 for $t_{sun} = 12\ \mathrm{h}$ (blue) and comparison to ideal UV index data during equinox in Brisbane, Australia (orange, dashed).[6]

8. Additional calculations and global data plots

All calculations described in Section S7 and the presented plots of this section were obtained with the python script provided as Supporting Information *SI_global_solar_photochemical_potential.ipynb*, and the corresponding anaconda environment *SI_worldmaps.txt*.

8.1. Monthly average daylight hours per day

In the following, the monthly average daylight hours between sunrise and sunset per day are plotted, calculated using the python library *SunTime*[10] as the time difference of sunrise and sunset. In the white areas, no sunset or sunrise occurs, due to perpetual daylight or darkness, depending on hemisphere. As a result of the unavailable data, the UV index cannot be calculated for these regions, leading to zero yield in Figure 6 (main text). However, these areas are generally of low peak UV index and inconvenient climate, and are therefore of less interest for the solar production of nanoparticles.

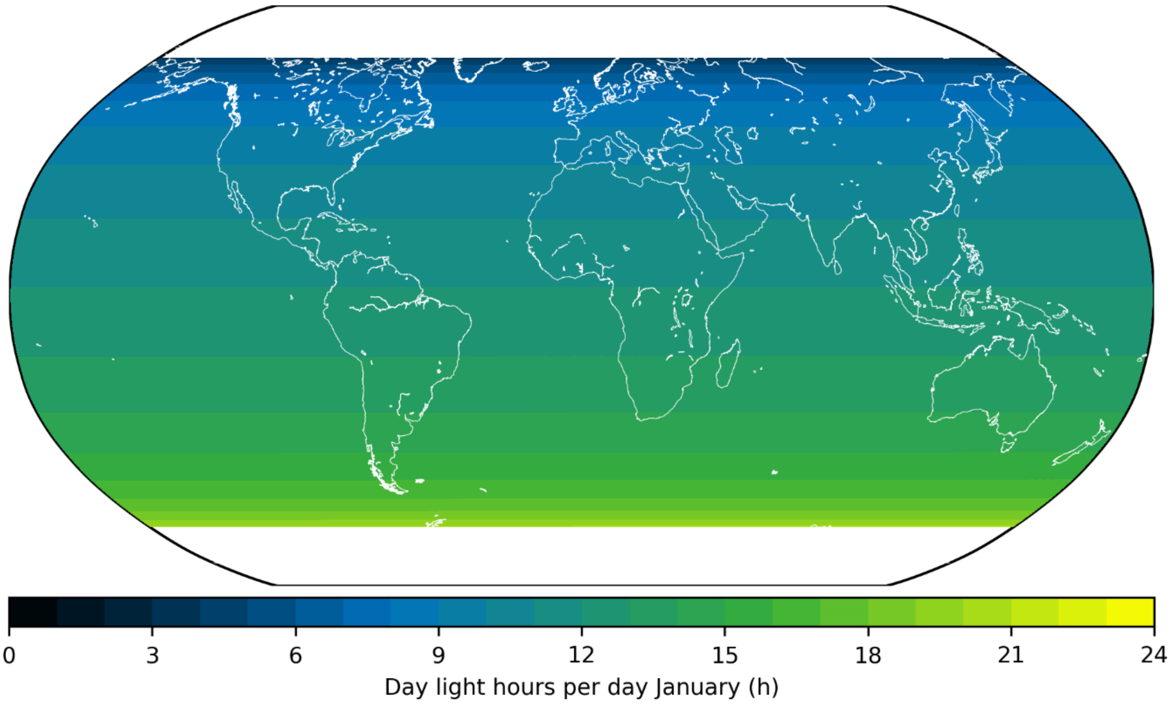

Figure S59. Monthly average daylight hours per day.

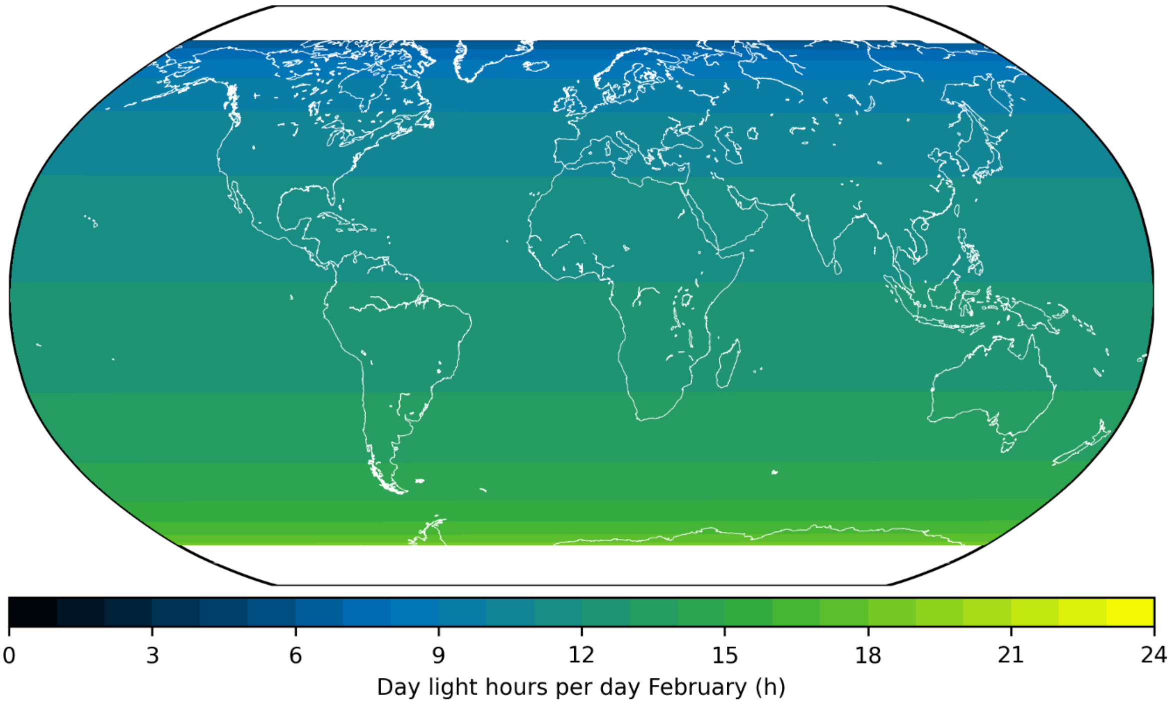

Figure S60. Monthly average daylight hours per day.

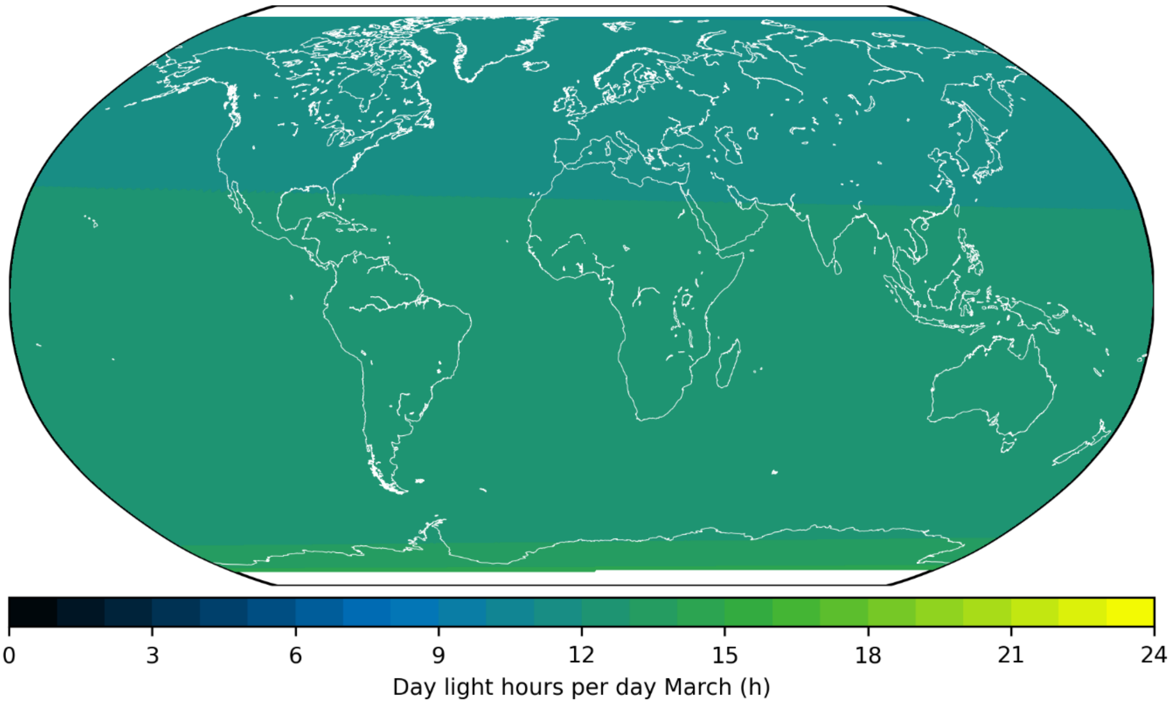

Figure S61. Monthly average daylight hours per day.

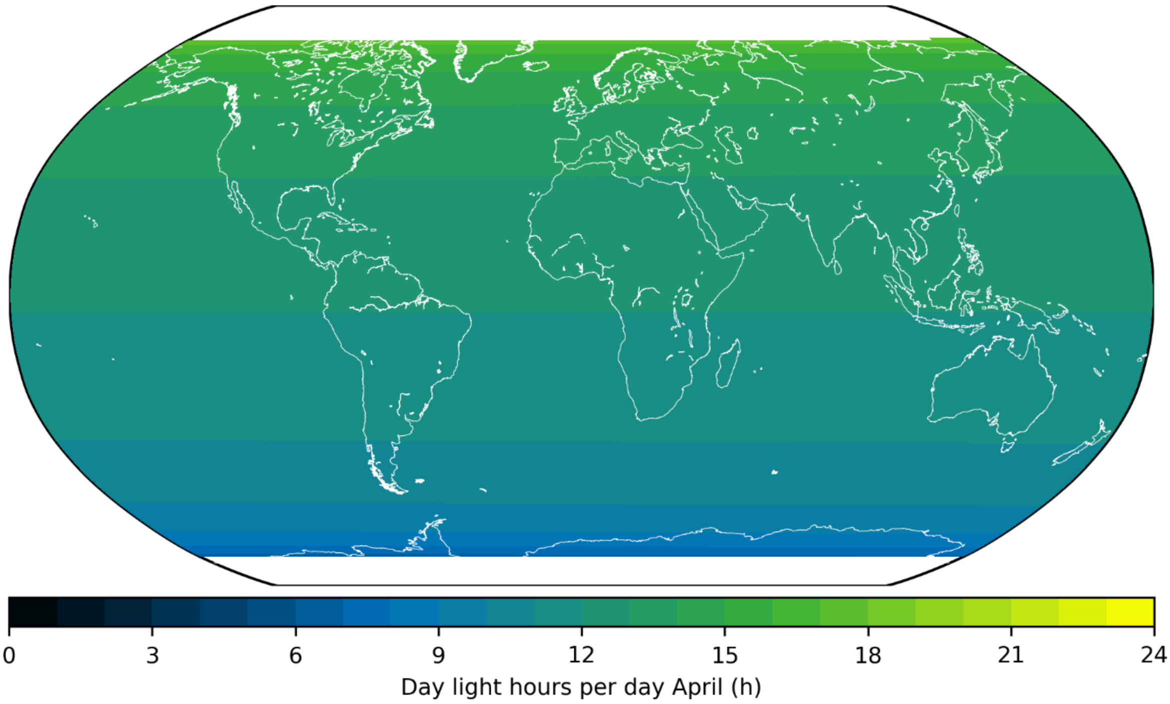

Figure S62. Monthly average daylight hours per day.

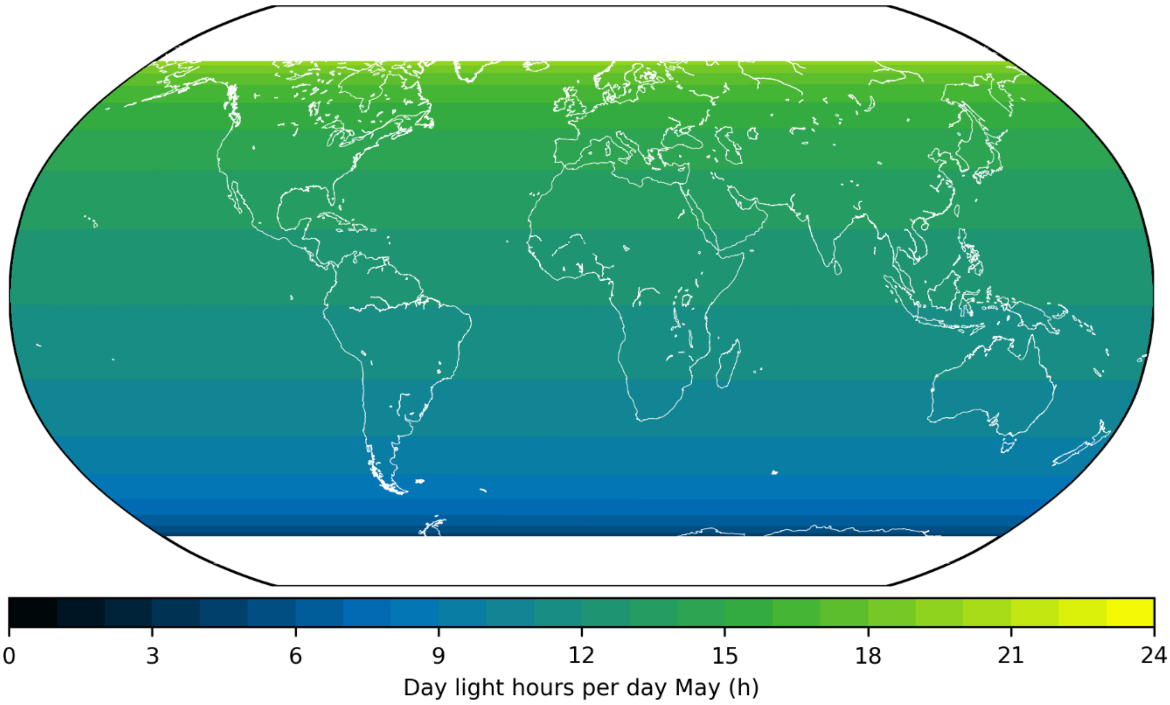

Figure S63. Monthly average daylight hours per day.

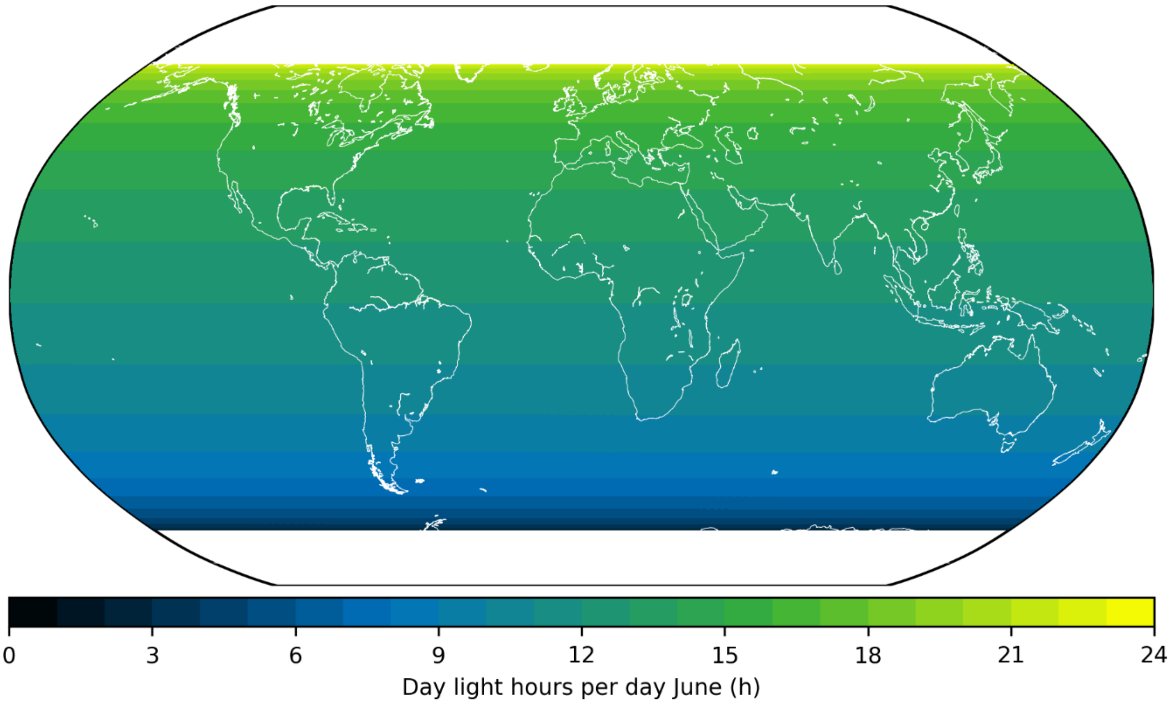

Figure S64. Monthly average daylight hours per day.

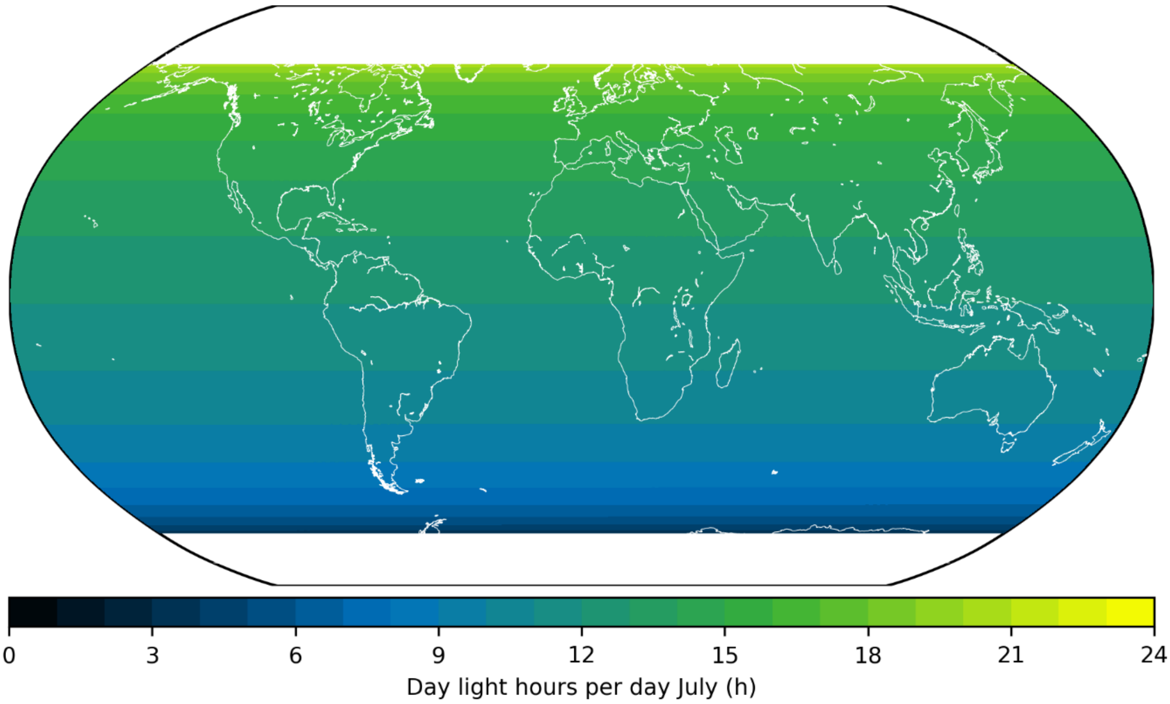

Figure S65. Monthly average daylight hours per day.

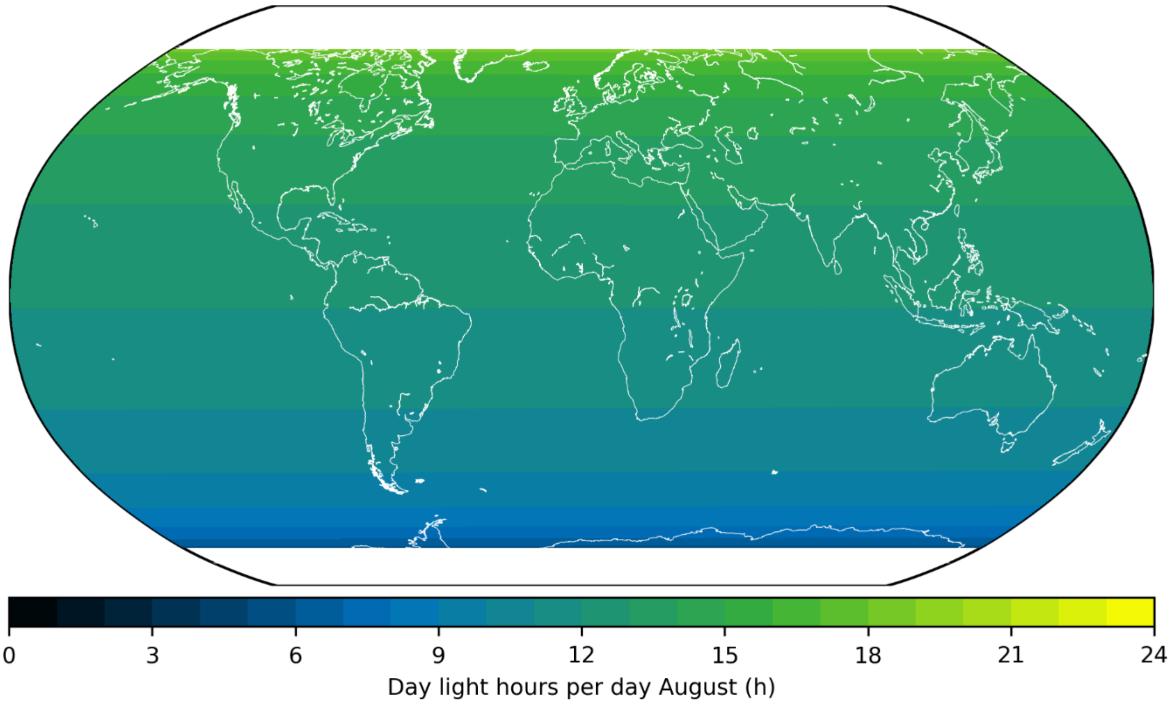

Figure S66. Monthly average daylight hours per day.

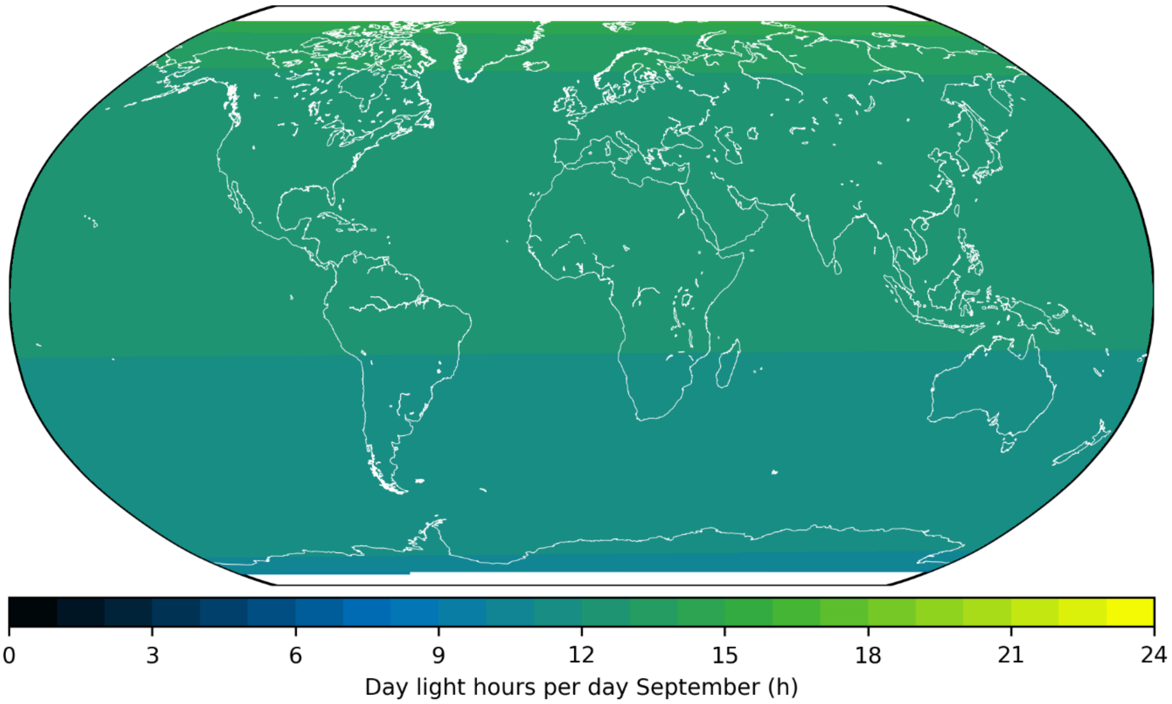

Figure S67. Monthly average daylight hours per day.

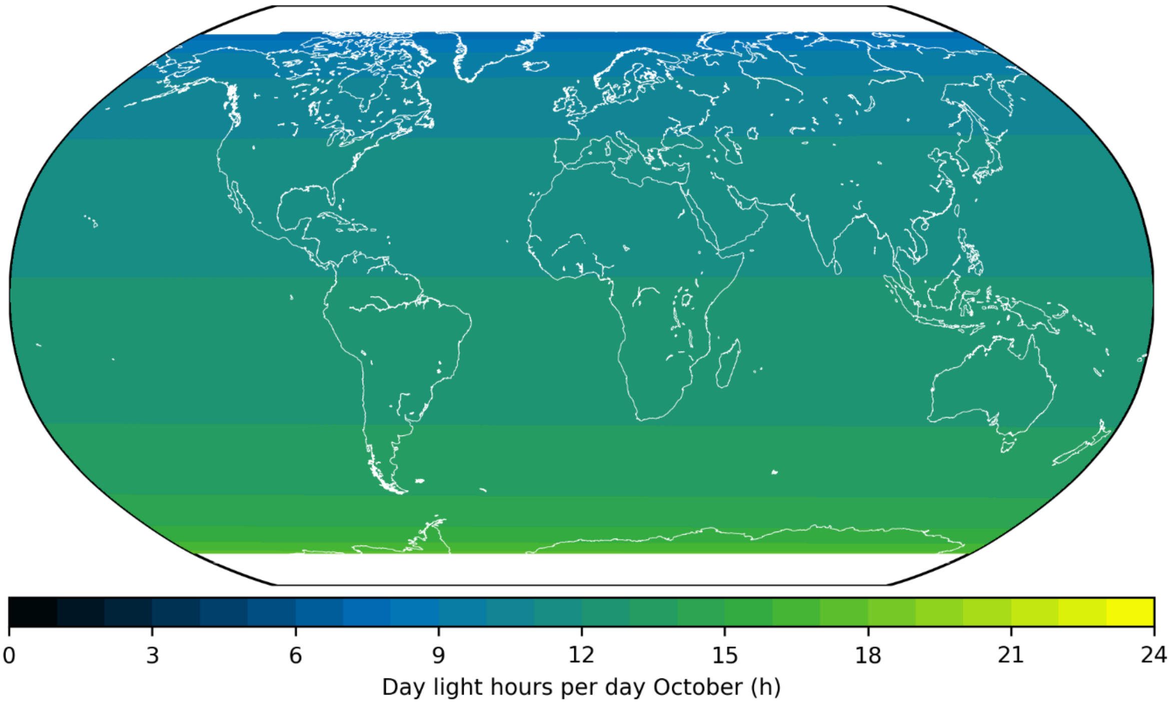

Figure S68. Monthly average daylight hours per day.

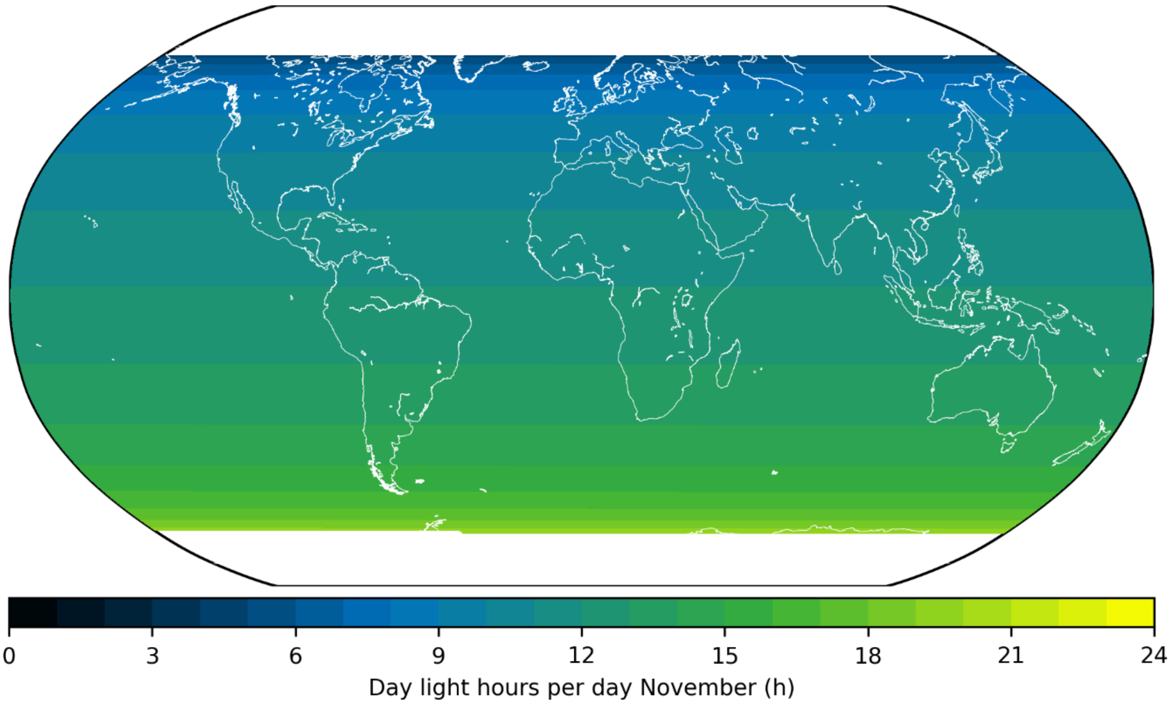

Figure S69. Monthly average daylight hours per day.

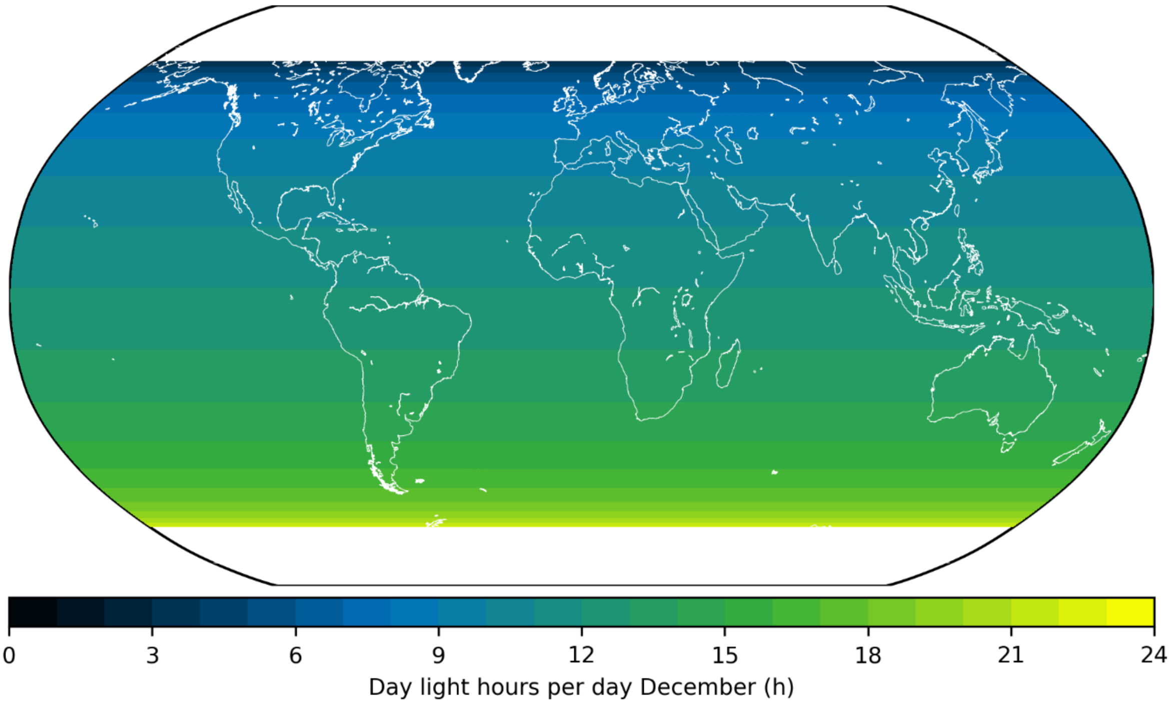

Figure S70. Monthly average daylight hours per day.

8.2. Monthly average peak UVI-index

Calculated using Equation S11 from the monthly average all-sky UV index data of 2023 from the National Aeronautics and Space Administration (NASA) – *The Power Project*.[8]

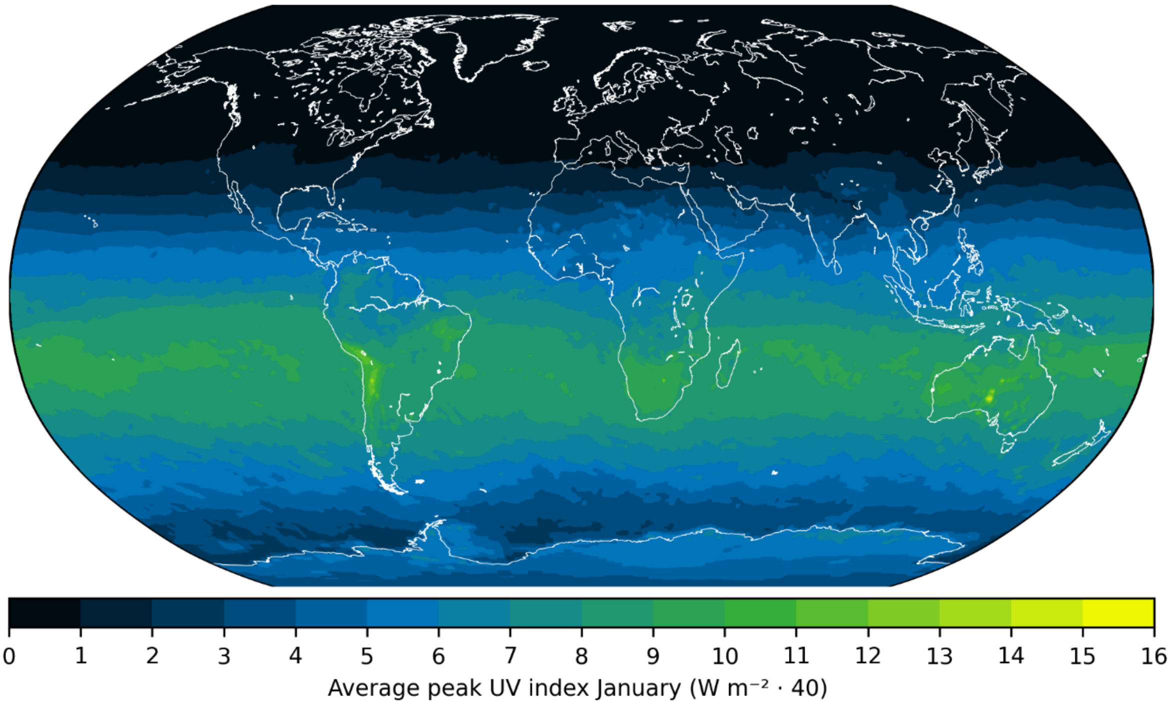

Figure 71. Monthly average peak UV index.

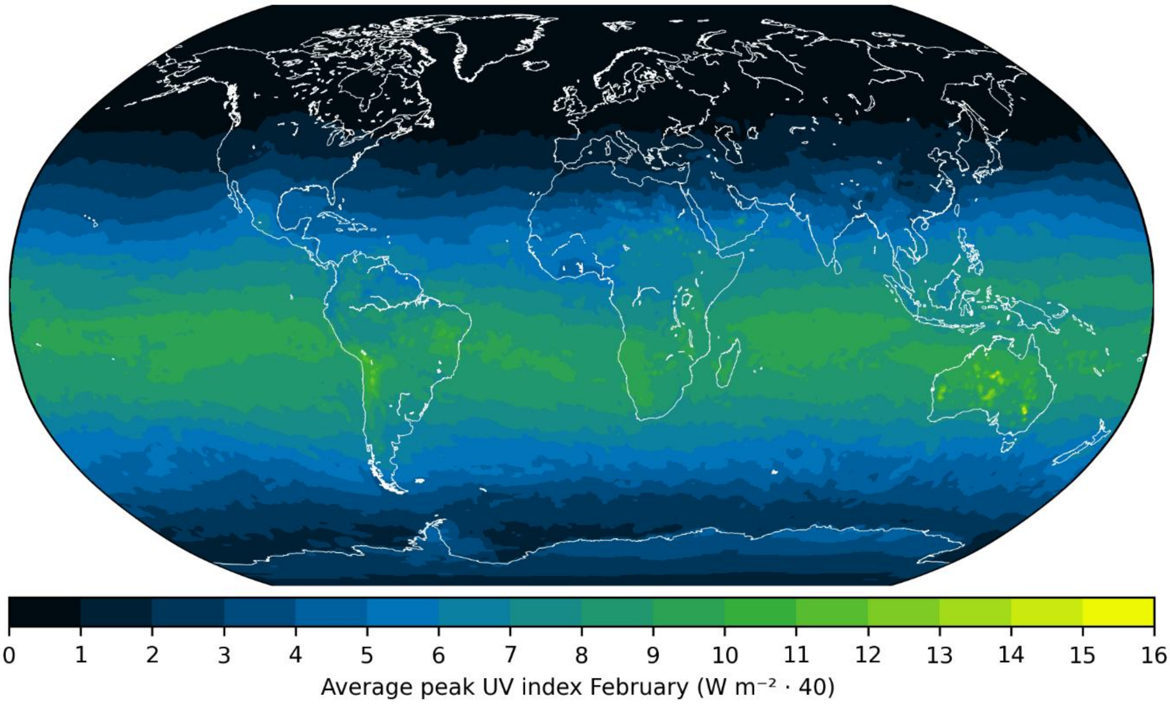

Figure S72. Monthly average peak UV index.

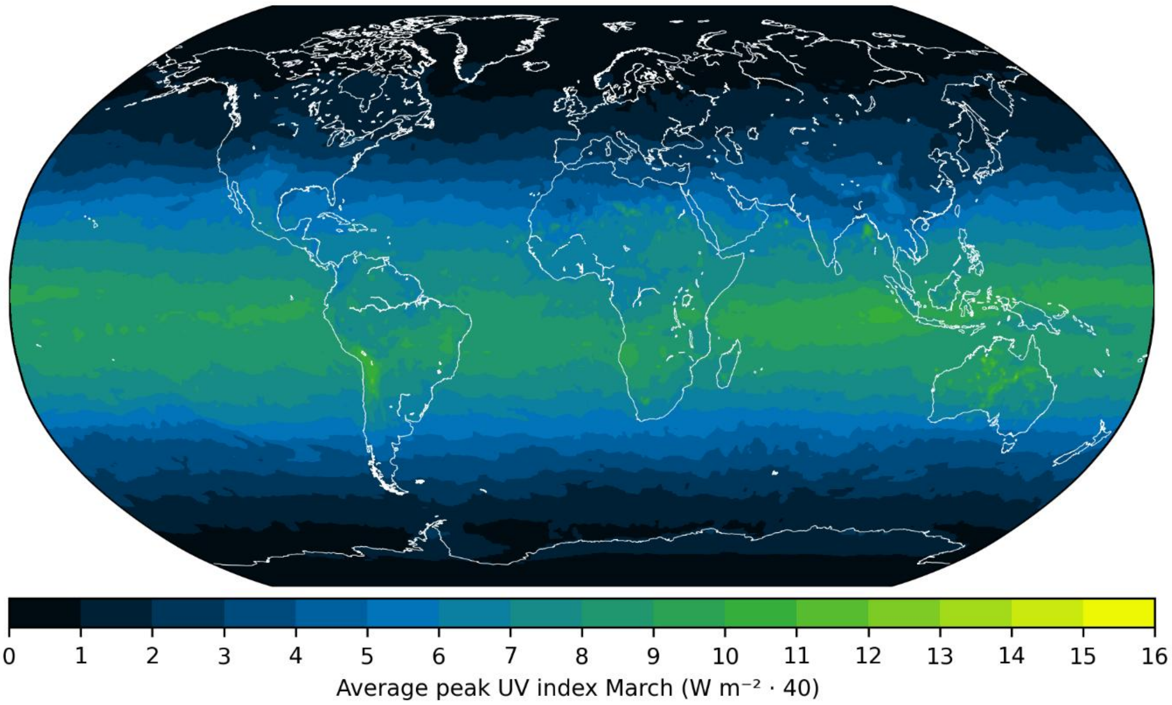

Figure S73. Monthly average peak UV index.

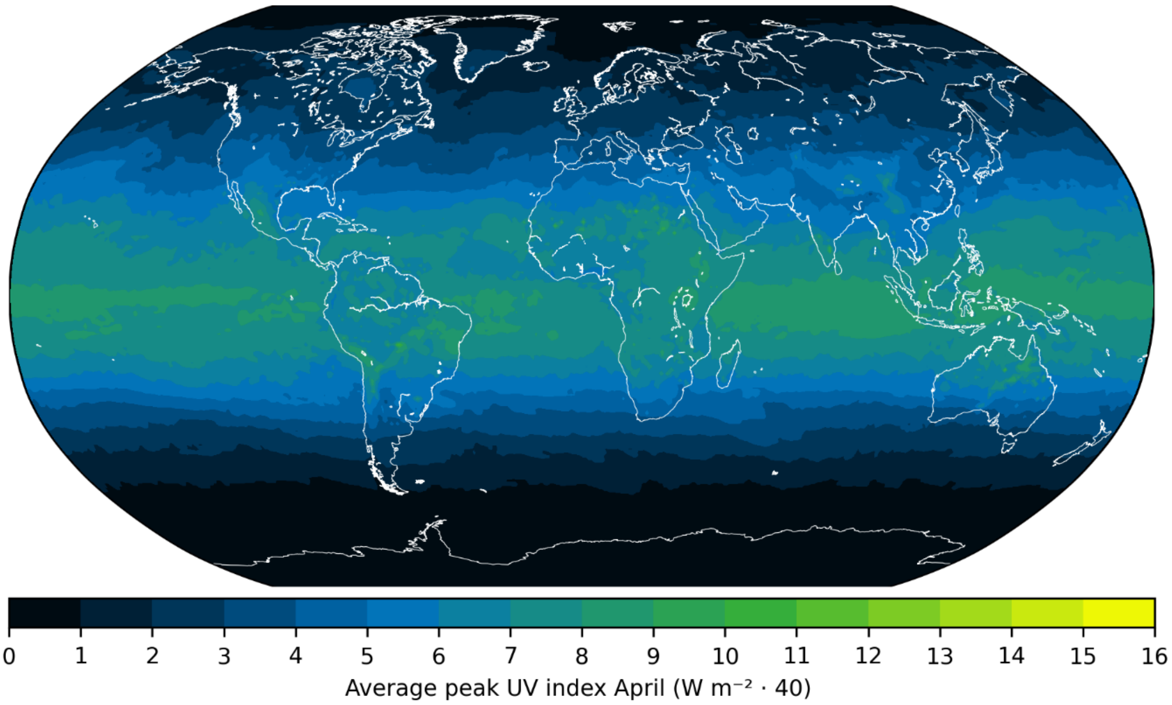

Figure S74. Monthly average peak UV index.

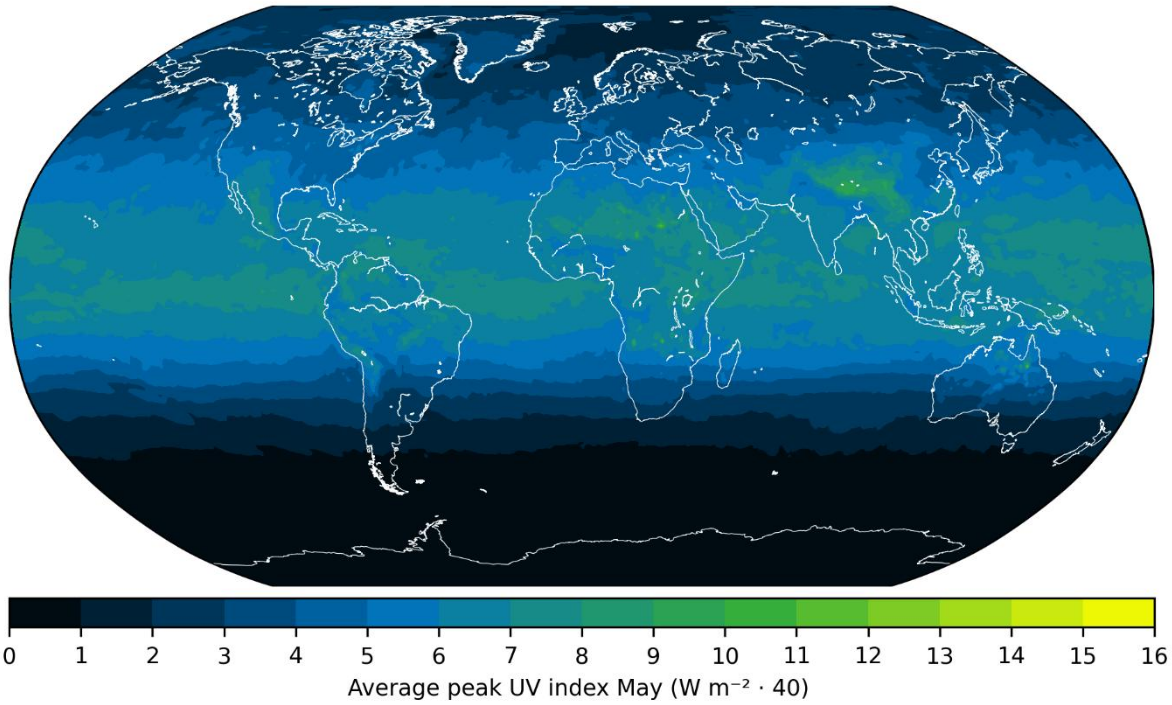

Figure S75. Monthly average peak UV index.

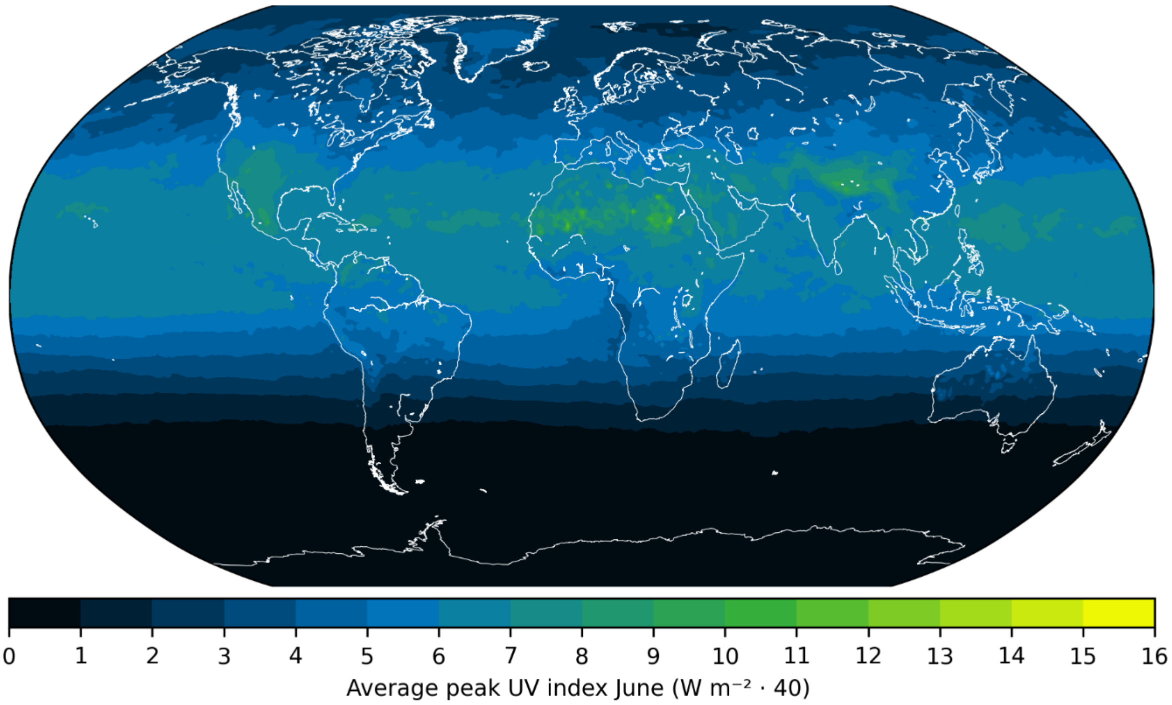

Figure S76. Monthly average peak UV index.

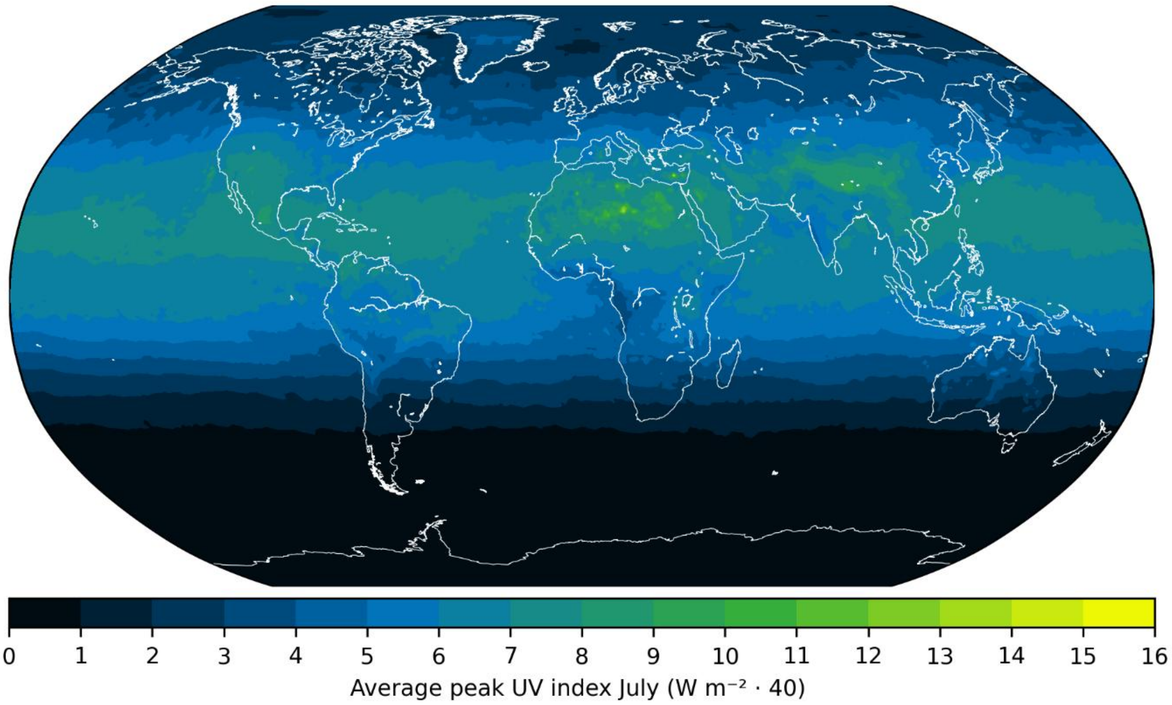

Figure S77. Monthly average peak UV index.

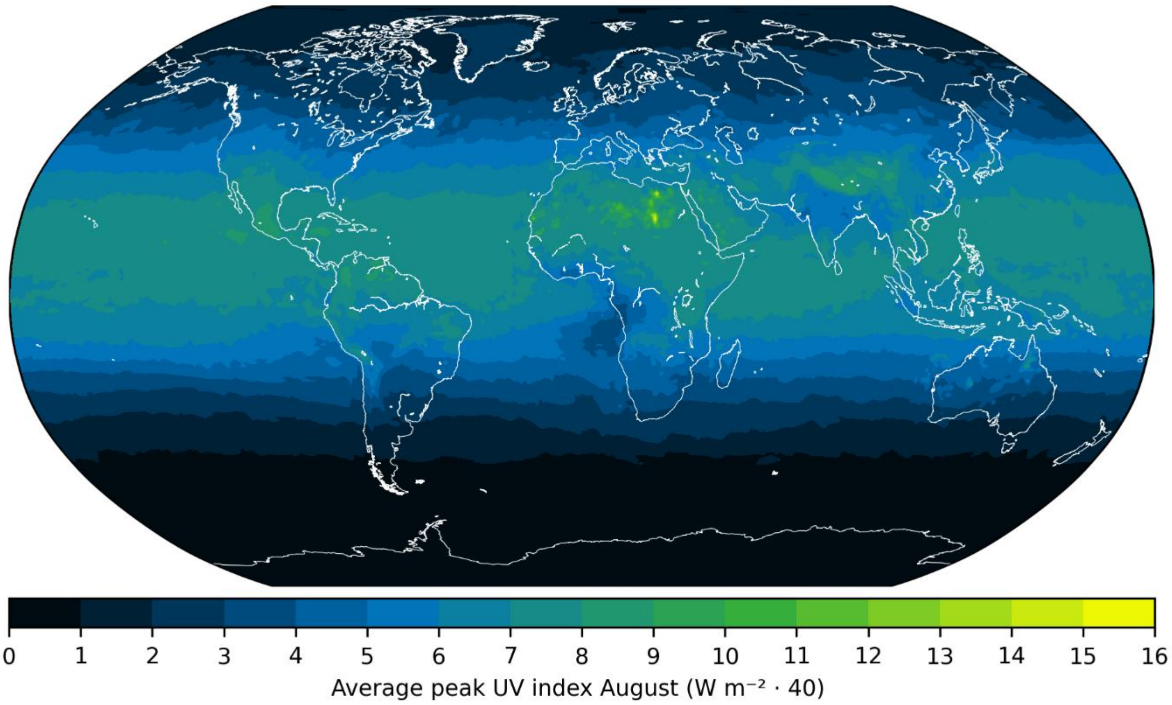

Figure S78. Monthly average peak UV index.

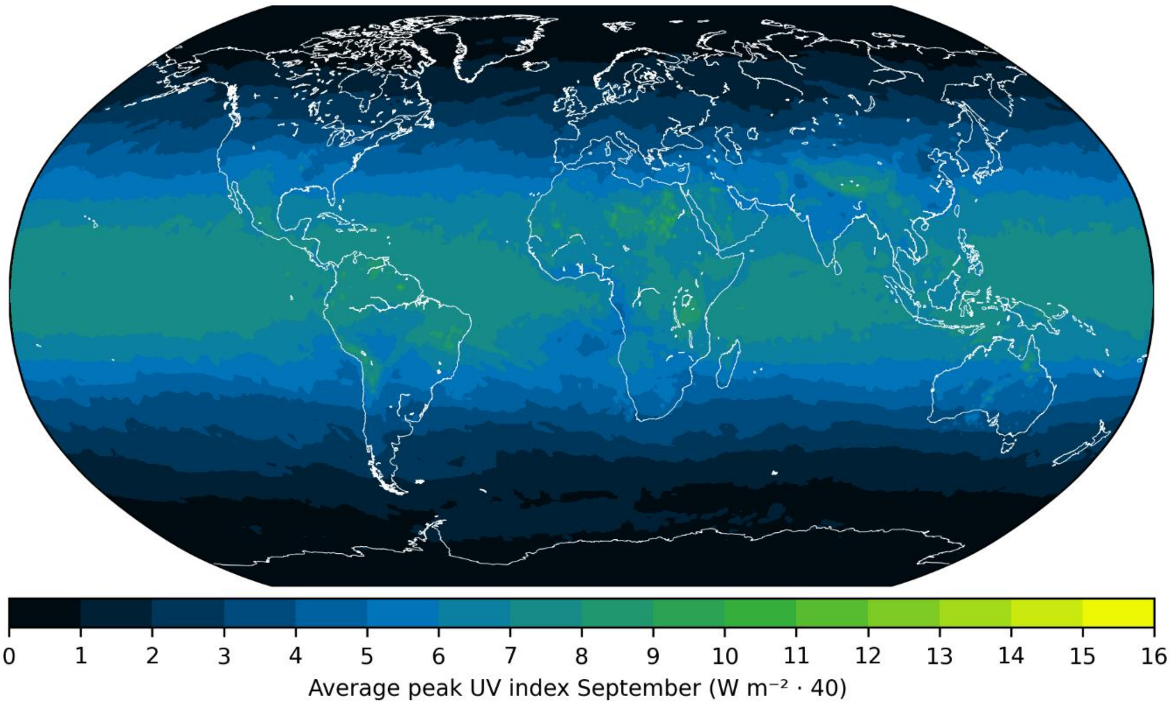

Figure S79. Monthly average peak UV index.

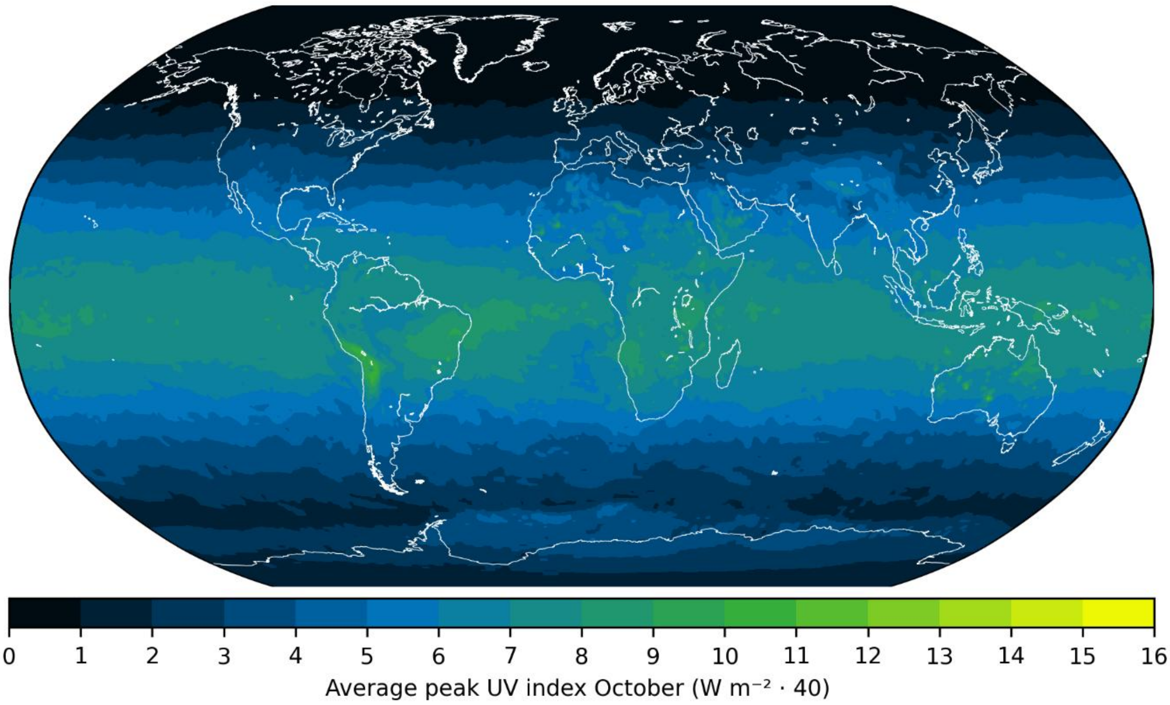

Figure S80. Monthly average peak UV index.

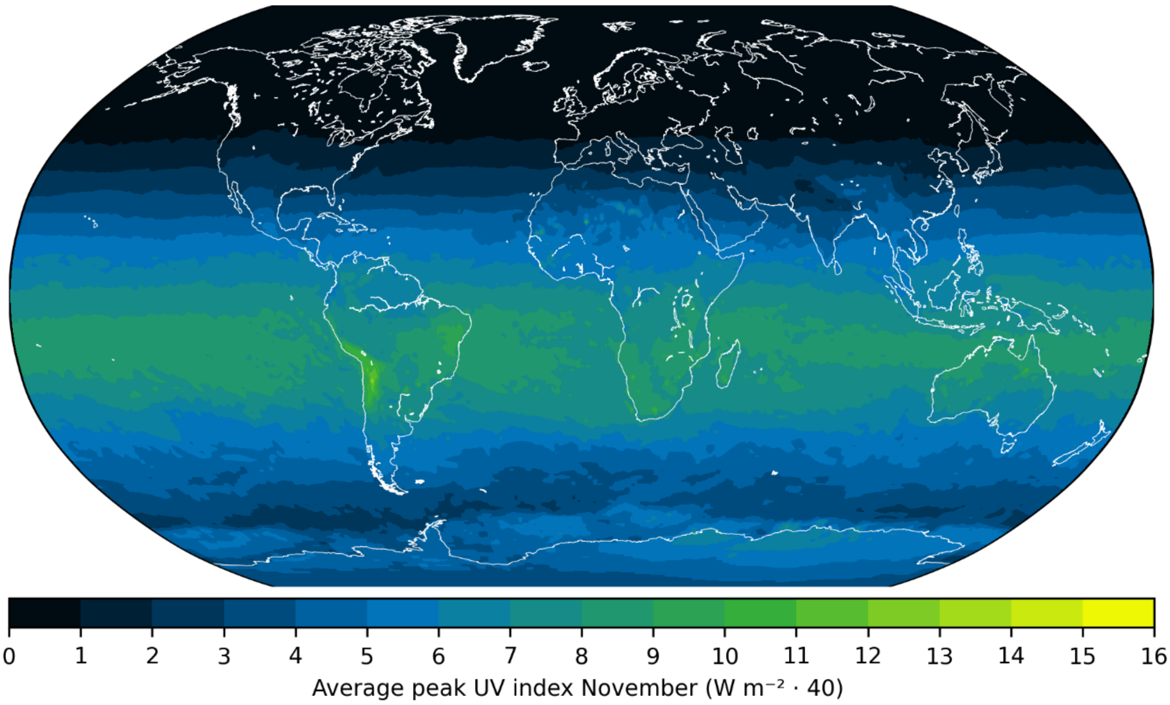

Figure S81. Monthly average peak UV index.

8.3. Optimal UV index at reaction start

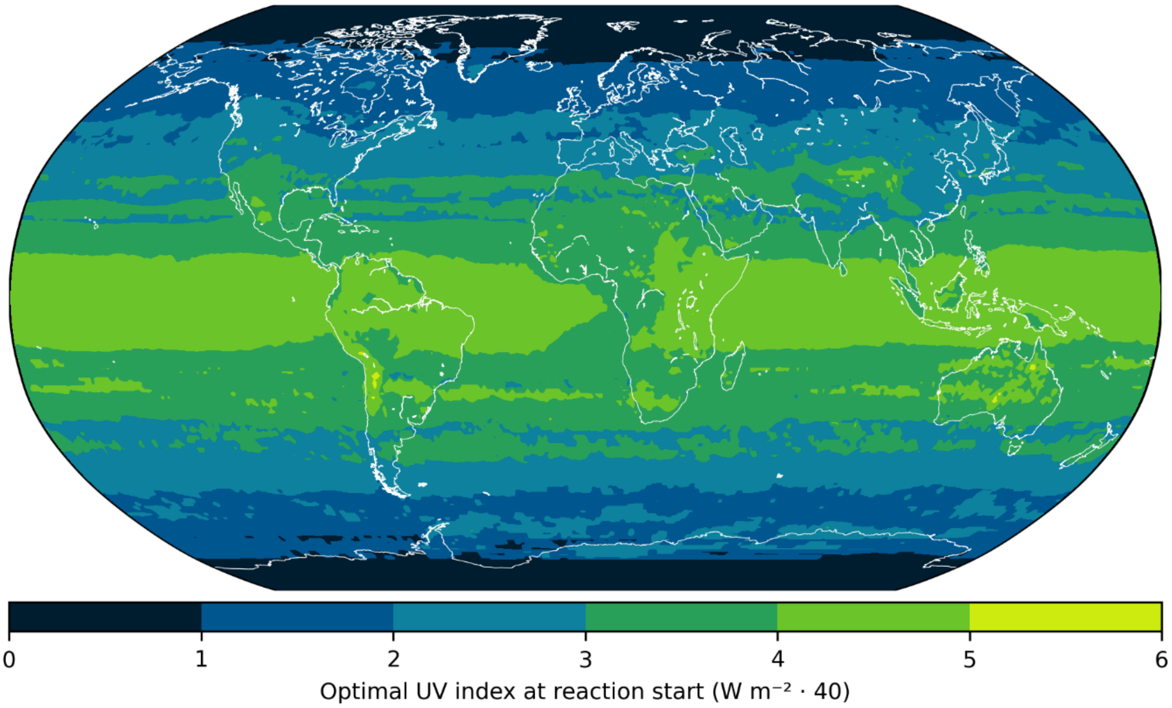

Figure S82. Optimal UV index at reaction start to obtain the highest yield throughout the year. The optimal UV index is equal for 2 mm and 3 mm diameter tubing.

8.4. Monthly resolved yield per area and day

8.4.1. 2 mm diameter tubing

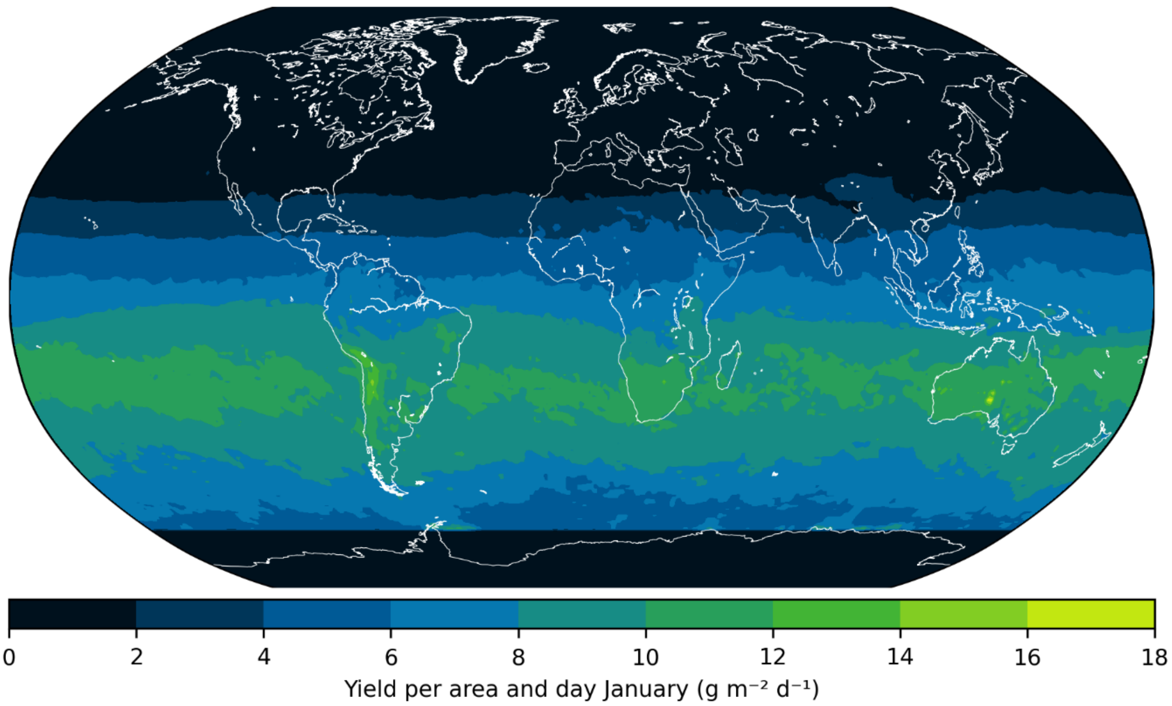

Figure S83. Monthly average yield per unit area and day for 2 mm diameter tubing.

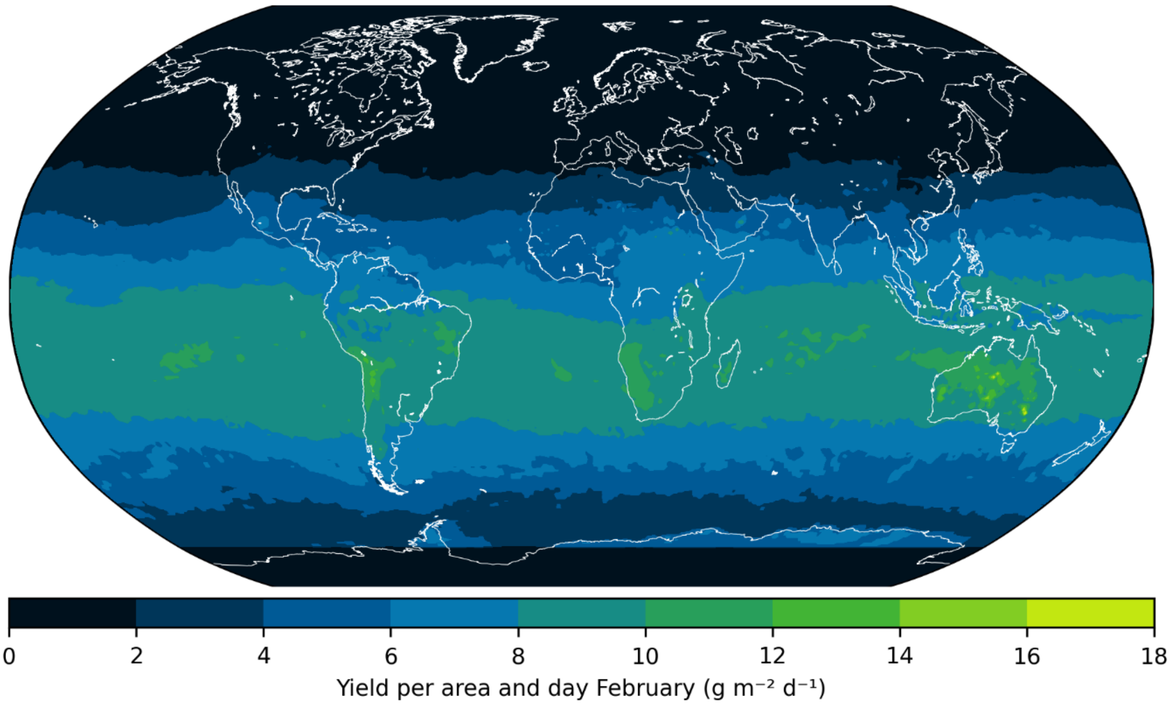

Figure S84. Monthly average yield per unit area and day for 2 mm diameter tubing.

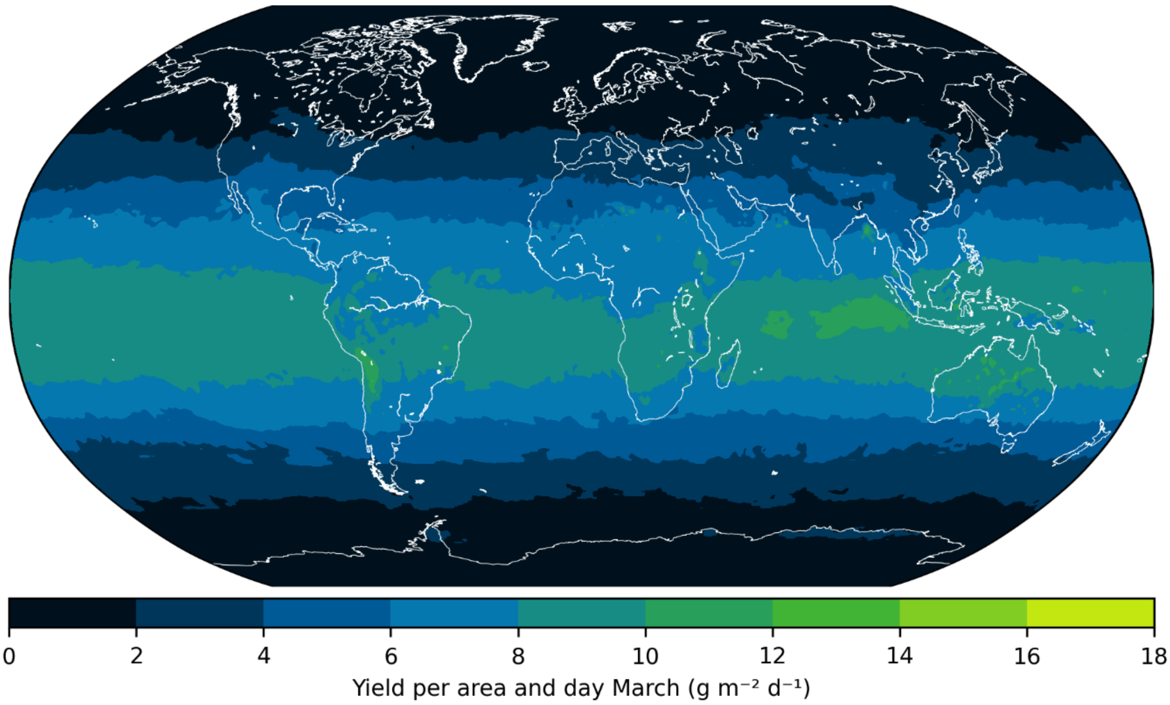

Figure S85. Monthly average yield per unit area and day for 2 mm diameter tubing.

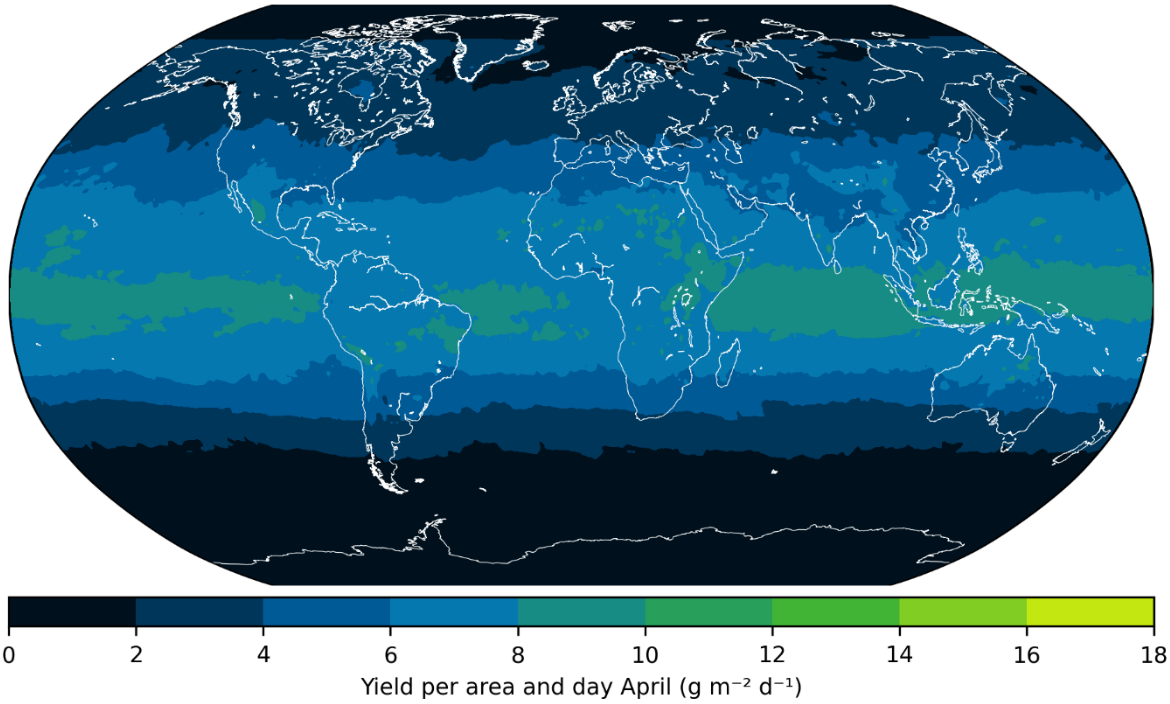

Figure S86. Monthly average yield per unit area and day for 2 mm diameter tubing.

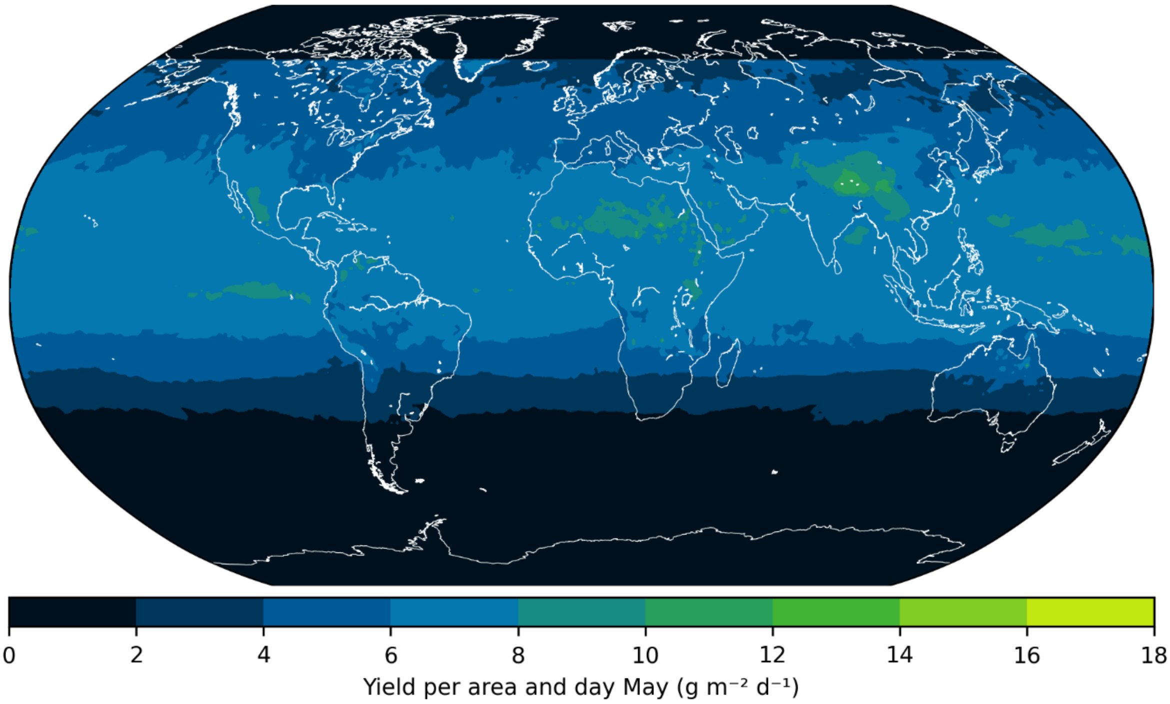

Figure S87. Monthly average yield per unit area and day for 2 mm diameter tubing.

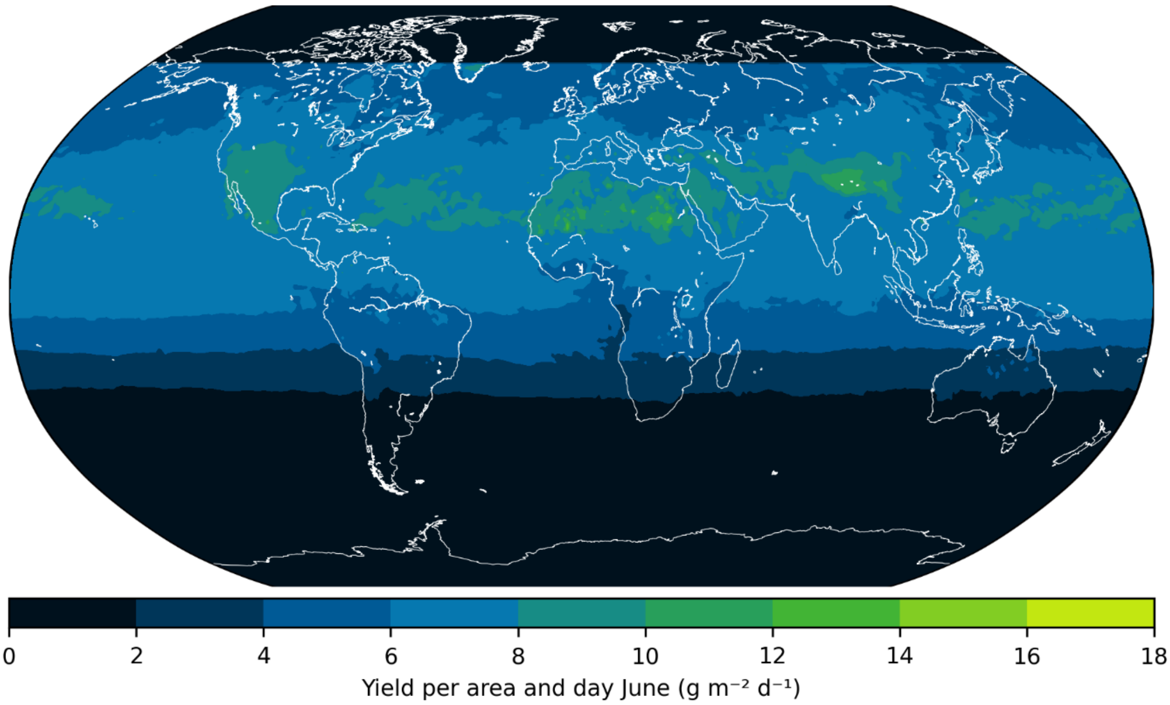

Figure S88. Monthly average yield per unit area and day for 2 mm diameter tubing.

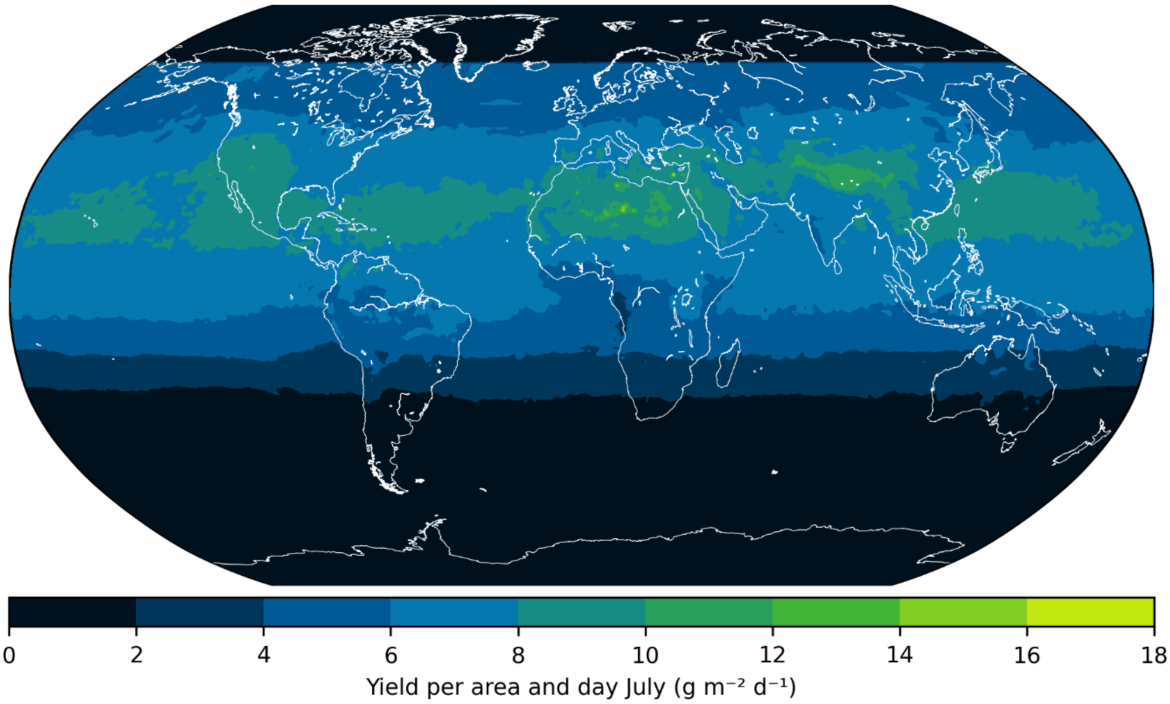

Figure S89. Monthly average yield per unit area and day for 2 mm diameter tubing.

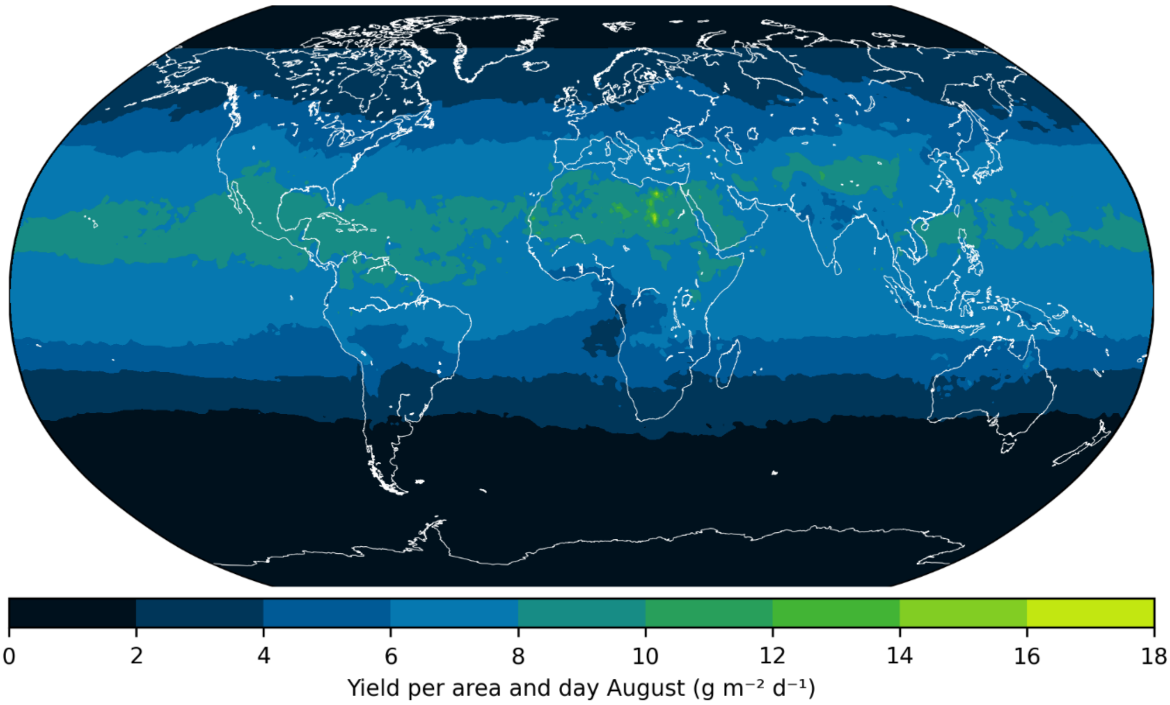

Figure S90. Monthly average yield per unit area and day for 2 mm diameter tubing.

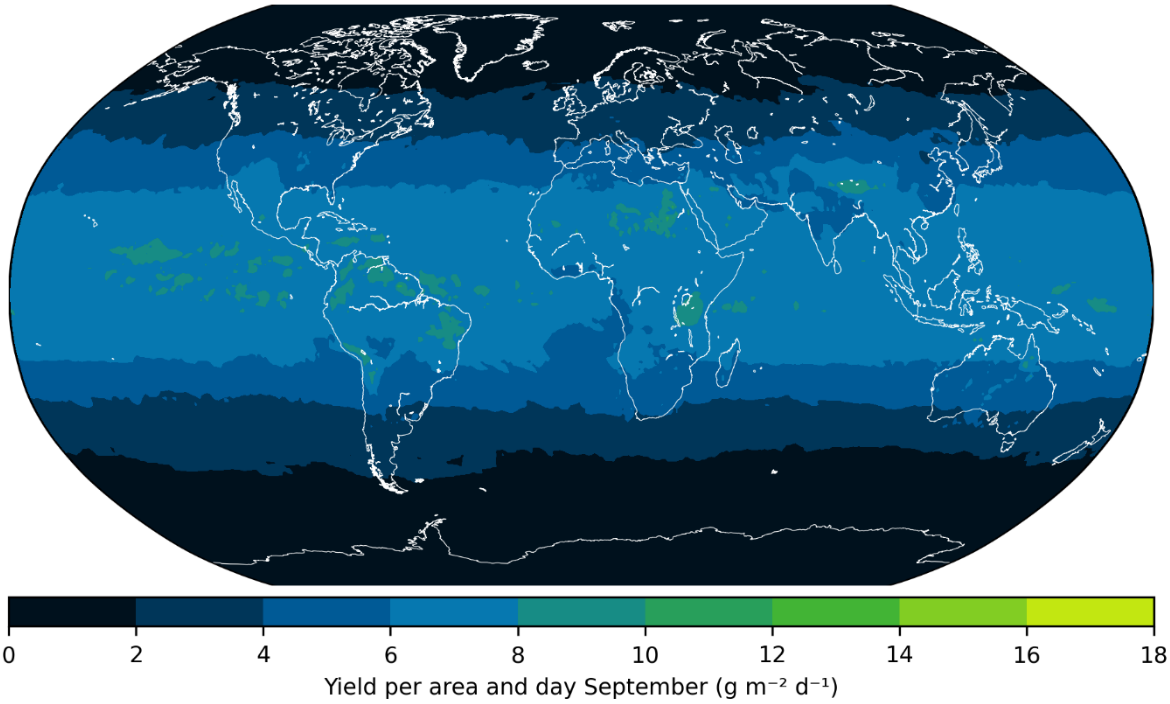

Figure S91. Monthly average yield per unit area and day for 2 mm diameter tubing.

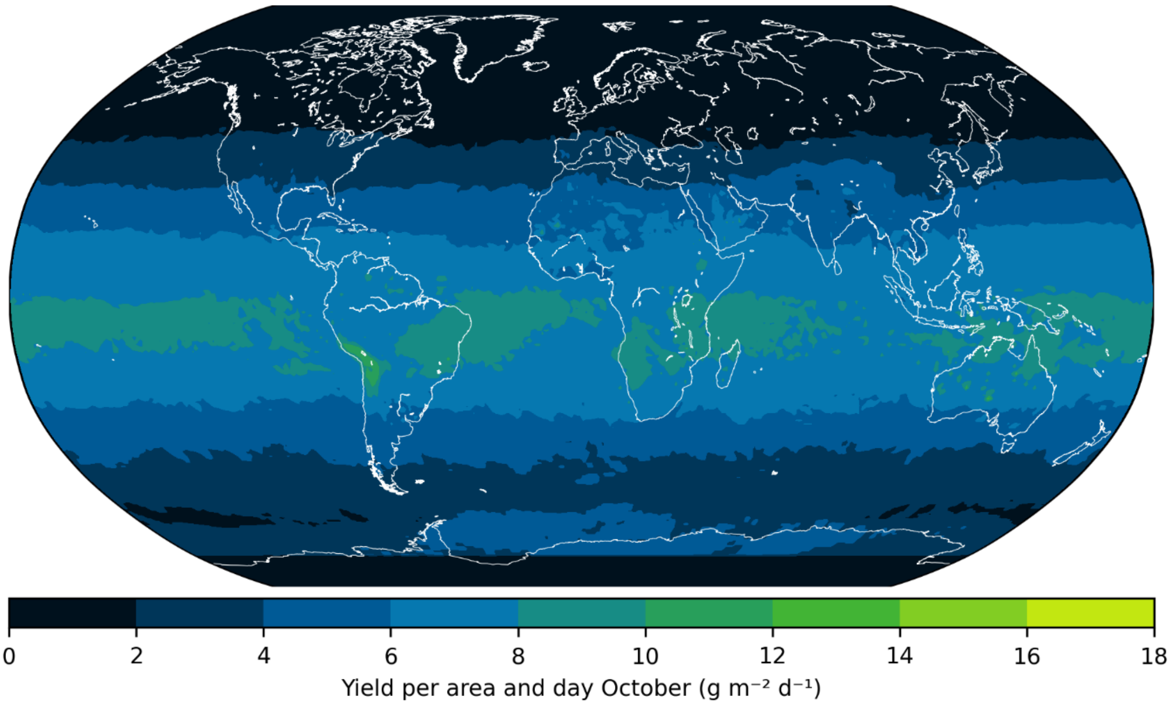

Figure S92. Monthly average yield per unit area and day for 2 mm diameter tubing.

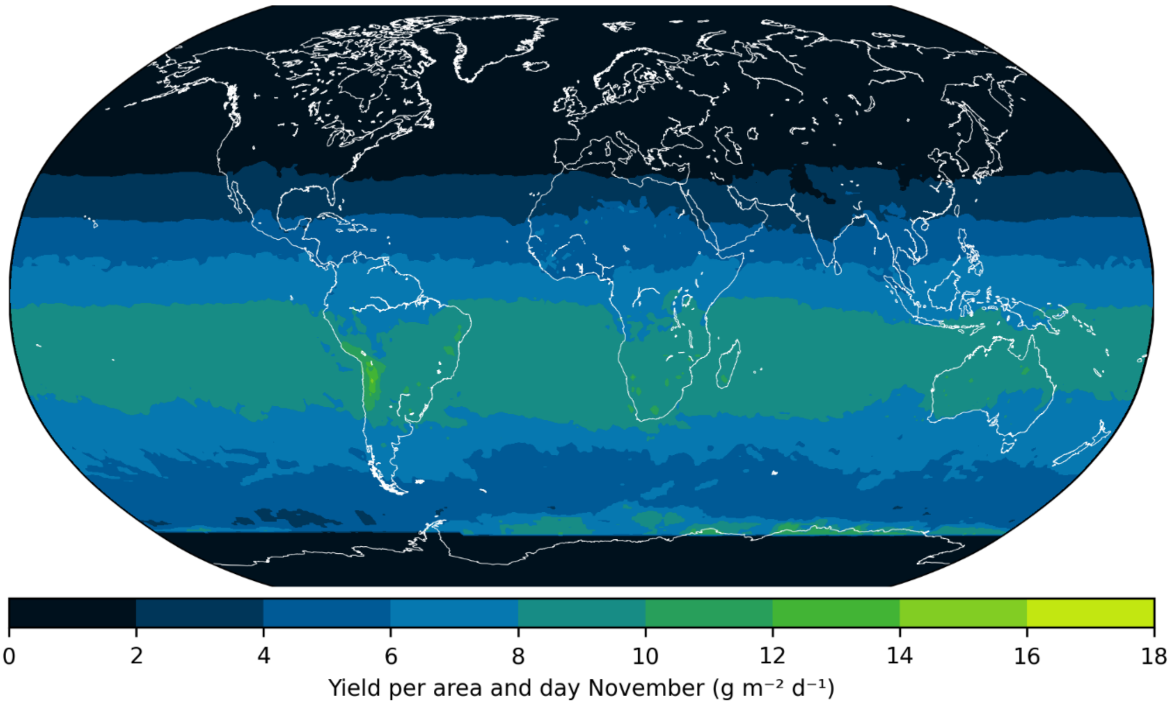

Figure S93. Monthly average yield per unit area and day for 2 mm diameter tubing.

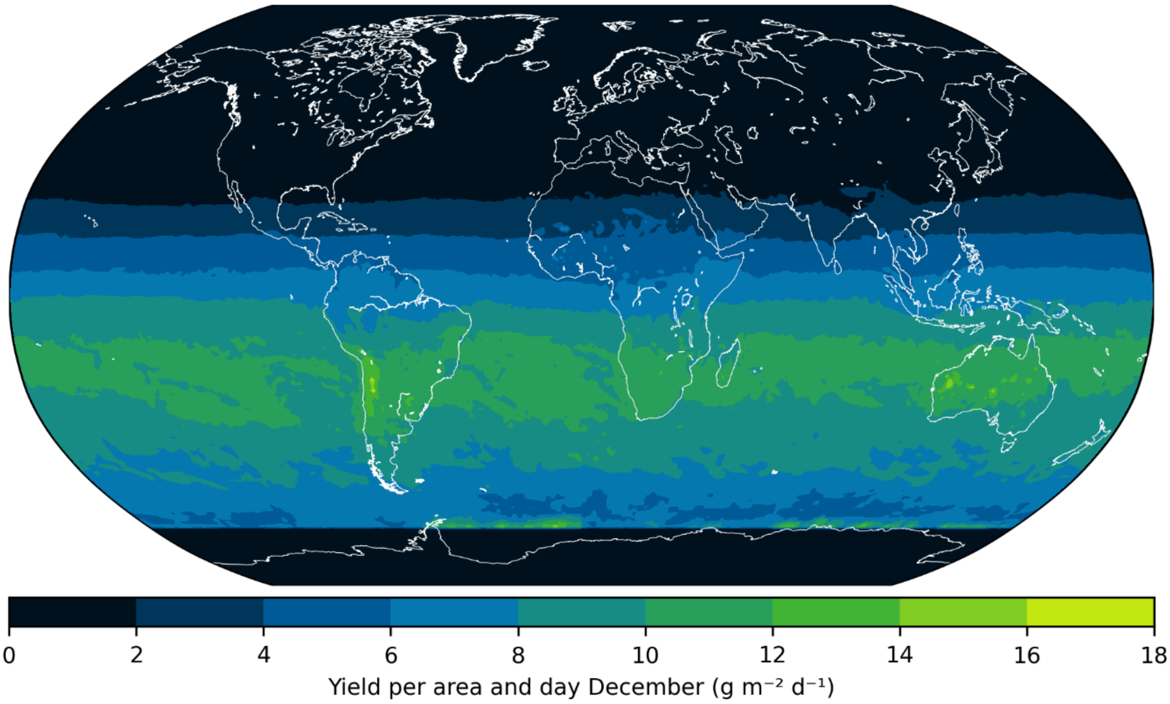

Figure S94. Monthly average yield per unit area and day for 2 mm diameter tubing.

8.4.2. 3 mm diameter tubing

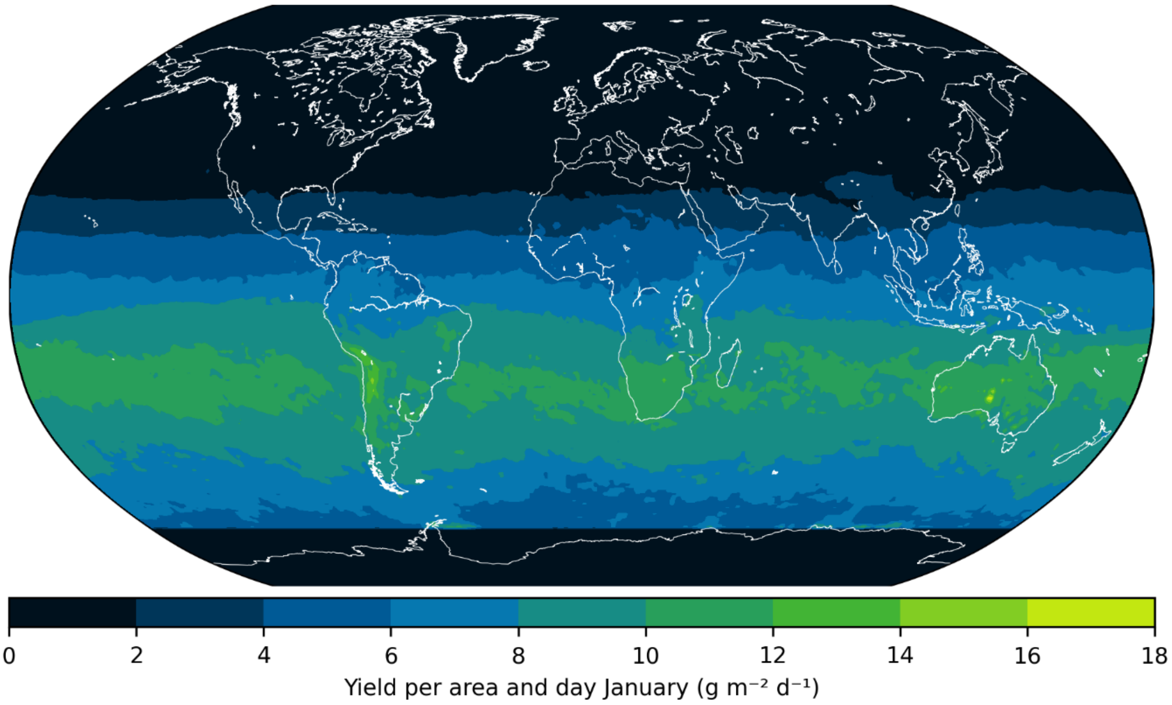

Figure S95. Monthly average yield per unit area and day for 3 mm diameter tubing.

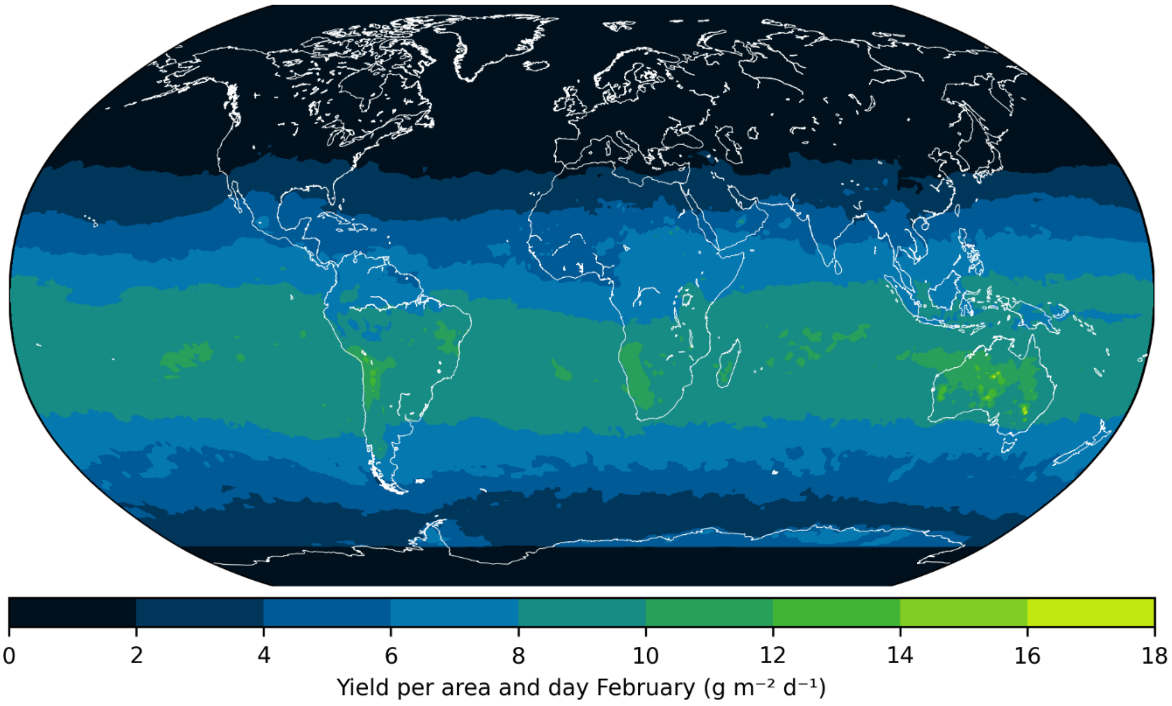

Figure S96. Monthly average yield per unit area and day for 3 mm diameter tubing.

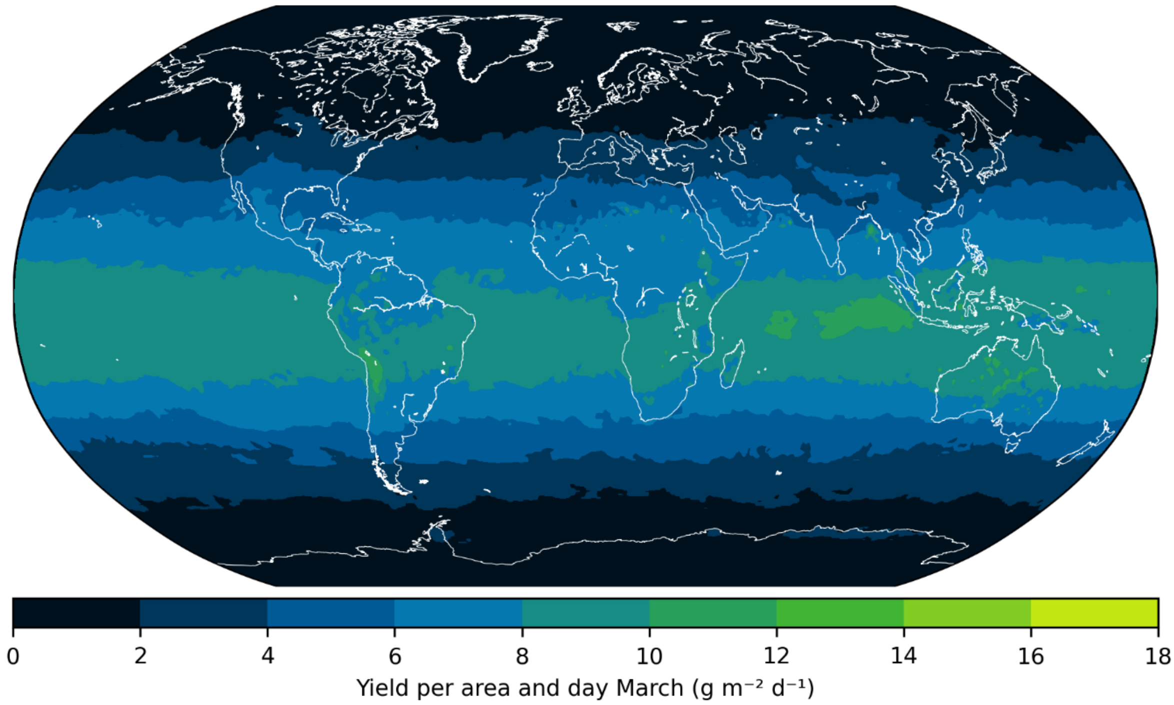

Figure S97. Monthly average yield per unit area and day for 3 mm diameter tubing.

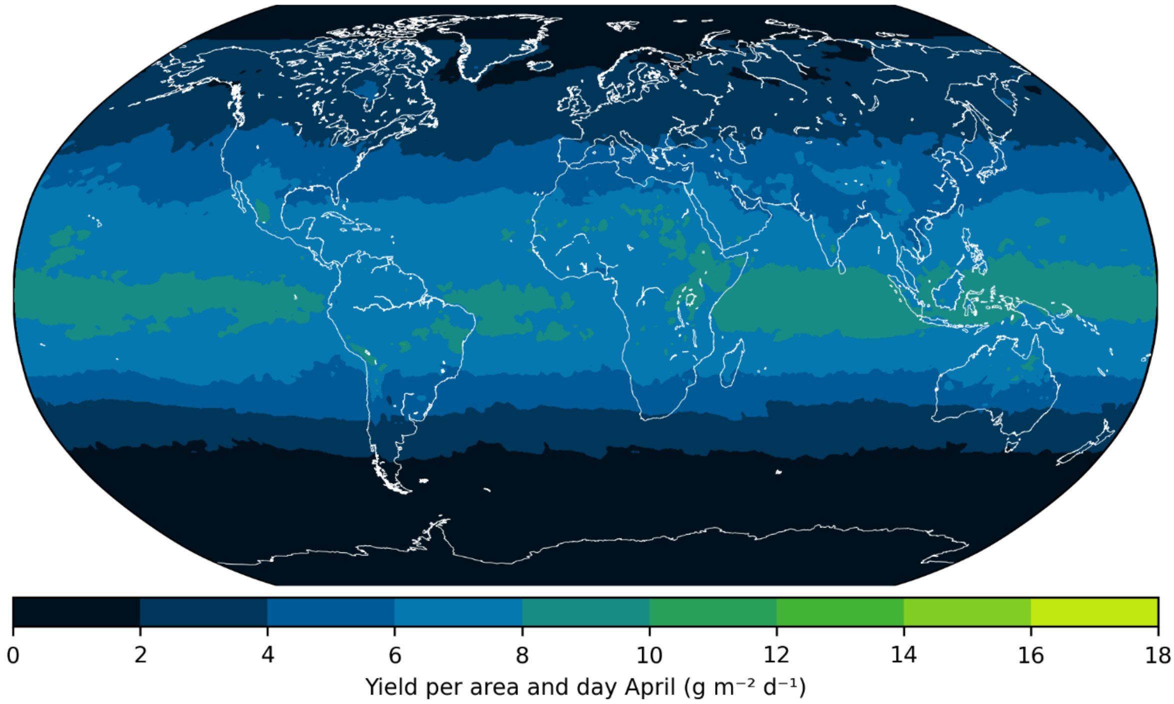

Figure S98. Monthly average yield per unit area and day for 3 mm diameter tubing.

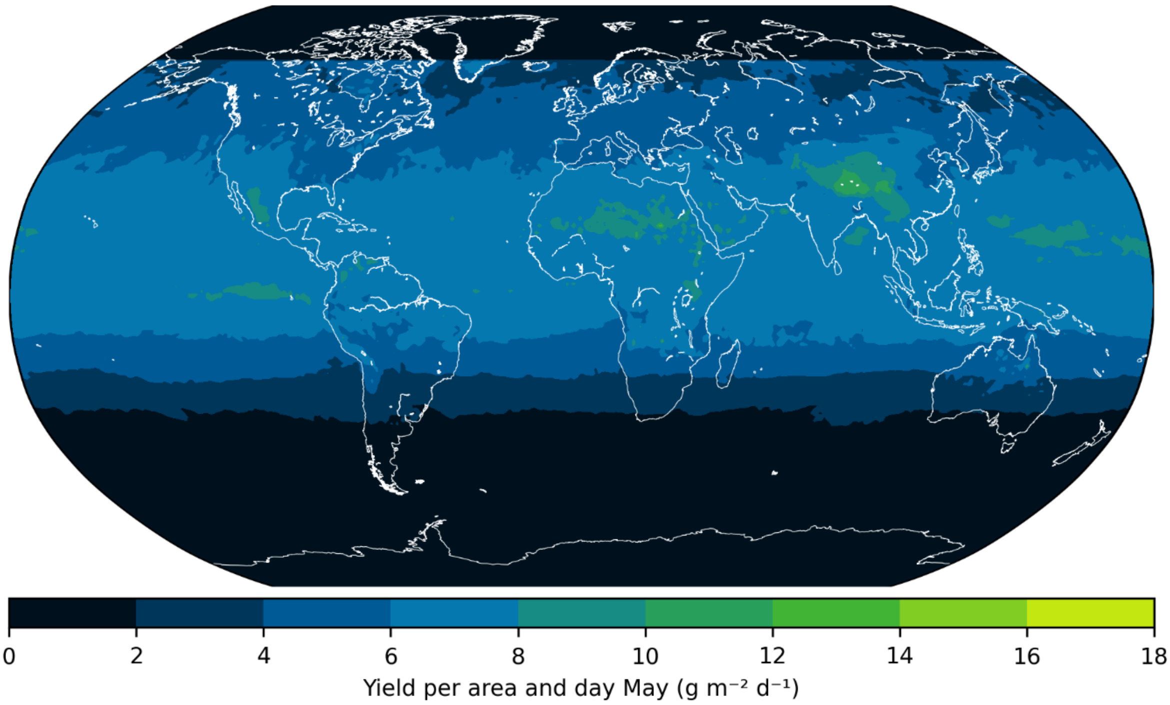

Figure S99. Monthly average yield per unit area and day for 3 mm diameter tubing.

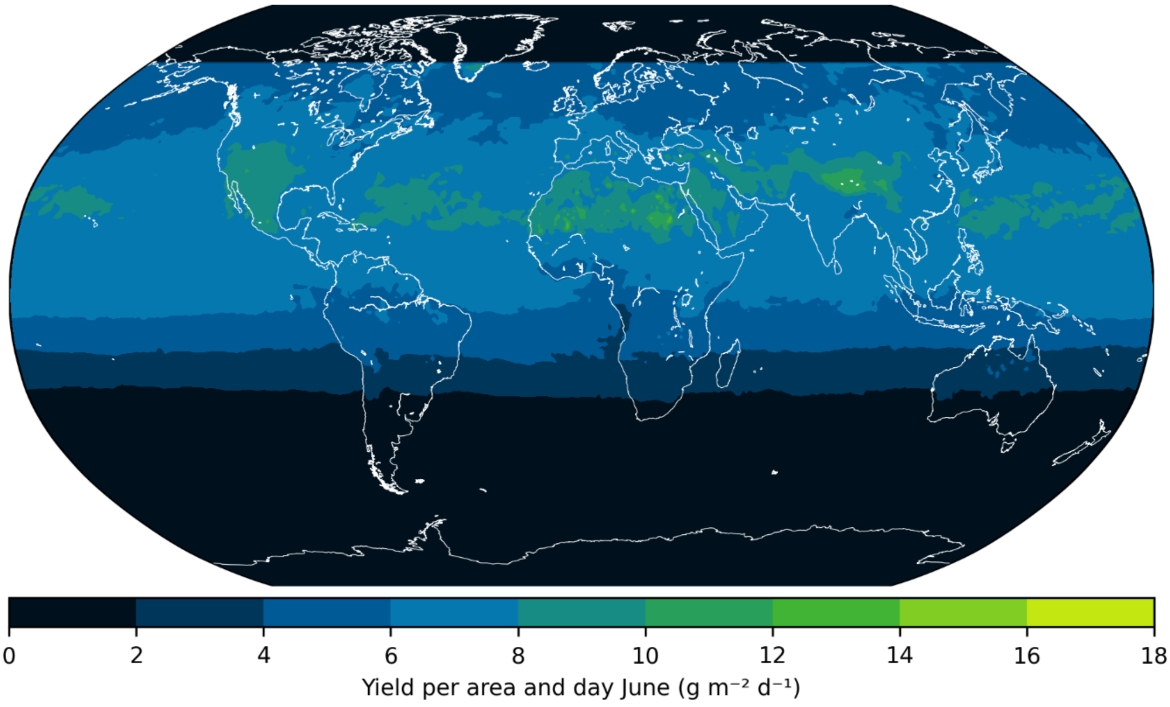

Figure S100. Monthly average yield per unit area and day for 3 mm diameter tubing.

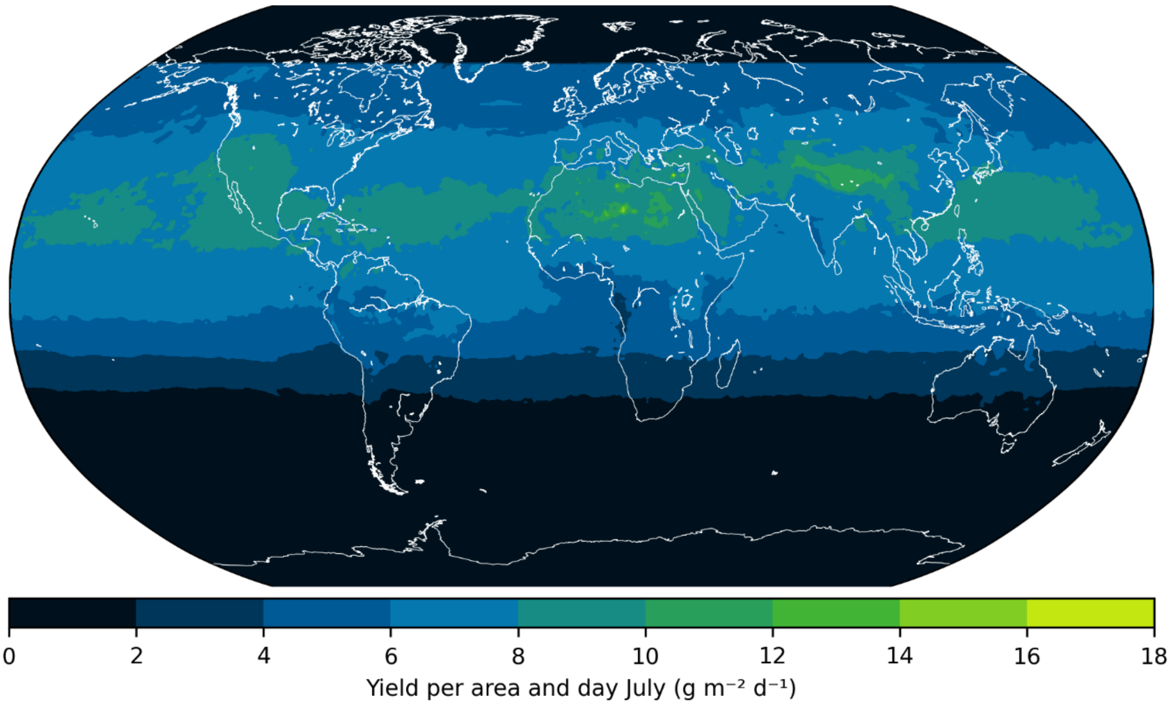

Figure S101. Monthly average yield per unit area and day for 3 mm diameter tubing.

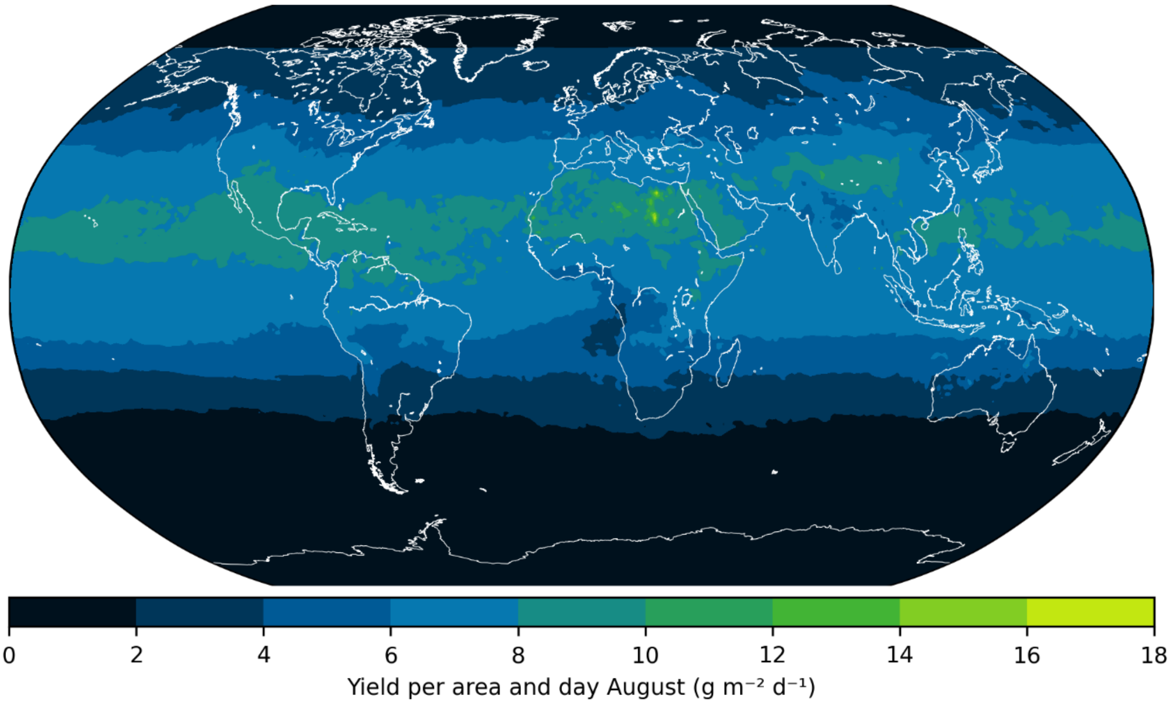

Figure S102. Monthly average yield per unit area and day for 3 mm diameter tubing.

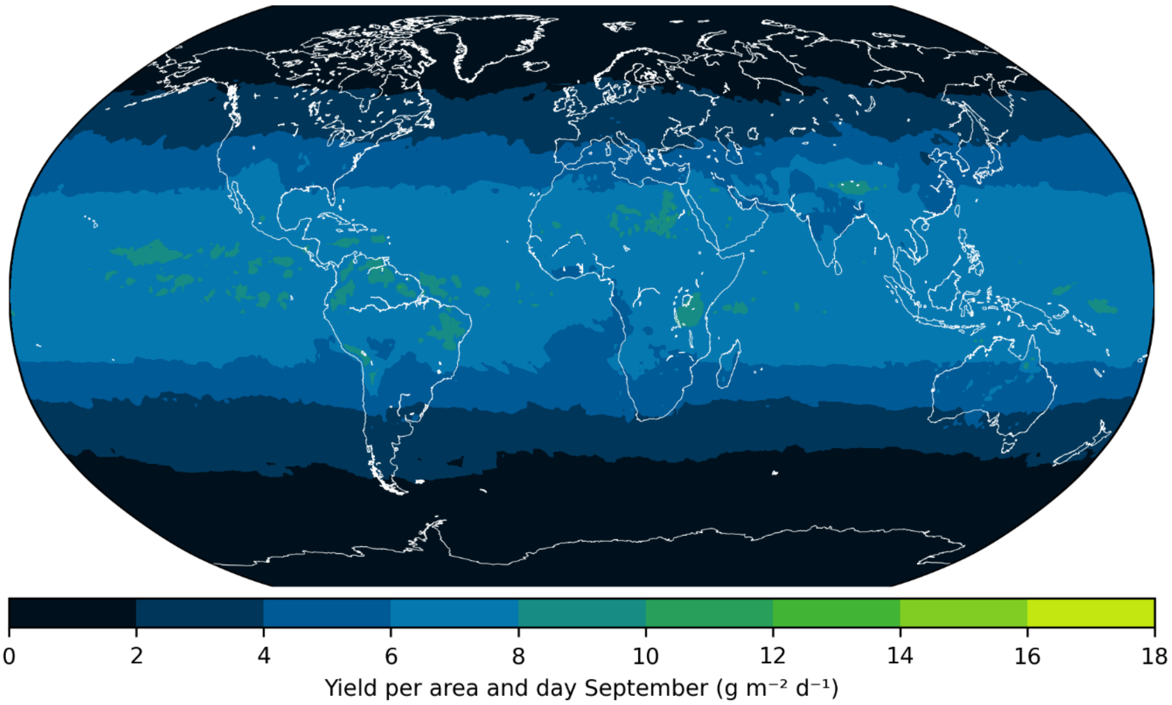

Figure S103. Monthly average yield per unit area and day for 3 mm diameter tubing.

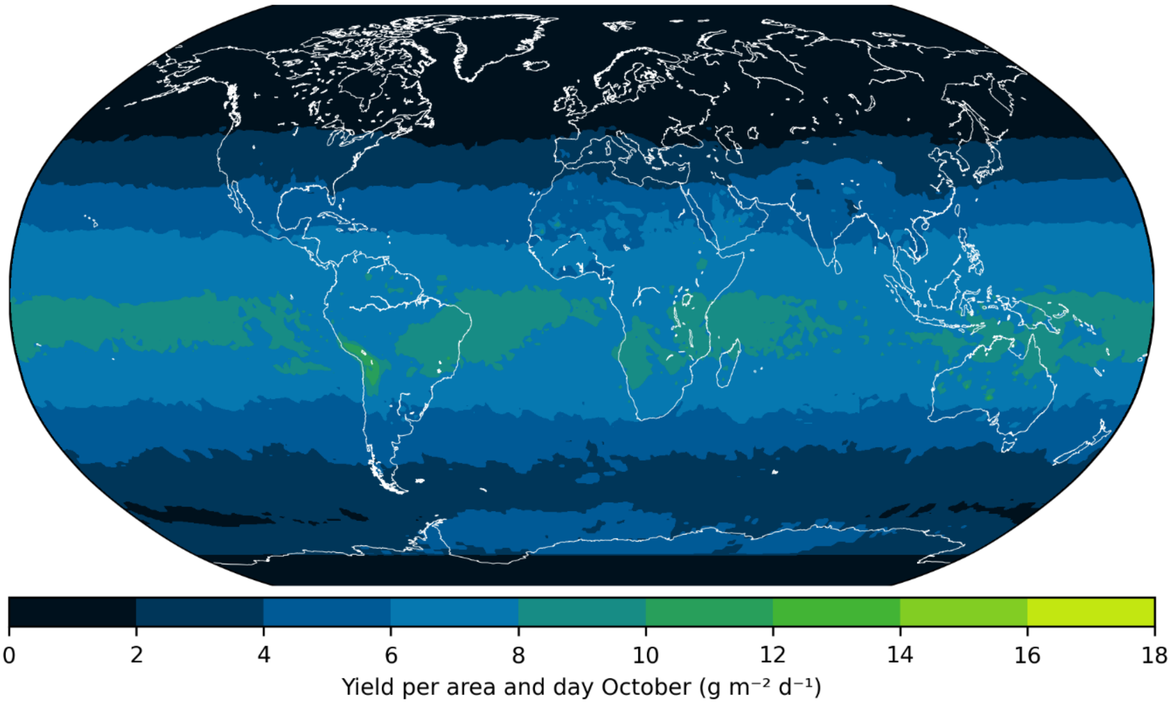

Figure S104. Monthly average yield per unit area and day for 3 mm diameter tubing.

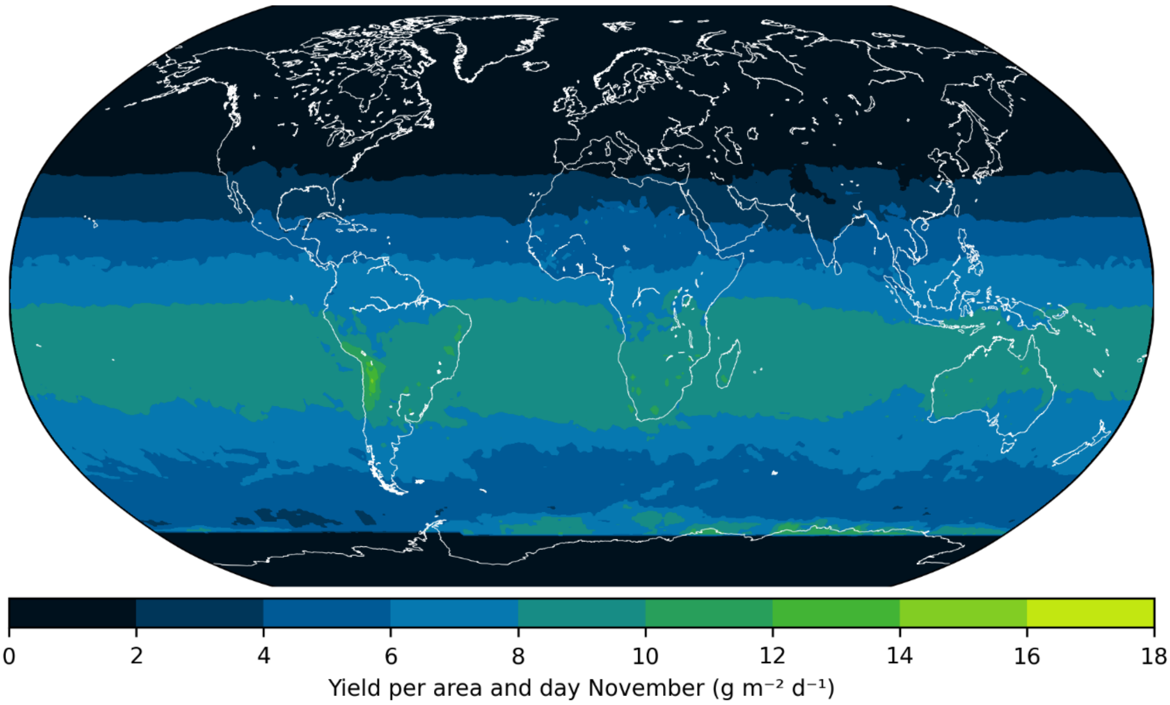

Figure S105. Monthly average yield per unit area and day for 3 mm diameter tubing.

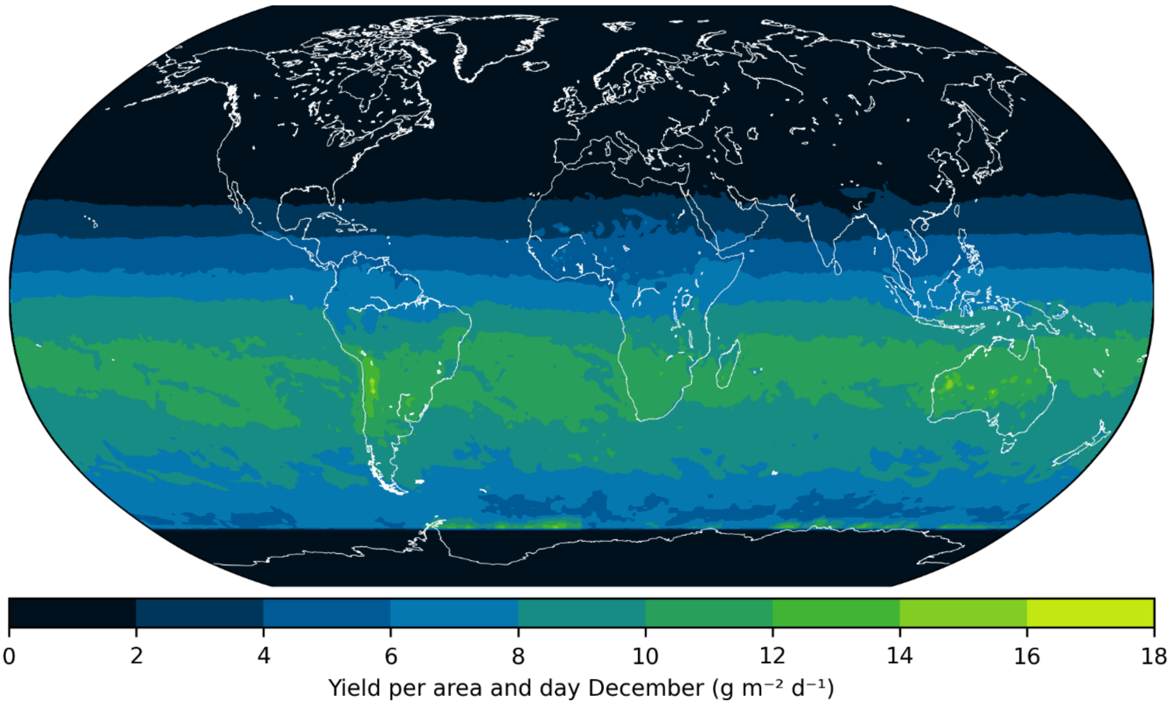

Figure S106. Monthly average yield per unit area and day for 3 mm diameter tubing.

9. Fourier-Transform Infrared Spectroscopy of Nanoparticles

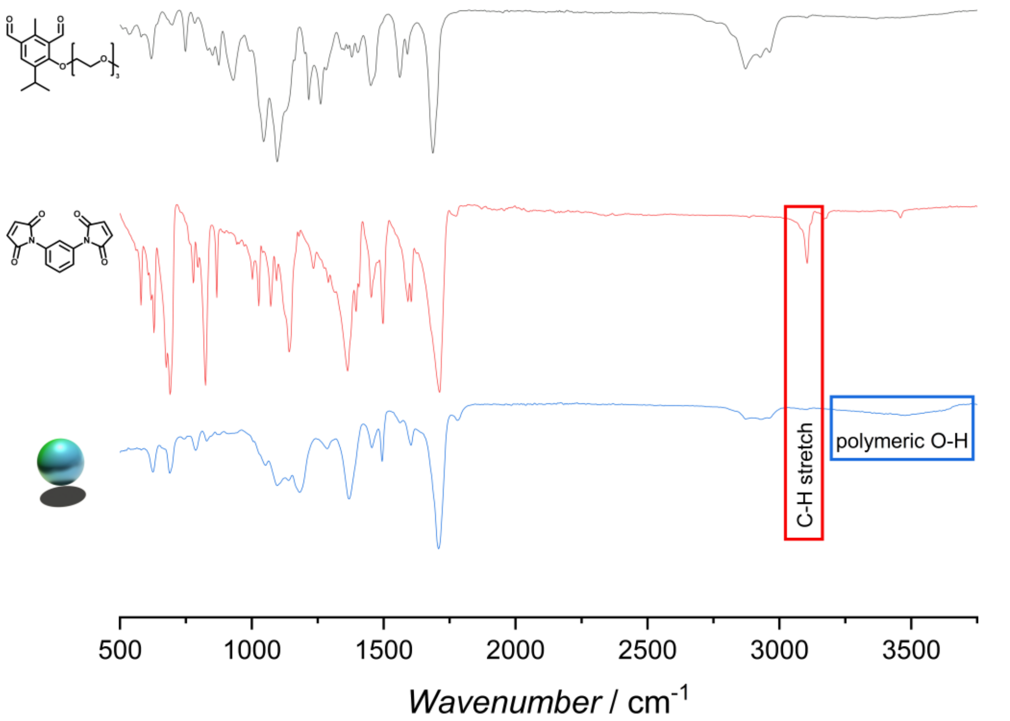

Figure S107. FT-IR spectra of AA (top), BB (middle), and the resultant microparticles (bottom).

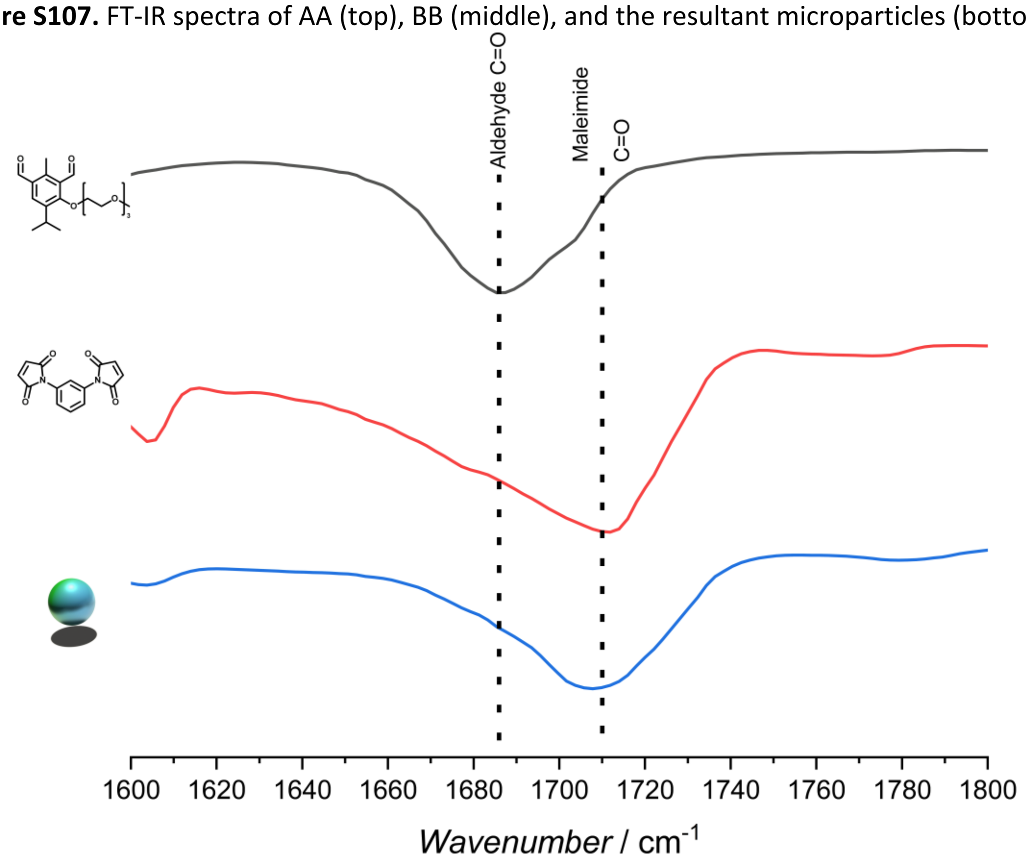

Figure S108. Enlarged FT-IR spectra of AA (top), BB (middle), and the microparticles (bottom).

10. NMR and DLS from irradiation with a 365 nm LED

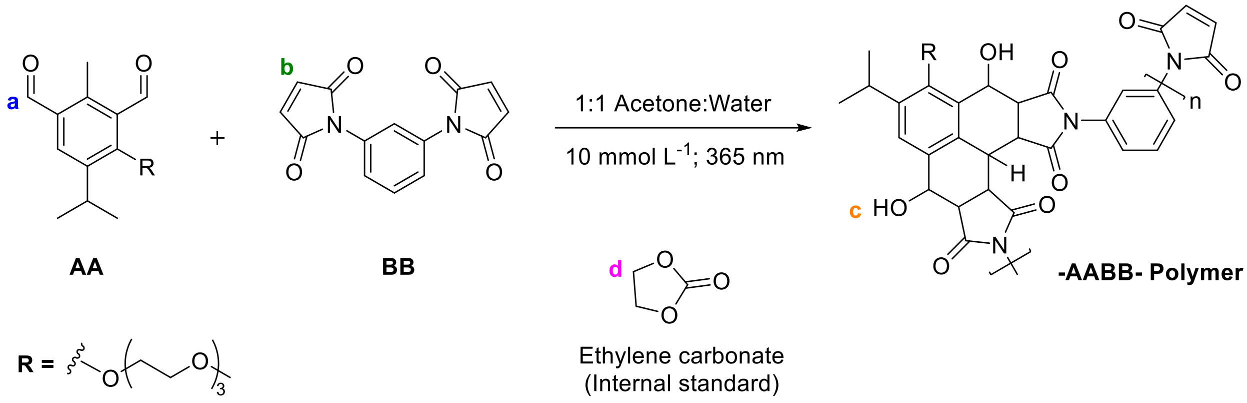

A 10 mmol L^{-1} equimolar solution of the AA and BB monomer was prepared in a 1:1 mixture of D_2O and acetone-d_6. Ethylene carbonate was added as internal standard. The solution was sonicated for 30 min at 45 °C, and 0.6 mL was filled either into an NMR tube (NMR experiment) or into a 0.8 mL clear flat base 8 mm crimp vial (DLS experiment), after having passed a 0.2 µm PTFE syringe filter. The solution was degassed with nitrogen for 5 min and subsequently irradiated using a Violumas High Power UVA LED COB (1.4 A forward current) with an emission centered at λ_{max} = 365 nm[11] (Figure S109). The NMR tube or crimp vial was positioned at a distance of 2 cm from the LED. The LED and the NMR tube or crimp vial were cooled by a fan to maintain ambient temperature. For the DLS experiment, small volumes were taken out of the reaction solution over time, and 10 µL of each sample was added to 1.3 mL Milli-Q water for DLS measurements. For the NMR experiment, the NMR tube was removed temporarily from the LED at given time points for the measurements over time.

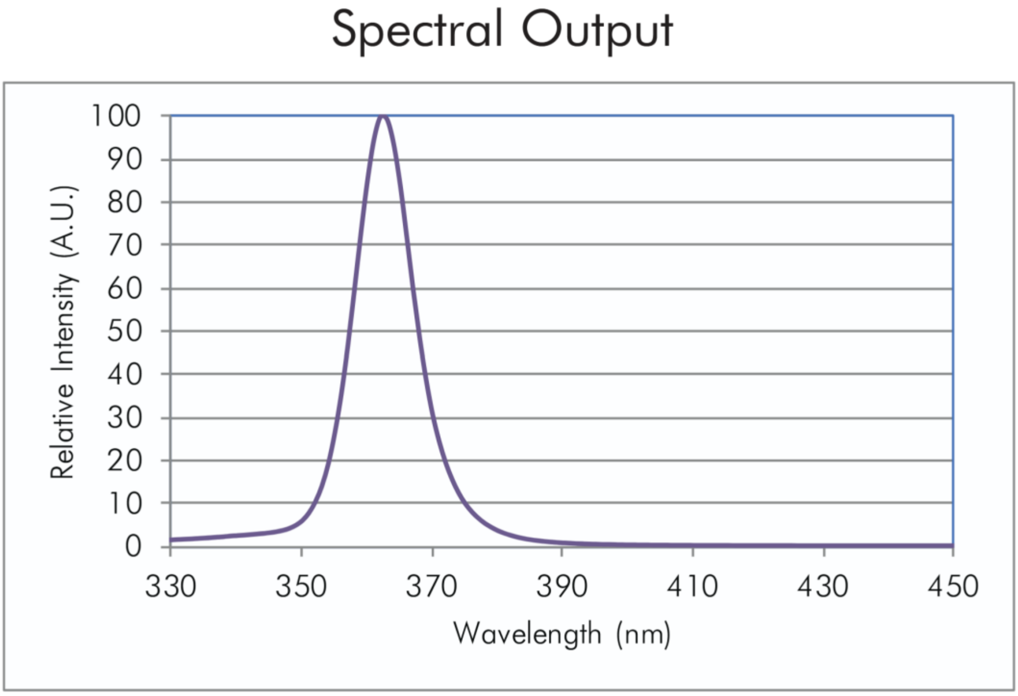

Figure S109. Spectral output of the employed Violumas High Power UVA LED COB with an emission

centered at λ_{max} = 365 nm.[11]

Calculation of the conversion from ^{1}H NMR spectroscopy:

The conversion X of the AA monomer based on the aldehyde resonance ***a*** (δ = 10.39 -10.55 ppm), of the BB monomer based on the double bond resonances ***b*** (δ = 6.90-7.03 ppm), and of the oligomers based on the hydroxy proton resonances ***c*** (δ = 5.39-5.94 ppm) were calculated in reference to the resonances ***d*** of the internal standard ethylene carbonate (δ = 4.44-4.59 ppm) based on the following equations:

$$X(\text{AA}) = 1 - \frac{\int a}{\int a(t_0)} \quad ; \quad X(\text{BB}) = 1 - \frac{\int b}{\int b(t_0)} \quad ; \quad X(\text{Oligomers}) = \frac{\int c}{2 \cdot \int a(t_0)}$$

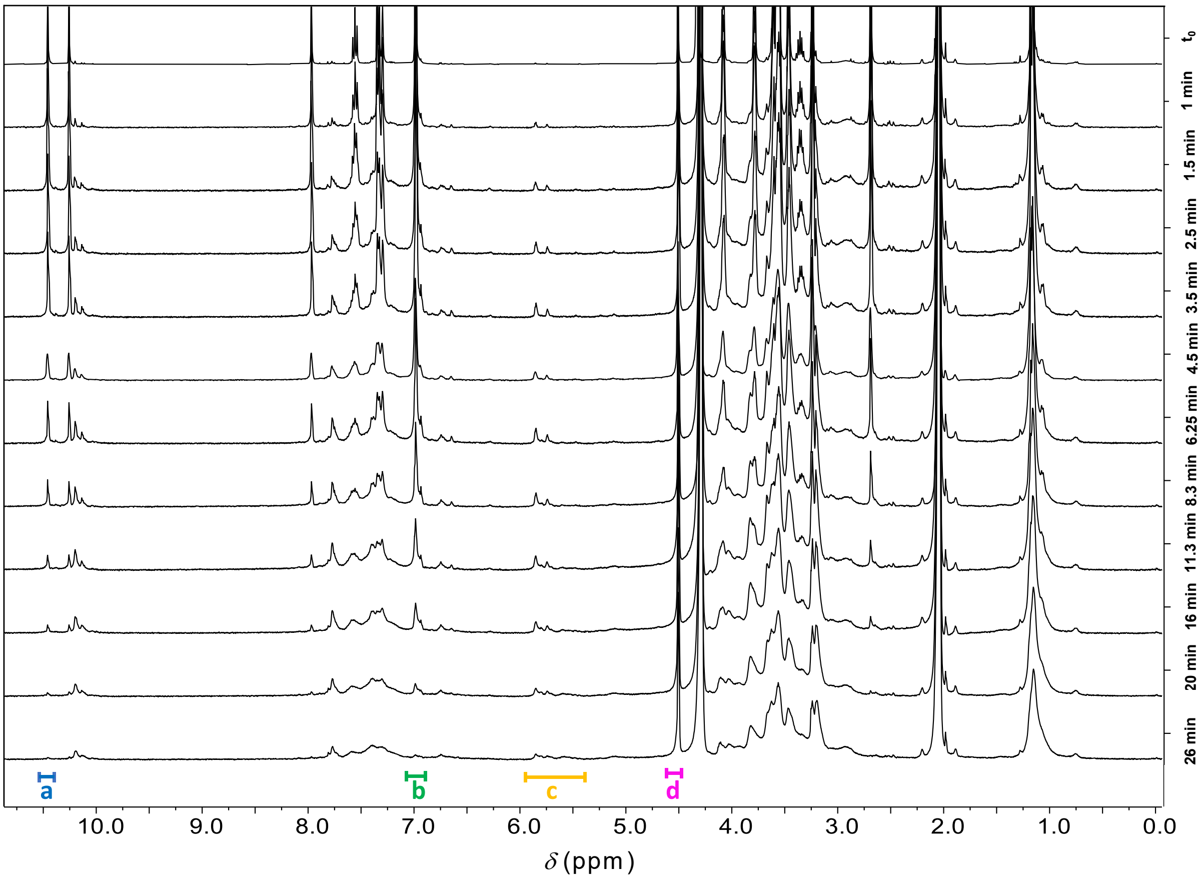

Figure S109. ^{1}H NMR spectra (400 MHz, D_1 = 2 s, NS = 32) over time in a 1:1 mixture of D_2O and acetone-d_6 for irradiation with a λ_{max} = 365 nm LED. The filtered and degassed solution was irradiated in the NMR tube at a distance of 2 cm from the LED.

Table S5. ^{1}H NMR spectroscopic data showing the integrals and conversions of the AA monomer, the BB monomer, and the oligomers over time for irradiation with a 365 nm LED in a 1:1 mixture of D_2O and acetone-d_6. The filtered and degassed solution was irradiated in the NMR tube at a distance of 2 cm from the LED.

Time (min)	$\int a$	$X(\text{AA})$ (%)	$\int b$	$X(\text{BB})$ (%)	$\int c$	$X(\text{Oligomers})$ (%)
0.00	0.79	0	3.28	0	0.00	0
0.30	0.64	19	2.52	23	0.06	4

1.00	0.55	30	2.22	32	0.10	6
1.50	0.50	37	2.08	37	0.13	8
2.50	0.43	45	1.85	44	0.17	11
3.50	0.35	55	1.54	53	0.20	13
4.50	0.27	65	1.19	64	0.24	15
6.25	0.23	71	1.09	67	0.28	17
8.33	0.16	80	0.68	79	0.30	19
11.33	0.10	87	0.42	87	0.31	20
16.00	0.06	92	0.32	90	0.35	22
20.00	0.05	94	0.19	94	0.30	19
26.00	0.03	96	0.10	97	0.27	17

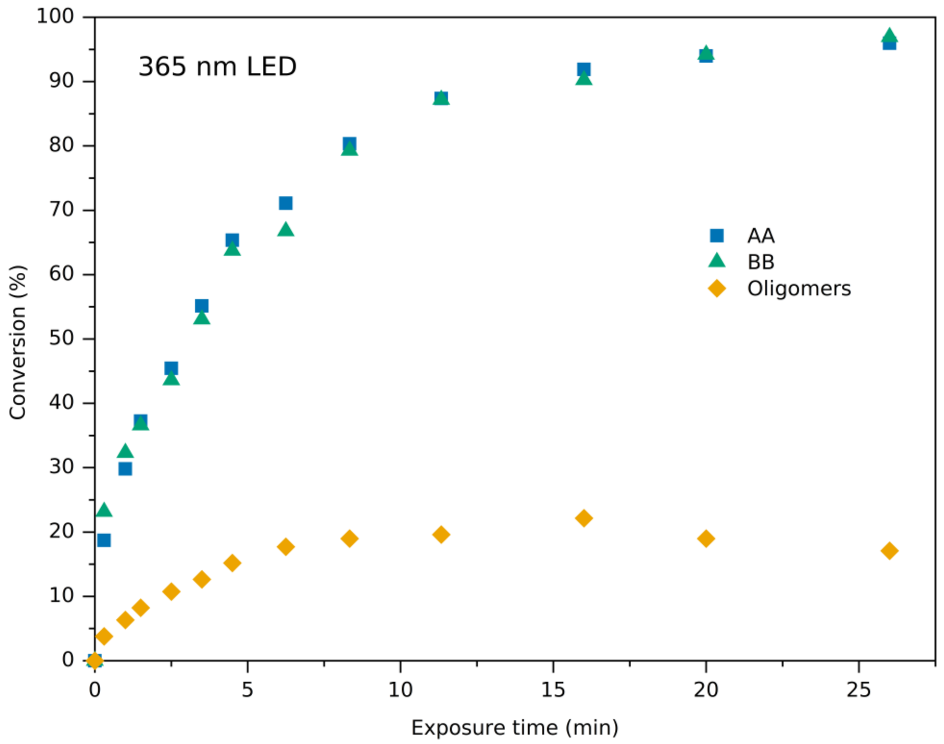

Figure S110. Conversion of the AA monomer, BB monomer and oligomers obtained from ^{1}H NMR in a 1:1 mixture of D_2O and acetone-d_6 for irradiation with a 365 nm LED. The filtered and degassed solution was irradiated in the NMR tube at a distance of 2 cm from the LED.

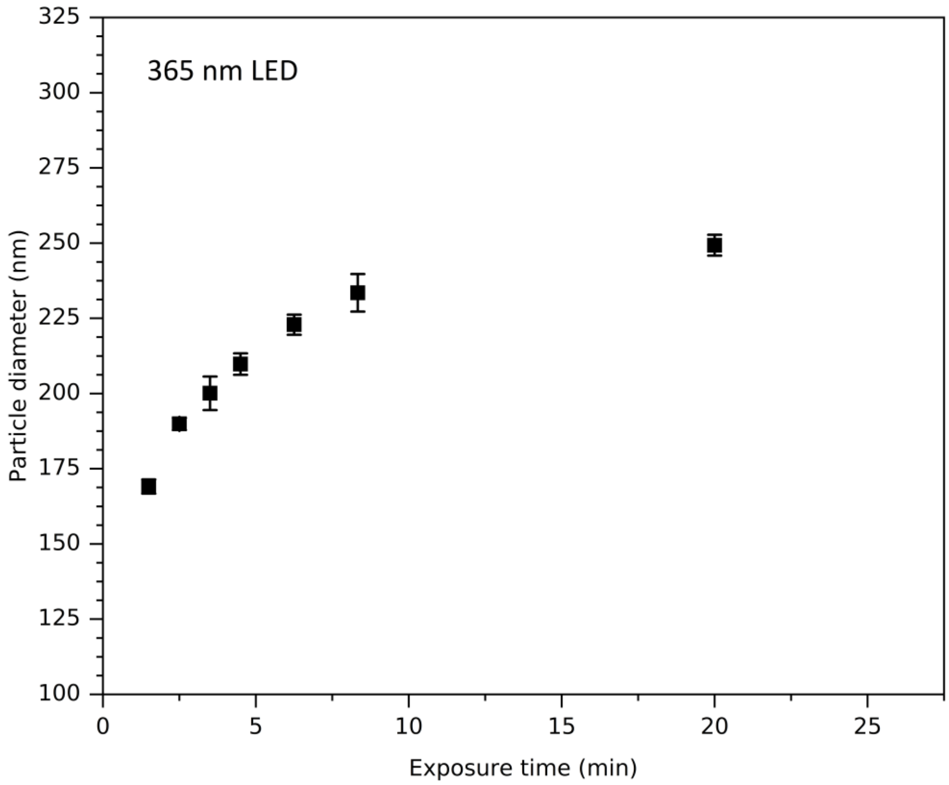

Figure S111. The z-averaged size of the particle diameters from DLS over time for irradiation with a365 nm LED. The filtered and degassed solution was irradiated in a 0.8 mL, 8 mm crimp vial at a distance of 2 cm from the LED.

11. Reynolds Number

The Reynolds number (*Re*) can be used to predict the transition of laminar to turbulent flow, with turbulent flow becoming relevant for flow in a pipe or tube at around Re_d > 2300. The Reynolds number for flow in a pipe or tube is calculated as: $Re_d = \frac{u \cdot d}{\nu}$ with *u*: flow speed (m s^{-1}), *d*: inner tube diameter (m), and ν: kinematic viscosity ($m^2 s^{-1}$). The value of the kinematic viscosity of a (nearly) 1:1 mixture of water and acetone was obtained from literature.[12] The highest Reynolds number among the 2 mm and 3 mm inner diameter tubes is reached for the 3 mm tube at the maximal flow speed of 30 m min^{-1} with Re_d = 907 at 20 °C (or Re_d = 1823 at 50 °C). Therefore, laminar flow is expected for all employed flow rates with the two tube diameters used.

12. Residence time distribution determined using UV/Vis spectroscopy

Residence time experiments were conducted in the Karlsruhe (Germany) installation of the sun flow reactor. The experiments were performed on the 3 mm inner diameter tubing. At the reactor inlet – behind the peristaltic pump – 0.5 mL of a 4.4 mM solution of neutral red in a 1:1 mixture of water and acetone was injected via a check-valve and T-piece as tracer into the continuous flow. In parallel, the absorbance at the exit of the reactor tube was measured using a 10 mm quartz glass high performance flow-through cell, an Ocean Insight DH-2000 light source, and an Ocean Insight OCEANFX miniature spectrometer. The OceanView 2.0.8 software was used to record the average absorbance for the

wavelengths of 518 and 532 nm over time, with an integration time of 50 ms (flow rates: 9.5 mL min^{-1}, 106.4 mL min^{-1},159.6 mL min^{-1}). The peristaltic pump was used without back pressure regulation. For the flow rate of 26.6 mL min^{-1}, the fluctuations in the flow speed from the peristaltic pump were more pronounced, so that the integration time was increased to 200 ms.

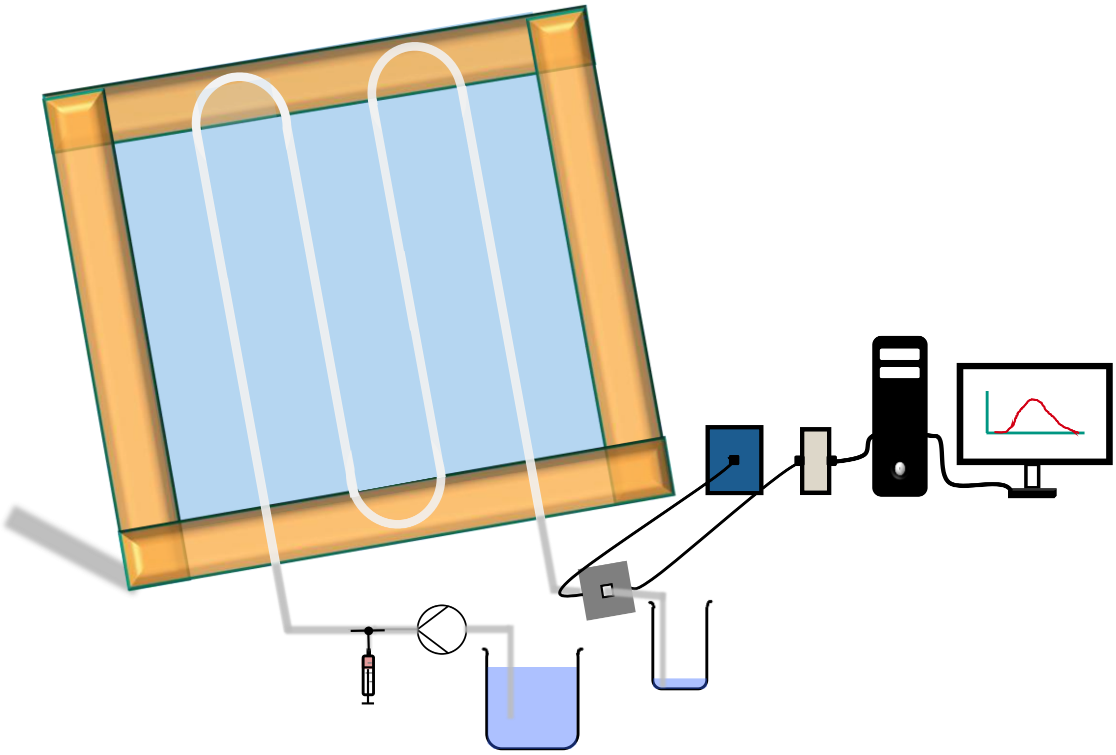

Figure S112. Experimental setup for the residence time distribution experiments.

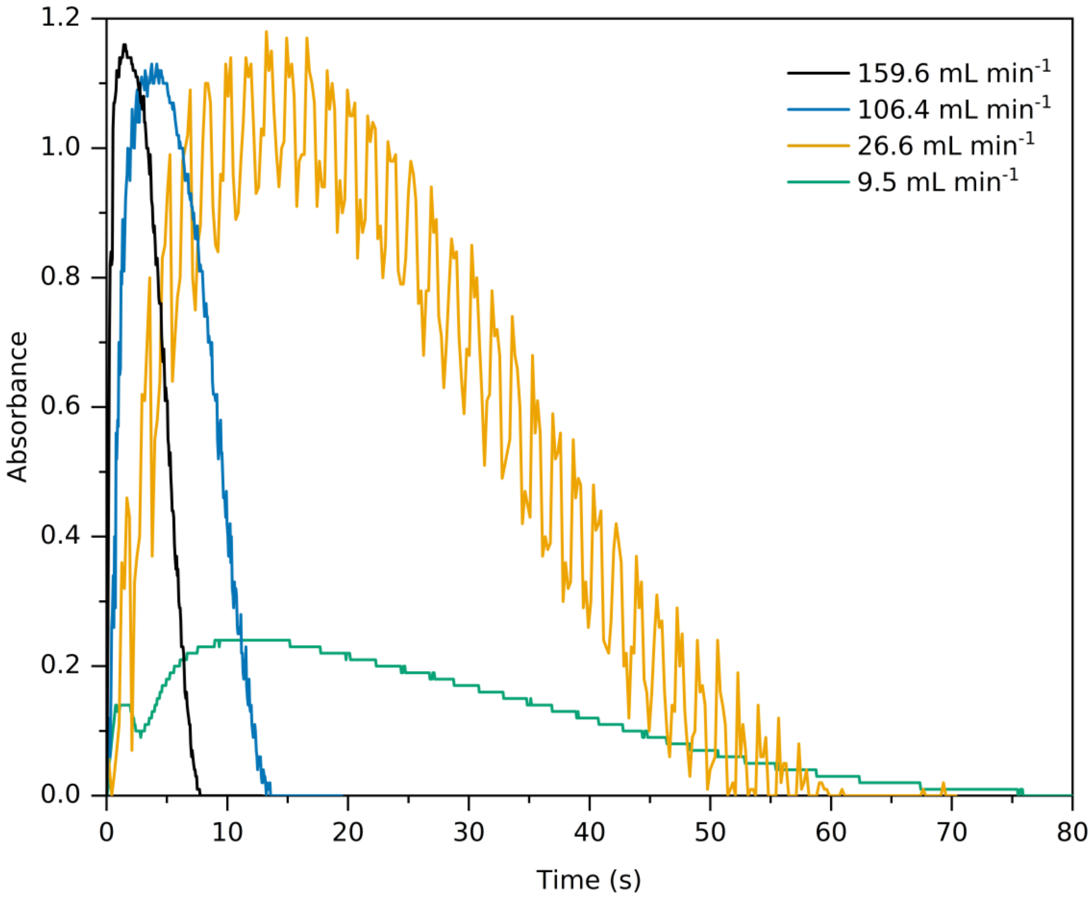

Figure S113. Residence time distribution curves at different flow rates for the 3 mm inner diameter tubing. The absorbance of the exiting tracer dye was measured over time, giving a signal proportional to the concentration $c(t)$. The asymmetric profile of the measured curves matches the expectation of laminar flow conditions. The leading and trailing humps in the curve for the lowest flow rate of 9.5 mL min^{-1} indicate convective and diffusion mass transfer processes, respectively. The plotted time axis was shifted for each flow rate to start with the beginning of detected absorbance.

The fluctuations in the absorbance signal are caused by the pressure pattern of the peristaltic pump. The area under the curve varies for the same amount of injected tracer, since different flow rates are recorded. For the three higher flow rates, the area under the curve becomes smaller with a higher flow rate as expected. In the experiment with the lowest flow rate, 9.5 mL min^{-1}, a larger portion of the dye was too diluted at the outlet of the reactor tube to give any signal in absorbance, so that the curve is shortened compared to the other flow rates.

The variances of the residence time distribution curves were determined from the raw data without curve fitting. To obtain variances that are comparable between different flow rates, dimensionless variances were calculated with $\sigma_\theta^2 = \frac{\sigma^2}{\tau^2}$. The mean residence times τ were obtained here by dividing the volume of the reactor by the volumetric flow rates.

Table S6. Variances σ^2 and dimensionless variances σ_θ^2 of the measured residence time distribution curves at the different flow rates for the 3 mm inner diameter tubing. A higher flow rate results in a narrower residence time distribution.

Flow rate (mL min^{-1})	σ^2 (s^2)	σ_θ^2
159.6	2.7	0.0121
106.4	8.0	0.0157
26.6	142.6	0.0175
9.5	-*	-*

*The variances for the lowest flow rate of 9.5 mL min^{-1} are not provided, since larger portions of the tracer dye remained undetected with the concentration at the outlet being lower than the detection limit.

According to the Hagen-Poiseuille law, which assumes the laminar flow of an incompressible, Newtonian fluid, the required pressure difference Δp at the two ends of a tube with the length ΔL and the radius R to drive a fluid with the dynamic viscosity η and the volumetric flow rate $\dot{V}$ can be calculated from:

$$\Delta p = \frac{8\eta \cdot \Delta L}{\pi R^4}\dot{V}$$

Table S7. Pressure differences Δp in both Pa and mbar for the 2 mm and 3 mm inner diameter tubing with a length $\Delta L = 5.6$ m for a (nearly) 1:1 mixture of water and acetone (η = 1.5485 mPa s at 20 °C)[12] at different flow rates using the Hagen-Poiseuille law (inlet and outlet pressure drops not considered here).

Flow rate (mL min^{-1})	Δp for 2 mm tubing (Pa)	Δp for 2 mm tubing (mbar)	Δp for 3 mm tubing (Pa)	Δp for 3 mm tubing (mbar)
0.37	136	1	27	0.3
2.39	880	9	174	2
7.18	2642	26	522	5
11.97	4405	44	870	9
47.89	17625	176	3482	35
95.78	35250	353	6963	70
159.6	58738	587	11603	116
212.8	78318	783	15470	155

13. Particle stability

To assess the stability of the particles produced under various conditions, an assay of their stability in different solvents was conducted. Under four different conditions that were selected to broadly represent a breadth of solvent conditions: DCM at room temperature, water at room temperature, p-xylene at room temperature, and p-xylene at 100°C, an identical batch of particles was exposed for 48 hours. Subsequently, the particles were dried under reduced pressure at 40°C for two weeks. SEM analysis of the resultant particles reveals minimal coagulation or damage to the particles, indicating the high stability under harsh conditions.

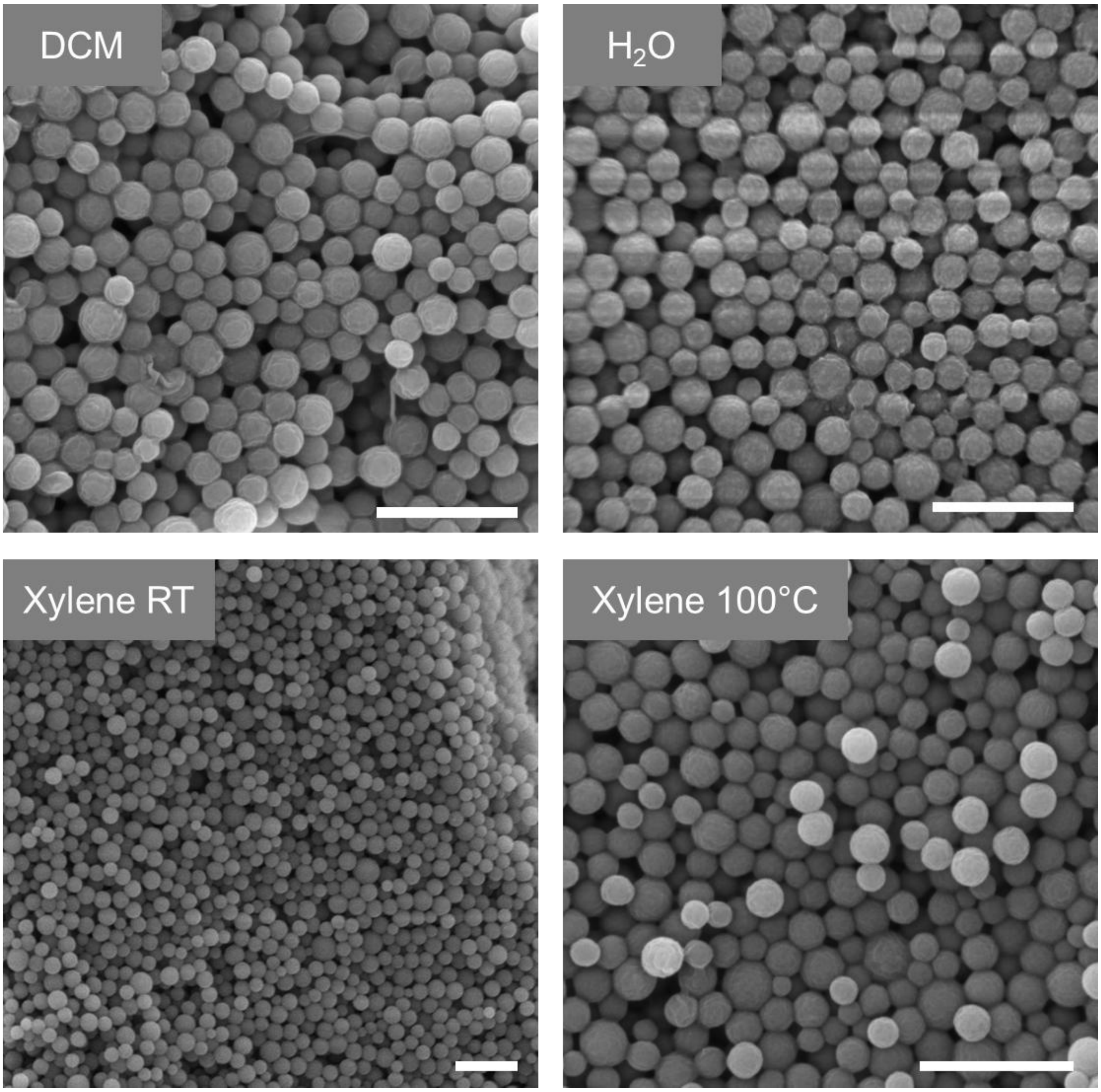

Figure S114. SEM images of the particles after exposure to harsh solvent conditions. Scale bars = 1 µm

14. Reactor fouling

To eliminate concerns of reactor fouling due to polymer deposition, we analysed the experiments conducted chronologically, and observed no significant trend over time. Therefore, we conclude that polymer deposition on the reactor walls, or other fouling mechanisms do not have a long-term fouling effect on the overall reactor performance. The data is provided below.

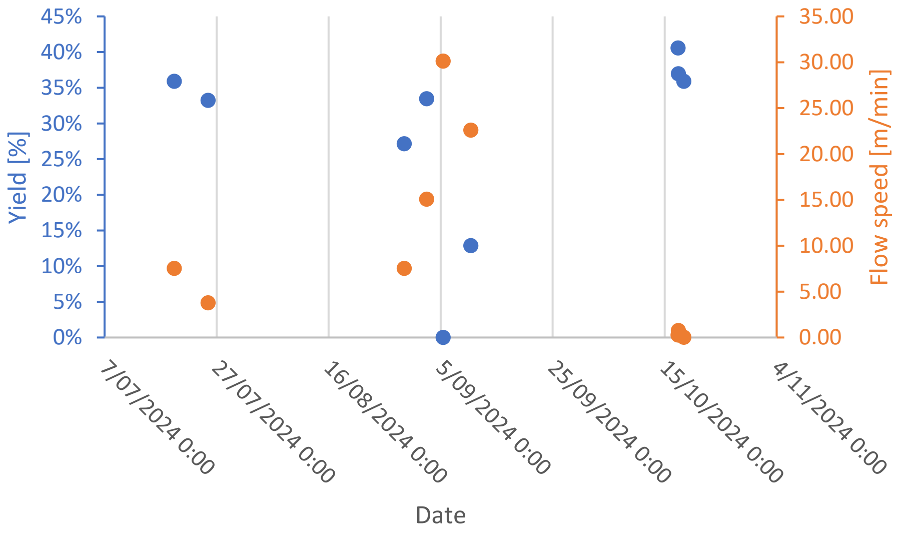

Figure S115. Yield and flow speed vs experiment date of the 3 mm tubing.

15. References

[1] J. A. Kammerer, F. Feist, D. Ryklin, A. Sarkar, C. Barner-Kowollik, R. R. Schröder, *Advanced Materials* **2023**, 2211074.

[2] J. Schindelin, I. Arganda-Carreras, E. Frise, V. Kaynig, M. Longair, T. Pietzsch, S. Preibisch, C. Rueden, S. Saalfeld, B. Schmid, J.-Y. Tinevez, D. J. White, V. Hartenstein, K. Eliceiri, P. Tomancak, A. Cardona, *Nat Methods* **2012**, *9*, 676–682.

[3] C. A. Schneider, W. S. Rasband, K. W. Eliceiri, *Nat Methods* **2012**, *9*, 671–675.

[4] N. Raval, R. Maheshwari, D. Kalyane, S. R. Youngren-Ortiz, M. B. Chougule, R. K. Tekade, in *Basic Fundamentals of Drug Delivery*, Elsevier, **2019**, pp. 369–400.

[5] J. O. Holloway, L. Delafresnaye, E. M. Cameron, J. A. Kammerer, C. Barner-Kowollik, *Mater. Horiz.* **2024**, *11*, 3115–3126.

[6] “Ultraviolet radiation index | ARPANSA,” can be found under https://www.arpansa.gov.au/our-services/monitoring/ultraviolet-radiation-monitoring/ultraviolet-radiation-index, **2025**.

[7] "UV index - Federal Office for Radiation Protection (BfS)," can be found under https://www.bfs.de/EN/topics/opt/uv/index/current/current-daily-graphs.html, **2025**.

[8] S. Ong, C. Campbell, P. Denholm, R. Margolis, G. Heath, *Land-Use Requirements for Solar Power Plants in the United States*, **2013**.

[9] “NASA POWER | Prediction Of Worldwide Energy Resources,” can be found under https://power.larc.nasa.gov/, **2025**.

[10] A. Kobyshev, H. Bertrand, I. Randolf, J. Vandenberg, K. Stopa, M. Yasirroni, Matthias, “SunTime, Version 1.3.2,” can be found under https://github.com/SatAgro/suntime, **2025**.

[11] "VC2X2C45L9-365-Rev080621 | Violumas," can be found under https://violumas.com/wp-content/uploads/2024/02/VC2X2C45L9-365-Rev080621.pdf, **2025**.

[12] K. S. Howard, R. A. McAllister, “The viscosity of acetone-water solutions up to their normal boiling points” *AIChE Journal* **1958**, *4*, 362–366.
